# Individual and joint performance of DNA methylation profiles, genetic risk score and environmental risk scores for predicting breast cancer risk

**DOI:** 10.1002/1878-0261.12594

**Published:** 2019-11-19

**Authors:** Zhong Guan, Janhavi R. Raut, Korbinian Weigl, Ben Schöttker, Bernd Holleczek, Yan Zhang, Hermann Brenner

**Affiliations:** ^1^ Division of Clinical Epidemiology and Aging Research German Cancer Research Center (DKFZ) Heidelberg Germany; ^2^ Medical Faculty Heidelberg University of Heidelberg Germany; ^3^ Division of Preventive Oncology German Cancer Research Center (DKFZ) National Center for Tumor Diseases (NCT) Heidelberg Germany; ^4^ German Cancer Consortium (DKTK) German Cancer Research Center (DKFZ) Heidelberg Germany; ^5^ Network Aging Research University of Heidelberg Germany; ^6^ Saarland Cancer Registry Saarbrücken Germany

**Keywords:** breast cancer, DNA methylation, environmental risk, genetic risk, risk prediction model, SNPs

## Abstract

DNA methylation patterns in the blood, genetic risk scores (GRSs), and environmental risk factors can potentially improve breast cancer (BC) risk prediction. We assessed the individual and joint predictive performance of methylation, GRS, and environmental risk factors for BC incidence in a prospective cohort study. In a cohort of 5462 women aged 50–75 from Germany, 101 BC cases were identified during 14 years of follow‐up and were compared to 263 BC‐free controls in a nested case–control design. Three previously suggested methylation risk scores (MRSs) based on methylation of 423, 248, and 131 cytosine‐phosphate‐guanine (CpG) loci, and a GRS based on the risk alleles from 269 recently identified single nucleotide polymorphisms were constructed. Additionally, multiple previously proposed environmental risk scores (ERSs) were built based on environmental variables. Areas under the receiver operating characteristic curves (AUCs) were estimated for evaluating BC risk prediction performance. MRS and ERS showed limited accuracy in predicting BC incidence, with AUCs ranging from 0.52 to 0.56 and from 0.52 to 0.59, respectively. The GRS predicted BC incidence with a higher accuracy (AUC = 0.61). Adjusted odds ratios per standard deviation increase (95% confidence interval) were 1.07 (0.84–1.36) and 1.40 (1.09–1.80) for the best performing MRS and ERS, respectively, and 1.48 (1.16–1.90) for the GRS. A full risk model combining the MRS, GRS, and ERS predicted BC incidence with the highest accuracy (AUC = 0.64) and might be useful for identifying high‐risk populations for BC screening.

## Graphical abstract

DNA methylation, and genetic and environmental risk scores (MRS, GRS, ERS) have been suggested for risk stratification in breast cancer (BC) screening. Their individual and joint performance for predicting BC risk were assessed in a prospective cohort study. Prediction accuracy was better for GRS than for MRS and ERS and highest for the combination of all three risk scores.

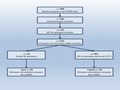

**Abbreviations**
AUC: area under the receiver operating characteristic curve
BC: breast cancer
CpG: cytosine‐phosphate‐guanine
ERS: environmental risk score
GRS: genetic risk score
MRS: methylation risk score
SNP: single nucleotide polymorphism

## 1 Introduction

Breast cancer (BC) is the most commonly diagnosed cancer and the leading cause of cancer death among women worldwide, accounting for nearly 2.08 million new cases and 630 000 deaths in 2018 (Bray *et al.*, 2018). Among women aged 50–69 years, the detection of early‐stage disease through mammography has led to a decline in BC mortality (Independent, 2012). Although mammography is widely used for BC screening, it has limitations such as high rates of false‐positive results and overdiagnosis (Independent, 2012; Pace and Keating, 2014). Furthermore, screening offers so far (except for women who had family history of BC or mutation in *BRCA1* or *BRCA2*) do not take interindividual variation of BC risk into account (Winters *et al.*, 2017).

DNA methylation markers detected in whole blood have emerged as potential candidates for the identification of high‐risk populations for earlier or further specific BC screening in recent years (Guan *et al.*, 2018). While these markers have been identified in diverse study populations through different approaches (Joo *et al.*, 2018; van Veldhoven *et al.*, 2015; Xu *et al.*, 2013; Xu *et al.*, 2019; Yang *et al.*, 2019), their predictive value in prospectively collected samples needs to be further validated. Additionally, genome‐wide association studies (GWASs) have identified an increasing number of single nucleotide polymorphisms (SNPs) that are independently associated with BC risk (Mavaddat *et al.*, 2015; Michailidou *et al.*, 2015; Michailidou *et al.*, 2017). Although these SNPs confer small risk individually, their combined effect can substantially influence BC risk. Genetic risk scores (GRSs) based on multiple SNPs can be used to stratify women according to their risk of developing BC which may lead to more refined, personalized prevention strategies (Burton *et al.*, 2013; Mavaddat *et al.*, 2015). Recently, Mavaddat *et al. *(2019) constructed a GRS based on 313 SNPs which discriminated BC cases from controls with an area under the receiver operating characteristic curve (AUC) of 0.63 in prospective studies conducted among white European populations. Besides epigenetic and genetic factors, reproductive (e.g., menarche, pregnancy, and menopause), lifestyle (e.g., alcohol consumption), and anthropometric (e.g., body mass index) factors, as well as the use of hormone medications (Madigan *et al.*, 1995; Peto, 2011), have long been identified to be related to BC risk. Risk prediction models combining these known risk factors in ‘environmental risk scores’ (ERSs) have been developed (Dierssen‐Sotos *et al.*, 2018; Gail *et al.*, 1989; Maas *et al.*, 2016; Novotny *et al.*, 2006; Park *et al.*, 2013; Rudolph *et al.*, 2018; Wang *et al.*, 2016), but their predictive accuracy was found to be modest (Anothaisintawee *et al.*, 2012).

In this study, we aimed to simultaneously assess the individual and joint performance of whole‐blood DNA methylation markers, GRS, and ERS for BC incidence in a prospective cohort study.

## 2 Materials and methods

### 2.1 Study population and data collection

**Figure 1:**
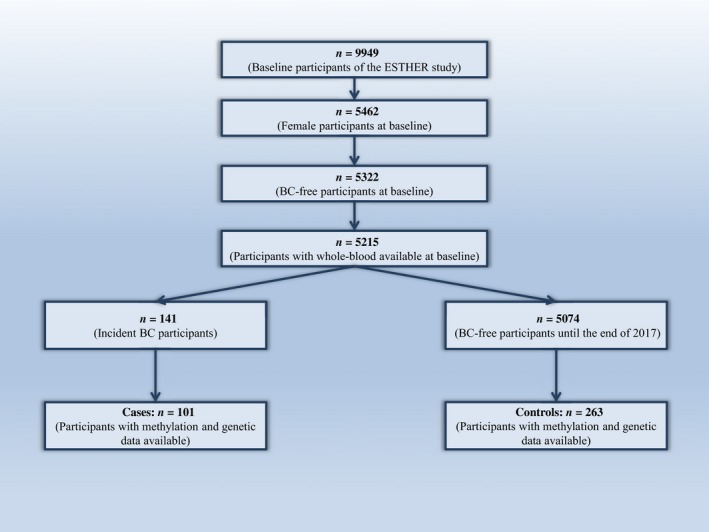
Flow diagram of inclusion of study participants.

We performed a case–control study nested within the ESTHER (Epidemiologische Studie zu Chancen der Verhütung, Früherkennung und optimierten Therapie chronischer Erkrankungen in der älteren Bevölkerung) cohort, a population‐based study, conducted in Saarland, Germany. Details of the ESTHER cohort have been previously described (Schottker *et al.*, 2013). As shown in Fig. 1, 9949 older adults aged 50–75 years of whom 5462 were women were recruited by their general practitioners during routine health checkups between July 2000 and December 2002, and followed up thereafter. The participants completed a standardized self‐administered questionnaire (collecting information on sociodemographic, reproductive, and lifestyle factors) and donated blood samples at baseline. Prevalent and incident cancer was determined by self‐report and record linkage with the Saarland Cancer Registry. DNA methylation measurements and genotyping were performed in the baseline blood samples of the ESTHER participants. Overall, we identified 101 women with incident BC and 263 women without BC at baseline or during the follow‐ups for whom both DNA methylation and genetic data were available. The study was approved by the ethics committees of the University of Heidelberg and of the state medical board of Saarland, Germany. Written informed consent was provided by all participants.

### 2.2 Methylation assessments

Blood samples collected at baseline (available for 98.8% of participants) were stored at – 80 °C until further processing. DNA was extracted from whole‐blood samples using a salting out procedure (Miller *et al.*, 1988). DNA methylation levels of 866836 cytosine‐phosphate‐guanine (CpG) loci were quantified by the Infinium Methylation EPIC (850K) BeadChip Assay (Illumina Inc., San Diego, CA, USA) (Zaimi *et al.*, 2018). Briefly, 1 µg DNA was bisulfite converted, and 250 ng bisulfite‐treated DNA was applied to the EPIC BeadChips following the manufacturer’s instruction. GenomeStudio® (version 2011.1; Illumina Inc.) was used to extract DNA methylation signals from the scanned arrays (module version 1.9.0; Illumina Inc.). Due to its straightforward biological interpretation, the methylation level of a specific CpG site was quantified as a β‐value ranging from 0 (no methylation) to 1 (full methylation) (Du *et al*., 2010; Xie *et al*. 2019). Illumina normalization and preprocessing methods implanted in Illumina’s GenomeStudio® were utilized. Data were normalized to internal controls provided by the manufacturer. All controls were checked for inconsistencies in each measured plate. Probes with a detection *P*‐value > 0.01 or with missing value > 10% of our samples were excluded from analysis (Zhang *et al.*, 2017). Leukocyte composition was estimated using the algorithms of Houseman et al. (Houseman *et al.*, 2012).

### 2.3 Genotyping

Extracted DNA from whole blood was genotyped using the Illumina Infinium OncoArray BeadChip (Illumina). General genotyping quality control assessment was done as previously described (Anderson *et al.*, 2010). Genotypes for common variants across the genome were imputed using data from UK10K‐1000 Genomes Project (phase 3, October 2014) with impute v2.3.2 (https://mathgen.stats.ox.ac.uk/impute/impute_v2.html#download) after prephasing with shapeit software v2.12 (https://mathgen.stats.ox.ac.uk/genetics_software/shapeit/shapeit.html#download). Thresholds were set for imputation quality to retain both common and rare variants for validation. Poorly imputed SNPs were defined by an information metric *I* < 0.70 and excluded for the subsequent analysis. All genomic locations are given in NCBI Build 37/UCSC hg19 coordinates. All SNPs with a minor allele frequency (MAF) < 1% were excluded. After imputation, the SNP set consisted of 9 198 808 genotyped and imputed SNPs. PLINK v1.90b5.4 was then used to extract SNPs for the required regions of interest (Chang *et al.*, 2015).

### 2.4 Statistical analysis

#### 2.4.1 Methylation risk scores (MRSs)

We constructed MRSs based on three sets of CpGs. The first set included 450 CpGs associated with BC risk that were identified using genetic variants as instrument (Yang *et al.*, 2019). The second set included 280 BC‐related CpGs identified in previous prospective epigenome‐wide association studies (Joo *et al.*, 2018; van Veldhoven *et al.*, 2015; Xu *et al.*, 2013). The third set included 144 CpGs which was identified and validated in independent prospective studies (Xu *et al.*, 2019). CpGs with missing value > 10% in our sample were excluded from subsequent analyses. Furthermore, CpGs only included in the Infinium Methylation 27K BeadChip Array or the Infinium Methylation 450K BeadChip Array but not the Infinium EPIC Array were excluded, which left 423 CpGs from the first set (423‐CpGs), 248 CpGs from the second set (248‐CpGs), and 131 CpGs from the third set (131‐CpGs). The individual CpGs and their associations with BC risk (none of which was statistically significant after adjustment for multiple testing by the Benjamini–Hochberg method) are provided in Table S1. These CpGs were classified into hyper‐ and hypomethylated CpGs according to the previously reported relationship with BC (Joo *et al.*, 2018; van Veldhoven *et al.*, 2015; Xu *et al.*, 2013; Xu *et al.*, 2019; Yang *et al.*, 2019). MRSs were calculated as the sum of hypermethylated CpGs with methylation levels in the upper quartile of the distribution among controls, and of hypomethylated CpGs with methylation levels in the lower quartile of the distribution among controls.

#### 2.4.2 Genetic risk score (GRS)

A newly established set of 313 SNPs which discriminated BC cases from controls with a modest AUC = 0.63 (95% CI: 0.63–0.65) in recent GWAS was used to build GRS (Mavaddat *et al.*, 2019). SNPs were excluded from further analyses if they were missing in > 10% of our sample after imputation, if they were in high linkage disequilibrium (*D*' ≥ 0.95 and *r* ^2 ^> 0.8) with each other, or if the MAF was low (< 1%). Specifically, we searched the website (proxy SNP website: https://ldlink.nci.nih.gov/) to find surrogates for the missing SNPs. By setting the criteria of D' ≥ 0.95, *r* ^2 ^> 0.8, MAF ≥ 1%, distance < 250K bp, and missing value < 10% of our sample, only six SNPs (rs6656241, rs3791976, rs3008281, rs28489579, rs521667, and rs965352) could be used as surrogates for the six missing SNPs (rs56168262, rs3791977, rs66823261, rs4774565, rs527616, and rs6030585) in the ESTHER study. This resulted in inclusion of 269 SNPs in the GRS. GRSs for all eligible participants were calculated using the formula: GRS = β_1_X_1_ + β_2_X_2_+ … β_k_X_k_ … + β*_n_*X*_n_*_,_ where β_k_ is the per‐allele log odds ratio (OR) for BC associated with the risk allele for SNP k reported in the previous independent GWAS (Mavaddat *et al.*, 2019), X_k_ is the number of risk alleles for the same SNP (0, 1, or 2), and *n* is the total number of SNPs. SNPs and corresponding effect sizes for risk alleles reported in the derivation of the GRS (Mavaddat *et al.*, 2019) are summarized in Table S2.

#### 2.4.3 Environmental risk scores (ERSs)

For building ERSs, we conducted a literature search to identify previously published ERSs used for BC risk prediction. We identified and included seven risk scores (Dierssen‐Sotos *et al.*, 2018; Gail *et al.*, 1989; Maas *et al.*, 2016; Novotny *et al.*, 2006; Park *et al.*, 2013; Rudolph *et al.*, 2018; Wang *et al.*, 2016), including two scores from two multicenter studies (study populations were mainly from Europe, the United States, and Australia) and one each from the United States, Czech, South Korea, China, and Spain. Four of the seven risk scores were built according to the previously reported score prediction algorithms (Gail *et al.*, 1989; Novotny *et al.*, 2006; Park *et al.*, 2013; Wang *et al.*, 2016). The remaining three scores (Dierssen‐Sotos *et al.*, 2018; Maas *et al.*, 2016; Rudolph *et al.*, 2018) were derived from beta coefficients of the corresponding risk factors which were reported from multivariable logistic regression used in each study. The proportion of missing values for all variables including in the ERSs was below 5%, and the missing baseline values were imputed by mean value of each incomplete variable [age at menarche, age at first live birth, parity, menopausal status, age at menopause, current use of menopause hormone therapy (MHT)] within groups of cases and controls, respectively. In case the variables were not available in our data sets and could not be replaced, we built the risk scores without them. Algorithms applied to obtain the risk scores are summarized in Table S3.

#### 2.4.4 Descriptive analyses

Main characteristics of cases and controls were described using frequencies for categorical variables, and means and standard deviations (SDs) for continuous variables. The correlations between the risk scores were estimated by Pearson's correlation coefficients.

#### 2.4.5 Associations of MRS, GRS, and ERS with breast cancer risk

Crude associations of MRS, GRS, and ERS with BC risk were assessed by unconditional logistic regression with adjustment for leukocyte composition in case of MRS in Model I. In Model II, associations were adjusted for the complementary risk scores. Risk estimates for each risk score were included in the models either as quartiles (lowest quartile defined as the reference category) or as continuous variables (calculating ORs for an increase in risk scores by 1 SD). The individual association of previously identified CpGs with BC incidence was also estimated by unconditional logistic regression with adjustment for age, batch effects, and leukocyte composition. Areas under the receiver operating characteristic curves (AUCs) were estimated for evaluating the performance of the three types of risk scores in BC risk prediction. In addition to exploring the predictive value of risk scores over the entire period of follow‐up, analyses were repeated with follow‐up time restricted to the initial 7 years after recruitment or to subsequent years, respectively. Tenfold cross‐validation was employed to correct for potential overoptimism in prediction models. In 10‐fold cross‐validation, the original sample was randomly partitioned into 10 subsamples, the nine subsamples were then used as training data for the derivation of the prediction model and the remaining single subsample was used as the validation data for testing the model (Lanfear *et al.*, 2017). This process was repeated 1000 times. Additionally, the goodness of fit of the combined model was tested using the Hosmer–Lemeshow test to evaluate the calibration of multiple risk scores and using the tailed‐based test by Song et al (Song *et al.*, 2015). All statistical analyses were carried out in SAS 9.4 (SAS Institute, Cary, NC, USA), and 2‐sided *P*‐values < 0.05 were considered statistically significant.

## 3 Results

Table 1 presents the baseline characteristics of the study population. Of 5462 women aged 50–75 years recruited between July 2000 and December 2002, a total of 101 BC cases and 263 controls were included for whom both methylation and genomic data were available for the current analysis (Fig. 1). The median time to diagnosis for cases, defined as the time between recruitment/sample collection and BC diagnosis ranged from 0.24 months to 13.6 years [median (interquartile range): 6.8 (3.7–8.9) years]. Mean age was about 61 years for both cases and controls. The distributions of participant characteristics were similar between cases and controls except for age at menopause and parity. Controls were more likely to have had an early menopause and to have a higher parity.

**Table 1:** Main characteristics of the study population at the time of cohort recruitment. Numbers shown were drawn from the not imputed data set. Missing values in cases/controls: age at menarche 3/3, age at first live birth 4/4, parity 5/5, menopausal status 3/3, age at menopause 4/3, and current use of MHT 2/3. No., number.

Characteristics	Cases (*n* = 101) No. (%)	Controls (*n* = 263) No. (%)	*P*‐value
Age (years)			
Mean ± SD	60.6 ± 6.5	60.8 ± 6.0	0.80
50–59	45 (44.6)	105 (39.9)	0.54
60–69	47 (46.5)	139 (52.9)	
≥ 70	9 (8.9)	19 (7.2)	
Age at menarche^a^			
≤ 12	22 (22.4)	50 (19.2)	0.45
12–14	53 (54.1)	150 (57.7)	
≥ 15	23 (23.5)	60 (23.1)	
Age at first live birth^a^			
≤ 20	18 (18.6)	68 (26.3)	0.26
21–29	64 (66.0)	164 (63.3)	
≥ 30	15 (15.4)	27 (10.4)	
Parity^a^			
0	9 (9.4)	14 (5.4)	0.03*
1	28 (29.2)	61 (23.6)	
2	38 (39.6)	98 (38.0)	
≥ 3	21 (21.8)	85 (33.0)	
Ever breastfed			
No	46 (45.5)	112 (42.6)	0.69
Yes	55 (54.5)	151 (57.4)	
Menopausal status^a^			
Premenopausal	11 (11.1)	39 (15)	0.33
Postmenopausal	88 (88.9)	221 (85.0)	
Age at menopause^a^			
< 50	49 (50.5)	159 (61.2)	0.04*
≥ 50	48 (49.5)	101 (38.8)	
Ever use of oral contraceptive			
No	31 (31.0)	101 (39.2)	0.27
Yes	69 (69.0)	157 (60.8)	
Current use of MHT^a^			
No	35 (35.0)	113 (43.5)	0.13
Yes	65 (65.0)	147 (56.5)	
BMI (kg·m^−2^)			
≤ 25	30 (29.7)	86 (32.7)	0.86
25–30	40 (39.6)	100 (38.0)	
> 30	31 (30.7)	77 (29.3)	
Height (m)			
≤ 1.60	44 (43.6)	104 (39.5)	0.85
1.60–1.65	30 (29.7)	90 (34.2)	
1.65–1.70	15 (14.6)	37 (14.1)	
> 1.70	12 (11.9)	32 (12.2)	
Alcohol consumption (g·day^−1^)			
0	49 (48.5)	109 (41.4)	0.31
≤ 5	16 (15.8)	62 (23.6)	
5–30	29 (28.7)	77 (29.3)	
> 30	7 (7.0)	15 (5.7)	
Smoking status			
Never smoker	63 (64.3)	166 (63.8)	0.52
Former smoker	16 (19.3)	49 (18.8)	
Current smoker	19 (15.4)	45 (17.4)	
Physical activity			
< once/week	21 (20.8)	68 (25.9)	0.50
≥ once/week	80 (79.2)	194 (74.1)	
Sleep quality			
Good	87 (91.6)	215 (90.3)	0.62
Common	6 (6.3)	15 (6.3)	
Poor	2 (2.1)	8 (3.4)	
No. of FDRs with cancer history			
0	50 (51.5)	119 (46.5)	0.68
1	35 (36.1)	89 (34.8)	
≥ 2	12 (12.4)	48 (18.7)	
No. of FDRs with BC			
0	93 (92.1)	239 (90.9)	0.06
≥ 1	8 (7.9)	24 (9.1)	

a Numbers do not add to total numbers due to missing values.

* *P* ‐value < 0.05.

Table 2 provides an overview of risk factors included in the previously proposed ERS and the performance of these ERSs in our study. The most commonly included environmental risk factors were age, age at menarche, age at first live birth, BMI, parity, alcohol consumption, menopausal hormone therapy, and number of first‐degree relatives (FDR) with BC. Factors such as light at night (Wang *et al.*, 2016), physical activity (Park *et al.*, 2013), and sleep quality (Wang *et al.*, 2016) were rarely included. The predictive accuracy of the ERS was limited with AUCs ranging from 0.517 to 0.594 in our cohort. The score of Dierssen–Sotos (Dierssen‐Sotos *et al.*, 2018) which was derived from a case–control study in a Spanish population achieved a relatively good performance and was used for subsequent analyses. For the score of Wang *et al. *(2016) who had provided separate algorithms for premenopausal women and postmenopausal women, the predictive performance was estimated by the algorithm for postmenopausal women only since most of the women in our study population were postmenopausal.

**Table 2:** Predictors included in previous studies and performance of corresponding ERS in the ESTHER study. No., number.

Predictors	Previously proposed ERSs							Variables available in ESTHER
	Gail *et al. *(1989)	Novotny *et al. *(2006)	Park *et al. *(2013)	Wang *et al. *(2016)	Maas *et al. *(2016)	Dierssen‐Sotos *et al. *(2018)	Rudolph *et al. *(2018)	
Age	✓	✓	✓	✓	✓	✓	✓	✓
Age at menarche	✓	✓	✓	✓	✓	✓	✓	✓
Age at first live birth	✓	✓		✓	✓	✓	✓	✓
Parity		✓	✓	✓	✓	✓	✓	✓
Breast feeding				✓				✓
Menopausal status			✓		✓	✓		✓
Age at menopause			✓		✓	✓		✓
Ever use of oral contraceptive			✓	✓				✓
Current use of MHT				✓	✓	✓	✓	✓
BMI		✓	✓	✓	✓	✓	✓	✓
Height					✓	✓	✓	✓
Alcohol consumption				✓	✓	✓	✓	✓
Smoking					✓	✓		✓
Physical activity			✓					✓
Sleep quality				✓				✓
Light at night				✓				
No. of FDRs with cancer history		✓						✓
Family history of BC			✓		✓	✓		✓
No. of FDRs with BC	✓	✓	✔	✓				✓
Breast inflammation		✓						
Number of previous biopsies	✓	✓		✓				
AUC in ESTHER	0.540 (0.520–0.559)	0.552 (0.533–0.570)	0.517 (0.498–0.536)	0.519^a^ (0.502–0.536)	0.556 (0.538–0.574)	0.594 (0.578–0.612)	0.560 (0.539–0.578)	

a AUC was calculated for postmenopausal women.

Figure S1 shows the pairwise correlations between the three types of risk scores. Very low, insignificant positive correlations were observed for each pair of scores.

Individual associations for MRS, GRS, and ERS (Dierssen–Sotos Score) with BC risk are summarized in Table 3. For GRS and ERS, having a score in the top quartile was associated with a significantly increased risk of BC compared to the lowest quartile without adjustment. However, none of the current MRS presented a significant association with BC risk. These results did not materially change after adjusting for the complementary risk scores and leukocyte composition. For example, after adjusting for MRS, ERS, and leukocyte composition, women in the highest GRS quartile had a 2.21‐fold increased risk of BC compared with women in the lowest quartile. Adjusted ORs (95% CI) per standard deviation increase were 1.07 (0.84–1.36), 1.03 (0.81–1.31), 1.01 (0.79–1.30), 1.48 (1.16–1.90), and 1.40 (1.09–1.80) for MRS (423‐CpGs), MRS (248‐CpGs), MRS (131‐CpGs), GRS, and ERS, respectively.

**Table 3:** Association of the risk scores with BC incidence in the ESTHER study.

Risk scores	Quartiles of risk score	Cases	Controls	Model I^a^ OR (95% CI)	Model II^b^ OR (95% CI)
MRS (423‐CpGs)	IQR	104 (90, 118)	98 (85, 115)		
	Q1 (≤ 85)	16	66	Ref.	Ref.
	Q2 (85–98]	26	66	1.60 (0.77–3.29)	1.49 (0.71–3.11)
	Q3 (98–115]	29	66	1.86 (0.91–3.81)	1.93 (0.93–4.01)
	Q4 > 115	30	65	1.82 (0.89–3.75)	1.67 (0.79–3.50)
	OR per SD increase			1.10 (0.87–1.39)	1.07 (0.84–1.36)
MRS (248‐CpGs)	IQR	53 (36, 67)	46 (35, 69)		
	Q1 (≤ 35)	23	70	Ref.	Ref.
	Q2 (35–46]	22	62	1.03 (0.51–2.09)	1.08 (0.53–2.23)
	Q3 (46–69]	26	68	1.20 (0.61–2.35)	1.27 (0.64–2.54)
	Q4 > 69	30	63	1.33 (0.69–2.59)	1.27 (0.64–2.50)
	OR per SD increase			1.06 (0.84–1.40)	1.03 (0.81–1.31)
MRS (131‐CpGs)	IQR	26 (16, 45)	26 (15, 45)		
	Q1 (≤ 15)	24	68	Ref.	Ref.
	Q2 (15–26]	24	65	0.94 (0.44–2.00)	0.82 (0.37–1.80)
	Q3 (26–45]	25	62	1.22 (0.58–2.53)	1.34 (0.63–2.85)
	Q4 > 45	28	68	1.16 (0.59–2.31)	1.30 (0.64–2.63)
	OR per SD increase			1.02 (0.80–1.32)	1.01 (0.79–1.30)
GRS	IQR	15.50 (15.06, 15.88)	15.21 (14.83, 15.59)		
	Q1 (≤ 14.83)	20	66	Ref.	Ref.
	Q2 (14.83–15.21]	17	66	0.85 (0.41–1.77)	0.80 (0.37–1.72)
	Q3 (15.21–15.59]	23	66	1.15 (0.58–2.29)	1.18 (0.58–2.42)
	Q4 > 15.59	41	65	2.08 (1.10–3.93)	2.21 (1.14–4.30)
	OR per SD increase			1.50 (1.18–1.91)	1.48 (1.16–1.90)
ERS (Dierssen‐Sotos)	IQR	0.99 (0.39, 1.51)	0.69 (0.19, 1.42)		
	Q1 (≤ 0.19)	13	60	Ref.	Ref.
	Q2 (0.19–0.69]	20	61	1.47 (0.70–3.07)	1.46 (0.67–3.14)
	Q3 (0.69–1.42]	28	61	2.07 (1.02–4.18)	2.06 (0.99–4.27)
	Q4 > 1.42	30	60	2.23 (1.11–4.50)	2.50 (1.19–5.25)
	OR per SD increase			1.36 (1.08–1.71)	1.40 (1.09–1.80)

a Model I includes individual risk score and, in case of MRS, leukocyte composition.

b Model II includes individual MRS (423‐CpGs or 248‐CpGs or 131‐CpGs), GRS, ERS, and leukocyte composition.

The predictive performances of individual risk scores and score combinations are presented in Table 4. The predictive accuracy of individual risk score varied with AUCs ranging from 0.517 to 0.612. The GRS was more predictive of BC risk [AUC = 0.612, (95% CI: 0.590–0.632)] than the MRS and ERS. The three individual MRSs, especially the MRS of 131‐CpGs, exhibited very limited accuracy in predicting BC incidence when comparing to GRS or ERS. Moreover, combining 423‐CpGs (the best performing MRS) with GRS or ERS improved the predictive performance to a very limited extent [AUC_423‐CpGs + GRS_ = 0.621 (0.593–0.640) vs. AUC_GRS_ = 0.612, (0.590–0.632) and AUC_423‐CpGs + ERS_ = 0.603 (0.583–0.621) vs. AUC_ERS_ = 0.594 (0.578–0.612)]. Of note, a full risk model combing MRS, GRS, and ERS outperformed all other risk prediction models [AUC = 0.637 (0.616–0.657)]. Further tests for goodness of fit and tailed‐based tests for the combined model were not statistically significant at *P* < 0.05 (results not shown). Additionally, consistent performance of either individual (MRS, GRS, ERS) or combined risk score (MRS + GRS + ERS) was observed in the time‐to‐diagnosis‐specific analyses (Table S4).

**Table 4:** Risk prediction by individual and combined risk scores for BC.

Risk scores	AUC (95% CI)	Combined AUC (95% CI)
Single risk scores		
423‐CpGs	0.557 (0.536–0.580)	
248‐CpGs	0.545 (0.524–0.563)	
131‐CpGs	0.517 (0.501–0.542)	
GRS	0.612 (0.590–0.632)	
ERS	0.594 (0.578–0.612)	
Multiple risk scores		
423‐CpGs + GRS		0.621 (0.593–0.640)
423‐CpGs + ERS		0.603 (0.583–0.621)
GRS + ERS		0.635 (0.615–0.656)
423‐CpGs + GRS + ERS		0.637 (0.616–0.657)

## 4 Discussion

To our knowledge, this is the first study evaluating and comparing the predictive performance of whole‐blood DNA methylation, and genetic and ERSs for BC incidence in a prospective cohort with up to 14 years of follow‐up. All three types of risk scores were predictive of BC risk. A GRS based on multiple common variants (269 SNPs) predicted BC incidence with much higher accuracy than a MRS based on previously identified CpGs and an ERS derived from previous studies. The combination of MRS, GRS, and ERS enhanced the risk prediction with an AUC of approximately 0.64. Similar predictive accuracies of either individual or combined risk scores were observed in specific subgroups defined by time to diagnosis.

Previous studies have demonstrated the diagnostic efficiency of aberrant DNA methylation for BC (Guan *et al.*, 2018). In the current study, we constructed MRSs based on previously identified CpGs and evaluated its predictive performance for BC incidence. Although the discriminatory power of the MRS was limited, the poor performance should not be misinterpreted as implying that the methylation status of those CpGs does not play a role in BC development. A potential explanation for low predictive value could be differences between the study populations of the previous studies in which the CpGs were identified and our study population. For example, the majority of the 248‐CpGs (227 out of 248 CpGs) and all of the 131‐CpGs were both identified within a prospective cohort of women who were BC‐free themselves at recruitment, but had a biological sister with BC (Xu *et al.*, 2013; Xu *et al.*, 2019). So, the association of methylation at these CpGs with BC may not be generalizable to the women from the general population (Xu *et al.*, 2019). Interestingly, the MRS based on 423 CpGs, which was derived from genetically predicted rather than directly measured CpG methylation levels, performed even better in our study than the MRS of the other two sets that had been derived from measured methylation levels. It is also worth noting that there was hardly any overlap between the genetically predicted CpGs and methylation measured CpGs (423‐CpGs and 248‐CpGs, 423‐CpGs and 131‐CpGs) which supports suggestions that there may still be much room for the improvement in the derivation of MRS.

The performance of our version of GRS score is generally consistent with the observation from a recent GWAS used to establish a GRS for risk stratification and early detection of BC (Mavaddat *et al.*, 2019). In the current analysis, the performance of our version of GRS (269 SNPs) with an OR (95% CI, per SD increase) of 1.48 (1.16–1.90) and an AUC (95% CI) of 0.61 (0.59–0.63) was similar albeit slightly less favorable compared with performance previously reported for the original GRS containing 313 SNPs [OR = 1.61, (1.57–1.65), AUC = 0.63 (0.63–0.65)]. A potential reason for the slightly weaker performance in our study could be incomplete validation, as 29 out of 313 SNPs were excluded because of missing values in > 10% of our sample.

We also conducted a literature search to identify ERSs used for BC risk prediction and validated their predictive performance for BC incidence in our study sample. Although many researchers have put substantial efforts in developing models for risk prediction, the overall results are not promising (Anothaisintawee *et al.*, 2012). In the current study, most of the ERSs yielded poor predictive accuracies with AUCs ranging from 0.52 to 0.59. The lack of predictive value of ERSs might be explained by heterogeneity of study populations used to derive the scores and missing information on some risk factors (i.e., numbers of previous breast biopsies, breast inflammation, and light at night) in several models (Gail *et al.*, 1989; Novotny *et al.*, 2006; Wang *et al.*, 2016). However, the main reason for the low predictive values would probably be that the risk factors included in these scores were not good predictors of BC risk. Notably, improved predictive accuracy was observed when ERS was combined with GRS, suggesting that rather than developing more risk scores based on environmental risk factors, future studies should explore possibilities of enhancing predictive performance by combining risk factors with novel laboratory markers, such as MRSs or GRSs.

We performed time‐to‐diagnosis‐specific analyses to explore whether the individual risk scores or score combinations performed better in cases who had shorter time to diagnosis. In a subgroup analysis, only the performance of ERS (Dierssen–Sotos) and a combination of three risk scores supported the hypothesis, but differences between shorter and longer time to diagnosis were small and did not reach statistical significance. However, power for such subgroup analyses was very limited due to sample size limitations. The predictive values of the three individual risk scores or score combinations for subgroup (e.g., defined by age and follow‐up time) or subtype‐specific BC screening warrant further exploration.

Compared with GRS, MRS was less predictive of BC risk. This, however, does not imply that MRS does not hold potential for risk stratification for BC risk. Whereas large‐scale GWASs have been conducted since more than 10 years (Easton *et al.*, 2007), large‐scale epigenome‐wide association studies have been initiated only recently (Xu *et al.*, 2013) and may yield substantially better MRS in the future. In contrast to GRS, MRS and ERS are though not static over lifetime. Although this may be considered a disadvantage for straightforward risk stratification (e.g., determining a starting age of screening), MRS and ERS may have additional use in reflecting the merits of specific prevention efforts.

Specific strengths of our study include its longitudinal design in which blood samples for methylation and genotyping analysis, along with environmental risk factor data, were collected years before BC diagnosis. This allowed for simultaneous evaluation and comparison of the ability of three different types of risk variables for BC risk prediction. In addition, the selection of incident BC cases through linkage to the Saarland Cancer Registry ensured an almost complete ascertainment of incident cancer cases in the population from which our study participants originated. However, our study has several limitations that require careful discussion. First, the limited sample size restricted the study’s power and precision of estimates, particularly in stratified analysis. For example, the AUC for score combination in cases with time to diagnosis ≤ 7 years was larger than that in cases with time to diagnosis > 7 years, but this difference did not reach statistical significance. Future studies with larger sample sizes should address predictive values of MRS, GRS, and ERS and their combination for BC risk prediction in specific population groups. Likewise, future studies with larger sample size should also address potential improvement of prediction by including interactions between the included factors such as gene–environment interactions (Barrdahl *et al.*, 2014; Campa *et al.*, 2011; Maas *et al.*, 2016; Nickels *et al.*, 2013; Rudolph *et al.*, 2015; Schoeps *et al.*, 2014). Second, it is difficult to compare the results between our study and previous studies, where different methylation analysis techniques were used to investigate different CpG sites. Although the EPIC assay covers more CpGs across the whole genome compared with the Illumina 27 or 450K used in previous studies, it cannot be ruled out that some key loci with powerful diagnostic performance were missed. Finally, although we paid careful attention to internal validation, further validation in external, independent cohorts would be highly desirable. In particular, our results pertain to a Caucasian study population from Germany; hence, the performance of our versions of MRS, GRS, and ERS needs to be validated and potentially adapted for ethnically diverse populations.

## 5 Conclusion

In summary, despite these limitations, our study provides new detailed insights into the individual and joint associations of MRS, GRS, and ERS with BC risk. Although the contribution of all three types of risk scores to risk stratification is still modest for the time being, with GRS so far slightly outperforming MRS and ERS, our findings demonstrated that combing MRS, GRS, and ERS can enable more precise risk prediction and therefore holds potential to improve risk stratification in BC screening. With further improvements of GRS and MRS by large‐scale international GWAS and EWAS efforts, and incorporation of additional risk signatures, such as microRNA‐based signatures (Hamam *et al.*, 2017), substantial further improvement of risk stratification should become possible which will enable more targeted, risk‐adopted approaches in BC screening.

## Conflict of interest

The authors declare no conflict of interest

## Author contributions

HB and YZ conceived the study. ZG analyzed the data and drafted the manuscript under the supervision of HB and YZ. KW participated in statistical analysis and interpretation of genetic data. JR participated in manuscript revision. BH conducted data collection from the Saarland Cancer Registry. HB, BS, and YZ supervised the data collection of the ESTHER study. HB initiated and led the ESTHER study and contributed to all aspects of this work. All authors made significant contributions to the manuscript, and all authors read and approved the final version.

## Ethics declaration and consent to participate

The study was conducted in adherence with the Code of Ethics of the World Medical Association (Declaration of Helsinki) for experiments involving humans, and informed consent was collected from all participants. The study was approved by the ethics committees of the University of Heidelberg and of the state medical board of Saarland, Germany.

## Consent for publication

The consent of this manuscript has not been previously published and is not under consideration for publication elsewhere.

## Authors’ information

All authors’ information has been presented in the title page.

## Supporting information


**Table S1** **.** Individual association of the previously identified CpGs with BC risk in the ESTHER study and reported methylation direction.
**Table S2** **.** SNPs used for genetic risk score construction.
**Table S3** **.** Environmental risk scores from previous studies and availability/modification of related variables in ESTHER.
**Table S4** **.** Performance of risk scores with respect to time to diagnosis in cases.
**Fig. S1.** Correlation among risk scores.

## Data Availability

DNA methylation, and genetic and demographic data of this study are available from the corresponding author upon reasonable request

## References

[mol212594-bib-0001] Anderson CA , Pettersson FH , Clarke GM , Cardon LR , Morris AP and Zondervan KT (2010) Data quality control in genetic case‐control association studies. Nat Protoc 5, 1564–1573. 21085122 10.1038/nprot.2010.116 PMC3025522

[mol212594-bib-0002] Anothaisintawee T , Teerawattananon Y , Wiratkapun C , Kasamesup V and Thakkinstian A (2012) Risk prediction models of breast cancer: a systematic review of model performances. Breast Cancer Res Treat 133, 1–10. 22076477 10.1007/s10549-011-1853-z

[mol212594-bib-0003] Barrdahl M , Canzian F , Joshi AD , Travis RC , Chang‐Claude J , Auer PL , Gapstur SM , Gaudet M , Diver WR , Henderson BE *et al* (2014) Post‐GWAS gene‐environment interplay in breast cancer: results from the Breast and Prostate Cancer Cohort Consortium and a meta‐analysis on 79,000 women. Hum Mol Genet 23, 5260–5270. 24895409 10.1093/hmg/ddu223 PMC4159150

[mol212594-bib-0004] Bray F , Ferlay J , Soerjomataram I , Siegel RL , Torre LA and Jemal A (2018) Global cancer statistics 2018: GLOBOCAN estimates of incidence and mortality worldwide for 36 cancers in 185 countries. CA Cancer J Clin 68, 394–424. 30207593 10.3322/caac.21492

[mol212594-bib-0005] Burton H , Chowdhury S , Dent T , Hall A , Pashayan N and Pharoah P (2013) Public health implications from COGS and potential for risk stratification and screening. Nat Genet 45, 349–351. 23535723 10.1038/ng.2582

[mol212594-bib-0006] Campa D , Kaaks R , Le Marchand L , Haiman CA , Travis RC , Berg CD , Buring JE , Chanock SJ , Diver WR , Dostal L *et al* (2011) Interactions between genetic variants and breast cancer risk factors in the breast and prostate cancer cohort consortium. J Natl Cancer Inst 103, 1252–1263. 21791674 10.1093/jnci/djr265 PMC3156803

[mol212594-bib-0007] Chang CC , Chow CC , Tellier LC , Vattikuti S , Purcell SM and Lee JJ (2015) Second‐generation PLINK: rising to the challenge of larger and richer datasets. GigaScience 4, 7. 25722852 10.1186/s13742-015-0047-8 PMC4342193

[mol212594-bib-0008] Dierssen‐Sotos T , Gomez‐Acebo I , Palazuelos C , Fernandez‐Navarro P , Altzibar JM , Gonzalez‐Donquiles C , Ardanaz E , Bustamante M , Alonso‐Molero J , Vidal C *et al* (2018) Validating a breast cancer score in Spanish women. The MCC‐Spain study. Sci Rep 8, 3036. 29445177 10.1038/s41598-018-20832-0 PMC5813036

[mol212594-bib-0009] Du P , Zhang X , Huang CC , Jafari N , Kibbe WA , Hou L and Lin SM (2010) Comparison of Beta-value and M-value methods for quantifying methylation levels by microarray analysis. BMC bioinformatics 11, 587–595. 21118553 10.1186/1471-2105-11-587 PMC3012676

[mol212594-bib-0010] Easton DF , Pooley KA , Dunning AM , Pharoah PD , Thompson D , Ballinger DG , Struewing JP , Morrison J , Field H , Luben R *et al* (2007) Genome‐wide association study identifies novel breast cancer susceptibility loci. Nature 447, 1087–1093. 17529967 10.1038/nature05887 PMC2714974

[mol212594-bib-0011] Gail MH , Brinton LA , Byar DP , Corle DK , Green SB , Schairer C and Mulvihill JJ (1989) Projecting individualized probabilities of developing breast cancer for white females who are being examined annually. J Natl Cancer Inst 81, 1879–1886. 2593165 10.1093/jnci/81.24.1879

[mol212594-bib-0012] Guan Z , Yu H , Cuk K , Zhang Y and Brenner H (2018). Whole‐blood DNA methylation markers in early detection of breast cancer: a systematic literature review. Cancer Epidemiol Biomarkers Prev 28, 496–505. 30487132 10.1158/1055-9965.EPI-18-0378

[mol212594-bib-0013] Hamam R , Hamam D , Alsaleh KA , Kassem M , Zaher W , Alfayez M , Aldahmash A and Alajez NM (2017) Circulating microRNAs in breast cancer: novel diagnostic and prognostic biomarkers. Cell Death Dis 8, e3045. 28880270 10.1038/cddis.2017.440 PMC5636984

[mol212594-bib-0014] Houseman EA , Accomando WP , Koestler DC , Christensen BC , Marsit CJ , Nelson HH , Wiencke JK and Kelsey KT (2012) DNA methylation arrays as surrogate measures of cell mixture distribution. BMC Bioinformatics 13, 86. 22568884 10.1186/1471-2105-13-86 PMC3532182

[mol212594-bib-0015] Independent UK Panel on Breast Cancer Screening (2012) The benefits and harms of breast cancer screening: an independent review. Lancet 380, 1778–1786. 23117178 10.1016/S0140-6736(12)61611-0

[mol212594-bib-0016] Joo JE , Dowty JG , Milne RL , Wong EM , Dugue PA , English D , Hopper JL , Goldgar DE , Giles GG , Southey MC *et al* (2018) Heritable DNA methylation marks associated with susceptibility to breast cancer. Nat Commun 9, 867–878. 29491469 10.1038/s41467-018-03058-6 PMC5830448

[mol212594-bib-0017] Lanfear DE , Gibbs JJ , Li J , She R , Petucci C , Culver JA , Tang WHW , Pinto YM , Williams LK , Sabbah HN *et al* (2017) Targeted metabolomic profiling of plasma and survival in heart failure patients. JACC Heart Fail 5, 823–832. 29096792 10.1016/j.jchf.2017.07.009 PMC5679305

[mol212594-bib-0018] Maas P , Barrdahl M , Joshi AD , Auer PL , Gaudet MM , Milne RL , Schumacher FR , Anderson WF , Check D , Chattopadhyay S *et al* (2016) Breast cancer risk from modifiable and nonmodifiable risk factors among white women in the United States. JAMA Oncol 2, 1295–1302. 27228256 10.1001/jamaoncol.2016.1025 PMC5719876

[mol212594-bib-0019] Madigan MP , Ziegler RG , Benichou J , Byrne C and Hoover RN (1995) Proportion of breast cancer cases in the United States explained by well‐established risk factors. J Natl Cancer Inst 87, 1681–1685. 7473816 10.1093/jnci/87.22.1681

[mol212594-bib-0020] Mavaddat N , Michailidou K , Dennis J , Lush M , Fachal L , Lee A , Tyrer JP , Chen TH , Wang Q , Bolla MK *et al* (2019) Polygenic risk scores for prediction of breast cancer and breast cancer subtypes. Am J Hum Genet 104, 21–34. 30554720 10.1016/j.ajhg.2018.11.002 PMC6323553

[mol212594-bib-0021] Mavaddat N , Pharoah PD , Michailidou K , Tyrer J , Brook MN , Bolla MK , Wang Q , Dennis J , Dunning AM , Shah M *et al* (2015) Prediction of breast cancer risk based on profiling with common genetic variants. J Natl Cancer Inst 107, djv036 10.1093/jnci/djv036 25855707 PMC4754625

[mol212594-bib-0022] Michailidou K , Beesley J , Lindstrom S , Canisius S , Dennis J , Lush MJ , Maranian MJ , Bolla MK , Wang Q , Shah M *et al* (2015) Genome‐wide association analysis of more than 120,000 individuals identifies 15 new susceptibility loci for breast cancer. Nat Genet 47, 373–380. 25751625 10.1038/ng.3242 PMC4549775

[mol212594-bib-0023] Michailidou K , Lindstrom S , Dennis J , Beesley J , Hui S , Kar S , Lemacon A , Soucy P , Glubb D , Rostamianfar A *et al* (2017) Association analysis identifies 65 new breast cancer risk loci. Nature 551, 92–94. 29059683 10.1038/nature24284 PMC5798588

[mol212594-bib-0024] Miller SA , Dykes DD and Polesky HF (1988) A simple salting out procedure for extracting DNA from human nucleated cells. Nucleic Acids Res 16, 1215. 3344216 10.1093/nar/16.3.1215 PMC334765

[mol212594-bib-0025] Nickels S , Truong T , Hein R , Stevens K , Buck K , Behrens S , Eilber U , Schmidt M , Haberle L , Vrieling A *et al* (2013) Evidence of gene‐environment interactions between common breast cancer susceptibility loci and established environmental risk factors. PLoS Genet 9, e1003284. 23544014 10.1371/journal.pgen.1003284 PMC3609648

[mol212594-bib-0026] Novotny J , Pecen L , Petruzelka L , Svobodnik A , Dusek L , Danes J and Skovajsova M (2006) Breast cancer risk assessment in the Czech female population–an adjustment of the original Gail model. Breast Cancer Res Treat 95, 29–35. 16319995 10.1007/s10549-005-9027-5

[mol212594-bib-0027] Pace LE and Keating NL (2014) A systematic assessment of benefits and risks to guide breast cancer screening decisions. JAMA 311, 1327–1335. 24691608 10.1001/jama.2014.1398

[mol212594-bib-0028] Park B , Ma SH , Shin A , Chang MC , Choi JY , Kim S , Han W , Noh DY , Ahn SH , Kang D *et al* (2013) Korean risk assessment model for breast cancer risk prediction. PLoS ONE 8, e76736. 24204664 10.1371/journal.pone.0076736 PMC3808381

[mol212594-bib-0029] Peto R (2011) The fraction of cancer attributable to lifestyle and environmental factors in the UK in 2010. Br J Cancer 105(Suppl 2), S1. 10.1038/bjc.2011.473 PMC3252054 22158311

[mol212594-bib-0030] Rudolph A , Milne RL , Truong T , Knight JA , Seibold P , Flesch‐Janys D , Behrens S , Eilber U , Bolla MK , Wang Q *et al* (2015) Investigation of gene‐environment interactions between 47 newly identified breast cancer susceptibility loci and environmental risk factors. Int J Cancer 136, E685–E696. 25227710 10.1002/ijc.29188 PMC4289418

[mol212594-bib-0031] Rudolph A , Song M , Brook MN , Milne RL , Mavaddat N , Michailidou K , Bolla MK , Wang Q , Dennis J , Wilcox AN *et al* (2018) Joint associations of a polygenic risk score and environmental risk factors for breast cancer in the Breast Cancer Association Consortium. Int J Epidemiol 47, 526–536. 29315403 10.1093/ije/dyx242 PMC5913605

[mol212594-bib-0032] Schoeps A , Rudolph A , Seibold P , Dunning AM , Milne RL , Bojesen SE , Swerdlow A , Andrulis I , Brenner H , Behrens S *et al* (2014) Identification of new genetic susceptibility loci for breast cancer through consideration of gene‐environment interactions. Genet Epidemiol 38, 84–93. 24248812 10.1002/gepi.21771 PMC3995140

[mol212594-bib-0033] Schottker B , Haug U , Schomburg L , Kohrle J , Perna L , Muller H , Holleczek B and Brenner H (2013) Strong associations of 25‐hydroxyvitamin D concentrations with all‐cause, cardiovascular, cancer, and respiratory disease mortality in a large cohort study. Am J Clin Nutr 97, 782–793. 23446902 10.3945/ajcn.112.047712

[mol212594-bib-0034] Song M , Kraft P , Joshi AD , Barrdahl M and Chatterjee N (2015) Testing calibration of risk models at extremes of disease risk. Biostatistics 16, 143–154. 25027274 10.1093/biostatistics/kxu034 PMC4263225

[mol212594-bib-0035] van Veldhoven K , Polidoro S , Baglietto L , Severi G , Sacerdote C , Panico S , Mattiello A , Palli D , Masala G , Krogh V *et al* (2015) Epigenome‐wide association study reveals decreased average methylation levels years before breast cancer diagnosis. Clin Epigenetics, 7, 67. 26244061 10.1186/s13148-015-0104-2 PMC4524428

[mol212594-bib-0036] Wang F , Dai J , Li M , Chan WC , Kwok CC , Leung SL , Wu C , Li W , Yu WC , Tsang KH *et al* (2016) Risk assessment model for invasive breast cancer in Hong Kong women. Medicine 95, e4515. 27512870 10.1097/MD.0000000000004515 PMC4985325

[mol212594-bib-0037] Winters S , Martin C , Murphy D and Shokar NK (2017) Breast cancer epidemiology, prevention, and screening. Prog Mol Biol Transl Sci 151, 1–32. 29096890 10.1016/bs.pmbts.2017.07.002

[mol212594-bib-0038] Xie C , Leung YK , Chen A , Long DX , Hoyo C and Ho SM (2019) Differential methylation values in differential methylation analysis. Bioinformatics 35, 1094–1097. 30184051 10.1093/bioinformatics/bty778 PMC6449748

[mol212594-bib-0039] Xu ZL , Bolick SCE , DeRoo LA , Weinberg CR , Sandler DP and Taylor JA (2013) Epigenome‐wide association study of breast cancer using prospectively collected sister study samples. J Natl Cancer Inst 105, 694–700. 23578854 10.1093/jnci/djt045 PMC3653821

[mol212594-bib-0040] Xu Z , Sandler DP and Taylor JA (2019) Blood DNA methylation and breast cancer: a prospective case‐cohort analysis in the Sister Study. J Natl Cancer Inst. djz065 10.1093/jnci/djz065 [Epub ahead of print] 30989176 PMC7489106

[mol212594-bib-0041] Yang Y , Wu L , Shu XO , Cai Q , Shu X , Li B , Guo X , Ye F , Michailidou K , Bolla MK *et al* (2019) Genetically predicted levels of DNA methylation biomarkers and breast cancer risk: data from 228,951 women of European descent. J Natl Cancer Inst. djz109 10.1093/jnci/djz109 31143935 PMC7073907

[mol212594-bib-0042] Zaimi I , Pei D , Koestler DC , Marsit CJ , De Vivo I , Tworoger SS , Shields AE , Kelsey KT and Michaud DS (2018) Variation in DNA methylation of human blood over a 1‐year period using the Illumina MethylationEPIC array. Epigenetics 13, 1056–1071. 30270718 10.1080/15592294.2018.1530008 PMC6342169

[mol212594-bib-0043] Zhang Y , Wilson R , Heiss J , Breitling LP , Saum KU , Schottker B , Holleczek B , Waldenberger M , Peters A and Brenner H (2017) DNA methylation signatures in peripheral blood strongly predict all‐cause mortality. Nat Commun 8, 14617. 28303888 10.1038/ncomms14617 PMC5357865

