# Supplementary material for: Individual and joint performance of DNA methylation profiles, genetic risk score and environmental risk scores for predicting breast cancer risk
Source: Mol Oncol. 2019 Nov 19;14(1):42–53. doi: 10.1002/1878-0261.12594 (PMC6944111; doi:10.1002/1878-0261.12594)

Supplementary Table 1. Individual association of the previously identified CpGs with BC risk in the ESTHER study and reported methylation direction

| CpG sets | Probe | CHR | Gene | Region | OR^a^ | 95%CI | *P*-value | *Q*-value^b^ | Previously reported direction |
| --- | --- | --- | --- | --- | --- | --- | --- | --- | --- |
| 423-CpGs  (Yang et al., 2019) |  |  |  |  |  |  |  |  |  |
|  | cg00025211 | 1 | *NUDT17* | Body | 0.96 | 0.62-1.47 | 0.8361 |  | Hyper-methylation |
|  | cg01391297 | 1 | *LOC645676* | TSS1500 | 1.11 | 0.78-1.58 | 0.5604 |  | Hyper-methylation |
|  | cg04794690 | 1 |  |  | 1.48 | 0.85-2.57 | 0.1616 |  | Hyper-methylation |
|  | cg05925577 | 1 |  |  | 0.84 | 0.6-1.19 | 0.325 |  | Hyper-methylation |
|  | cg07189204 | 1 | *SCAMP3* | 3'UTR | 1.15 | 0.72-1.84 | 0.5657 |  | Hyper-methylation |
|  | cg08005692 | 1 | *ASH1L* | 5'UTR | 0.91 | 0.68-1.22 | 0.5411 |  | Hyper-methylation |
|  | cg08614201 | 1 | *CD160* | 5'UTR | 0.76 | 0.5-1.15 | 0.1943 |  | Hyper-methylation |
|  | cg09578353 | 1 | *APITD1* | 5'UTR | 1.02 | 0.79-1.31 | 0.8887 |  | Hyper-methylation |
|  | cg10207631 | 1 |  |  | 1.06 | 0.72-1.55 | 0.771 |  | Hyper-methylation |
|  | cg10802414 | 1 | *HCN3* | Body | 1.02 | 0.79-1.32 | 0.8788 |  | Hyper-methylation |
|  | cg12688265 | 1 | *GBAP1* | TSS1500 | 0.80 | 0.58-1.12 | 0.1919 |  | Hyper-methylation |
|  | cg12832565 | 1 | *CD160* | TSS200 | 0.92 | 0.6-1.43 | 0.7175 |  | Hyper-methylation |
|  | cg14528056 | 1 | *GBAP1* | Body | 1.15 | 0.79-1.67 | 0.4614 |  | Hyper-methylation |
|  | cg15554126 | 1 | *PEX14* | 3'UTR | 1.28 | 0.89-1.85 | 0.1862 |  | Hyper-methylation |
|  | cg16928044 | 1 | *NUDT17* | TSS1500 | 1.05 | 0.7-1.56 | 0.8275 |  | Hyper-methylation |
|  | cg18568145 | 1 | *FAM189B* | TSS1500 | 1.16 | 0.76-1.76 | 0.4943 |  | Hyper-methylation |
|  | cg20311333 | 1 | *GBAP1* | TSS1500 | 1.03 | 0.75-1.41 | 0.8407 |  | Hyper-methylation |
|  | **cg20975414** | 1 | *CD160* | TSS200 | 1.59 | 1.04-2.42 | 0.0313 | 0.6677 | Hyper-methylation |
|  | cg22221025 | 1 |  |  | 0.95 | 0.7-1.3 | 0.7559 |  | Hyper-methylation |
|  | cg22515654 | 1 | *PEX14* | Body | 1.30 | 0.92-1.85 | 0.137 |  | Hyper-methylation |
|  | cg22715764 | 1 | *PEX14* | Body | 0.72 | 0.52-1 | 0.0522 |  | Hyper-methylation |
|  | cg23975251 | 1 | *PEX14* | Body | 1.10 | 0.72-1.68 | 0.6568 |  | Hyper-methylation |
|  | **cg24512897** | 1 |  |  | 1.57 | 1.07-2.3 | 0.0201 | 0.6677 | Hyper-methylation |
|  | cg25614726 | 1 | *APITD1* | TSS200 | 0.98 | 0.73-1.32 | 0.8952 |  | Hyper-methylation |
|  | cg26668989 | 1 |  |  | 1.02 | 0.74-1.4 | 0.9208 |  | Hyper-methylation |
|  | cg01926269 | 2 | *POMC* | TSS200 | 1.07 | 0.76-1.52 | 0.6874 |  | Hyper-methylation |
|  | cg04411307 | 2 | *ANTXR1* | Body | 1.00 | 0.74-1.34 | 0.9906 |  | Hyper-methylation |
|  | cg08030082 | 2 | *POMC* | TSS1500 | 1.13 | 0.75-1.68 | 0.5653 |  | Hyper-methylation |
|  | cg09505516 | 2 | *ADCY3* | Body | 1.04 | 0.79-1.36 | 0.8045 |  | Hyper-methylation |
|  | **cg09859492** | 2 | *TNS1* | Body | 1.85 | 1.23-2.78 | 0.0031 | 0.6677 | Hyper-methylation |
|  | cg09916783 | 2 | *POMC* | TSS1500 | 1.06 | 0.74-1.52 | 0.7405 |  | Hyper-methylation |
|  | cg11023668 | 2 | *ADCY3* | Body | 1.18 | 0.9-1.55 | 0.2416 |  | Hyper-methylation |
|  | cg11557618 | 2 |  |  | 0.81 | 0.6-1.09 | 0.1665 |  | Hyper-methylation |
|  | cg16302441 | 2 | *POMC* | TSS1500 | 1.20 | 0.85-1.7 | 0.2943 |  | Hyper-methylation |
|  | cg16773799 | 2 | *GRHL1* | Body | 1.42 | 1-2.02 | 0.0508 |  | Hyper-methylation |
|  | cg18011760 | 2 |  |  | 1.17 | 0.89-1.53 | 0.269 |  | Hyper-methylation |
|  | cg18309255 | 2 | *DIRC3* | Body | 0.96 | 0.74-1.23 | 0.7315 |  | Hyper-methylation |
|  | cg18789177 | 2 |  |  | 0.93 | 0.65-1.32 | 0.6838 |  | Hyper-methylation |
|  | cg20387815 | 2 | *POMC* | TSS200 | 0.89 | 0.65-1.22 | 0.465 |  | Hyper-methylation |
|  | cg22900229 | 2 | *POMC* | TSS1500 | 1.07 | 0.76-1.5 | 0.6996 |  | Hyper-methylation |
|  | cg22900681 | 2 | *EFR3B* | Body | 0.93 | 0.66-1.3 | 0.6592 |  | Hyper-methylation |
|  | cg24425171 | 2 | *POMC* | TSS200 | 1.02 | 0.7-1.48 | 0.9157 |  | Hyper-methylation |
|  | cg03895047 | 3 |  |  | 1.04 | 0.77-1.4 | 0.8005 |  | Hyper-methylation |

Supplementary Table 1. Continued

| CpG sets | Probe | CHR | Gene | Region | OR^a^ | 95%CI | *P*-value | *Q*-value^b^ | Previously reported direction |
| --- | --- | --- | --- | --- | --- | --- | --- | --- | --- |
|  | cg12970662 | 3 | *PRSS42* | Body | 1.06 | 0.74-1.54 | 0.7414 |  | Hyper-methylation |
|  | cg20202413 | 3 | *PTH1R* | 5'UTR | 1.10 | 0.77-1.57 | 0.5926 |  | Hyper-methylation |
|  | cg27384355 | 3 | *C3orf26* | Body | 1.11 | 0.86-1.43 | 0.438 |  | Hyper-methylation |
|  | cg06935464 | 4 | *TLR10* | TSS200 | 0.82 | 0.58-1.15 | 0.2482 |  | Hyper-methylation |
|  | cg16030869 | 4 |  |  | 1.12 | 0.69-1.82 | 0.6445 |  | Hyper-methylation |
|  | **cg20016631** | 4 | *FAM114A1* | 5'UTR | 1.45 | 1.03-2.04 | 0.0313 | 0.6677 | Hyper-methylation |
|  | cg20054786 | 4 | *TLR1* | Body | 0.94 | 0.69-1.28 | 0.6769 |  | Hyper-methylation |
|  | cg03785828 | 5 | *RPS23* | Body | 0.94 | 0.72-1.24 | 0.6822 |  | Hyper-methylation |
|  | cg08198193 | 5 |  |  | 0.78 | 0.48-1.26 | 0.3088 |  | Hyper-methylation |
|  | cg12836280 | 5 |  |  | 0.79 | 0.57-1.09 | 0.1509 |  | Hyper-methylation |
|  | cg15871215 | 5 | *ATG10* | Body | 0.94 | 0.73-1.21 | 0.6464 |  | Hyper-methylation |
|  | cg16059665 | 5 |  |  | 1.26 | 0.9-1.76 | 0.1786 |  | Hyper-methylation |
|  | cg16647868 | 5 | *SLC22A5* | Body | 1.16 | 0.84-1.6 | 0.3701 |  | Hyper-methylation |
|  | cg18721397 | 5 | *SUB1* | TSS1500 | 1.01 | 0.75-1.36 | 0.9589 |  | Hyper-methylation |
|  | cg25148456 | 5 | *MAP3K1* | TSS1500 | 0.97 | 0.7-1.33 | 0.8434 |  | Hyper-methylation |
|  | cg00903577 | 6 |  |  | 1.00 | 0.74-1.35 | 0.9954 |  | Hyper-methylation |
|  | cg06608359 | 6 |  |  | 0.86 | 0.64-1.16 | 0.3171 |  | Hyper-methylation |
|  | cg12612406 | 6 | *TRIM40* | TSS1500 | 1.37 | 0.96-1.96 | 0.0823 |  | Hyper-methylation |
|  | cg12914966 | 6 |  |  | 1.01 | 0.78-1.29 | 0.9672 |  | Hyper-methylation |
|  | cg16677399 | 6 |  |  | 1.03 | 0.8-1.33 | 0.8043 |  | Hyper-methylation |
|  | cg17783140 | 6 |  |  | 1.16 | 0.85-1.58 | 0.3452 |  | Hyper-methylation |
|  | cg18745416 | 6 | *ESR1* | TSS1500 | 1.14 | 0.85-1.51 | 0.3762 |  | Hyper-methylation |
|  | cg19918208 | 6 |  |  | 0.99 | 0.74-1.32 | 0.9223 |  | Hyper-methylation |
|  | **cg22157087** | 6 | *ESR1* | 5'UTR | 0.75 | 0.58-0.98 | 0.0379 | 0.6677 | Hyper-methylation |
|  | cg27045756 | 6 | *ZNF311* | 5'UTR | 1.06 | 0.78-1.43 | 0.7119 |  | Hyper-methylation |
|  | cg01689657 | 7 | *CYP51A1* | TSS1500 | 0.83 | 0.61-1.12 | 0.2237 |  | Hyper-methylation |
|  | cg03714773 | 7 | *CYP51A1* | TSS1500 | 0.91 | 0.67-1.23 | 0.5325 |  | Hyper-methylation |
|  | cg07237996 | 7 | *TECPR1* | TSS1500 | 1.14 | 0.84-1.55 | 0.4118 |  | Hyper-methylation |
|  | cg18422172 | 7 | *CYP51A1* | TSS1500 | 0.82 | 0.6-1.11 | 0.1968 |  | Hyper-methylation |
|  | cg22117172 | 7 | *CYP51A1* | TSS1500 | 0.76 | 0.56-1.03 | 0.0752 |  | Hyper-methylation |
|  | cg01666716 | 8 |  |  | 1.03 | 0.72-1.49 | 0.8613 |  | Hyper-methylation |
|  | cg07540652 | 8 |  |  | 1.05 | 0.79-1.41 | 0.7209 |  | Hyper-methylation |
|  | cg15704662 | 8 |  |  | 1.51 | 1-2.29 | 0.0504 |  | Hyper-methylation |
|  | cg16570133 | 8 |  |  | 0.84 | 0.64-1.1 | 0.2071 |  | Hyper-methylation |
|  | cg17095489 | 8 |  |  | 0.93 | 0.67-1.29 | 0.6588 |  | Hyper-methylation |
|  | cg13634994 | 9 | *LMX1B* | Body | 0.79 | 0.6-1.05 | 0.1099 |  | Hyper-methylation |
|  | cg03610228 | 10 |  |  | 0.92 | 0.7-1.2 | 0.5208 |  | Hyper-methylation |
|  | cg04707519 | 10 |  |  | 0.80 | 0.6-1.08 | 0.1464 |  | Hyper-methylation |
|  | cg04714110 | 10 |  |  | 1.07 | 0.74-1.53 | 0.7247 |  | Hyper-methylation |
|  | cg05162358 | 10 |  |  | 1.19 | 0.85-1.66 | 0.3101 |  | Hyper-methylation |
|  | cg06082804 | 10 | *FGFR2* | Body | 0.98 | 0.75-1.3 | 0.9082 |  | Hyper-methylation |
|  | cg06874403 | 10 |  |  | 0.89 | 0.61-1.3 | 0.5479 |  | Hyper-methylation |
|  | cg12732162 | 10 |  |  | 1.30 | 0.95-1.78 | 0.1026 |  | Hyper-methylation |
|  | cg12733607 | 10 | *C10orf140* | 5'UTR | 0.77 | 0.54-1.1 | 0.1497 |  | Hyper-methylation |
|  | cg14282850 | 10 | *TACC2* | Body | 1.20 | 0.91-1.6 | 0.1957 |  | Hyper-methylation |
|  | cg15492064 | 10 |  |  | 1.16 | 0.82-1.65 | 0.3996 |  | Hyper-methylation |
|  | cg16863332 | 10 | *ADO* | 3'UTR | 1.09 | 0.78-1.53 | 0.6016 |  | Hyper-methylation |

Supplementary Table 1. Continued

| CpG sets | Probe | CHR | Gene | Region | OR^a^ | 95%CI | *P*-value | *Q*-value^b^ | Previously reported direction |
| --- | --- | --- | --- | --- | --- | --- | --- | --- | --- |
|  | cg20569108 | 10 | *MLLT10* | TSS1500 | 1.15 | 0.7-1.91 | 0.5792 |  | Hyper-methylation |
|  | cg24983858 | 10 | *ZMIZ1* | 5'UTR | 1.15 | 0.81-1.62 | 0.439 |  | Hyper-methylation |
|  | cg01097872 | 11 | *OVOL1* | Body | 1.14 | 0.81-1.59 | 0.46 |  | Hyper-methylation |
|  | cg01741372 | 11 |  |  | 1.23 | 0.97-1.57 | 0.093 |  | Hyper-methylation |
|  | cg01821149 | 11 | *TNNT3* | Body | 1.17 | 0.87-1.55 | 0.2985 |  | Hyper-methylation |
|  | cg02230254 | 11 |  |  | 0.96 | 0.73-1.27 | 0.7847 |  | Hyper-methylation |
|  | cg04111478 | 11 |  |  | 0.90 | 0.69-1.17 | 0.4275 |  | Hyper-methylation |
|  | cg04293602 | 11 | *OVOL1* | TSS1500 | 1.06 | 0.78-1.44 | 0.7099 |  | Hyper-methylation |
|  | cg04310331 | 11 | *LOC100133545* | TSS1500 | 0.91 | 0.57-1.46 | 0.705 |  | Hyper-methylation |
|  | cg04338129 | 11 | *EFEMP2* | Body | 1.11 | 0.81-1.53 | 0.5038 |  | Hyper-methylation |
|  | cg05122969 | 11 | *ANO9* | Body | 0.81 | 0.63-1.02 | 0.078 |  | Hyper-methylation |
|  | cg05928581 | 11 | *LSP1* | Body | 1.17 | 0.88-1.55 | 0.2858 |  | Hyper-methylation |
|  | cg06455375 | 11 | *TNNT3* | Body | 1.08 | 0.77-1.51 | 0.6643 |  | Hyper-methylation |
|  | cg07434944 | 11 |  |  | 1.24 | 0.94-1.64 | 0.1305 |  | Hyper-methylation |
|  | **cg07589064** | 11 |  |  | 0.68 | 0.49-0.96 | 0.0271 | 0.6677 | Hyper-methylation |
|  | cg08687540 | 11 | *LSP1* | 5'UTR | 1.14 | 0.8-1.62 | 0.4699 |  | Hyper-methylation |
|  | cg10514793 | 11 | *LRDD* | Body | 0.78 | 0.52-1.18 | 0.2331 |  | Hyper-methylation |
|  | cg10604040 | 11 | *OVOL1* | Body | 1.18 | 0.74-1.88 | 0.489 |  | Hyper-methylation |
|  | cg11465766 | 11 | *LRDD* | Body | 1.30 | 0.87-1.94 | 0.2082 |  | Hyper-methylation |
|  | **cg12038298** | 11 | *TNNT3* | Body | 2.02 | 1.04-3.92 | 0.037 | 0.6677 | Hyper-methylation |
|  | cg12723425 | 11 |  |  | 1.20 | 0.81-1.78 | 0.3531 |  | Hyper-methylation |
|  | cg13058214 | 11 | *TNNT3* | Body | 0.90 | 0.68-1.19 | 0.4442 |  | Hyper-methylation |
|  | cg14295924 | 11 |  |  | 0.96 | 0.7-1.32 | 0.7978 |  | Hyper-methylation |
|  | cg15453482 | 11 | *OVOL1* | Body | 0.98 | 0.74-1.29 | 0.8629 |  | Hyper-methylation |
|  | cg15531562 | 11 | *SNX32* | Body | 1.25 | 0.96-1.63 | 0.0906 |  | Hyper-methylation |
|  | cg15849872 | 11 | *TNNT3* | 5'UTR | 1.08 | 0.72-1.62 | 0.699 |  | Hyper-methylation |
|  | cg17551192 | 11 | *EFEMP2* | Body | 1.25 | 0.77-2.03 | 0.3762 |  | Hyper-methylation |
|  | cg17616283 | 11 | *EFEMP2* | Body | 1.18 | 0.75-1.84 | 0.471 |  | Hyper-methylation |
|  | cg18032502 | 11 | *TNNT3* | Body | 1.09 | 0.85-1.4 | 0.5112 |  | Hyper-methylation |
|  | cg18877271 | 11 | *TNNT3* | Body | 1.02 | 0.75-1.39 | 0.886 |  | Hyper-methylation |
|  | cg19502266 | 11 |  |  | 1.13 | 0.8-1.61 | 0.4942 |  | Hyper-methylation |
|  | cg19694099 | 11 | *OVOL1* | Body | 1.21 | 0.9-1.62 | 0.1983 |  | Hyper-methylation |
|  | cg20494635 | 11 | *LOC100133545* | TSS1500 | 1.01 | 0.76-1.34 | 0.9406 |  | Hyper-methylation |
|  | cg21136371 | 11 |  |  | 1.08 | 0.78-1.51 | 0.6287 |  | Hyper-methylation |
|  | cg23135908 | 11 |  |  | 1.23 | 0.87-1.73 | 0.2358 |  | Hyper-methylation |
|  | cg23220533 | 11 | *OVOL1* | TSS1500 | 0.98 | 0.67-1.43 | 0.9201 |  | Hyper-methylation |
|  | cg23754390 | 11 | *CD151* | 5'UTR | 0.78 | 0.58-1.04 | 0.0938 |  | Hyper-methylation |
|  | cg24020398 | 11 | *CCND1* | TSS200 | 1.12 | 0.81-1.55 | 0.5087 |  | Hyper-methylation |
|  | cg24660199 | 11 | *LRDD* | Body | 1.20 | 0.77-1.86 | 0.4253 |  | Hyper-methylation |
|  | cg26433582 | 11 | *TPCN2* | Body | 1.19 | 0.91-1.56 | 0.2057 |  | Hyper-methylation |
|  | cg26683025 | 11 | *OVOL1* | Body | 1.06 | 0.8-1.4 | 0.69 |  | Hyper-methylation |
|  | cg27238887 | 11 | *PNPLA2* | 3'UTR | 1.10 | 0.76-1.61 | 0.6147 |  | Hyper-methylation |
|  | cg27305772 | 11 | *MUS81* | Body | 1.03 | 0.67-1.58 | 0.9035 |  | Hyper-methylation |
|  | cg00480142 | 12 |  |  | 1.08 | 0.81-1.45 | 0.6018 |  | Hyper-methylation |
|  | cg01333011 | 12 | *PTHLH* | 5'UTR | 0.96 | 0.71-1.31 | 0.8047 |  | Hyper-methylation |
|  | cg07211768 | 12 |  |  | 1.11 | 0.84-1.45 | 0.4599 |  | Hyper-methylation |
|  | cg11827629 | 12 |  |  | 1.00 | 0.67-1.5 | 0.9937 |  | Hyper-methylation |

Supplementary Table 1. Continued

| CpG sets | Probe | CHR | Gene | Region | OR^a^ | 95%CI | *P*-value | *Q*-value^b^ | Previously reported direction |
| --- | --- | --- | --- | --- | --- | --- | --- | --- | --- |
|  | cg11891239 | 12 |  |  | 0.96 | 0.72-1.29 | 0.7956 |  | Hyper-methylation |
|  | cg16550264 | 12 |  |  | 0.83 | 0.64-1.09 | 0.1731 |  | Hyper-methylation |
|  | cg25626611 | 12 |  |  | 1.22 | 0.91-1.63 | 0.1806 |  | Hyper-methylation |
|  | **cg00828721** | 14 |  |  | 1.40 | 1.05-1.85 | 0.0201 | 0.6677 | Hyper-methylation |
|  | cg06894612 | 14 | *RAD51L1* | Body | 1.05 | 0.75-1.48 | 0.7633 |  | Hyper-methylation |
|  | cg07112884 | 14 | *RAD51L1* | Body | 1.19 | 0.91-1.56 | 0.1994 |  | Hyper-methylation |
|  | cg10395868 | 14 | *C14orf181* | TSS1500 | 1.00 | 0.67-1.49 | 0.9912 |  | Hyper-methylation |
|  | cg10975863 | 14 | *RAD51L1* | Body | 0.96 | 0.67-1.37 | 0.8299 |  | Hyper-methylation |
|  | cg11730703 | 14 | *INF2* | 5'UTR | 1.16 | 0.88-1.52 | 0.2978 |  | Hyper-methylation |
|  | cg13225565 | 14 | *RIN3* | Body | 0.89 | 0.68-1.16 | 0.3761 |  | Hyper-methylation |
|  | cg13578465 | 14 | *INF2* | 5'UTR | 1.09 | 0.84-1.42 | 0.5126 |  | Hyper-methylation |
|  | cg16255729 | 14 | *RIN3* | Body | 0.95 | 0.7-1.29 | 0.7405 |  | Hyper-methylation |
|  | cg17984022 | 14 | *INF2* | 5'UTR | 1.25 | 0.94-1.65 | 0.1244 |  | Hyper-methylation |
|  | cg22971029 | 14 | *CCDC88C* | Body | 1.13 | 0.76-1.68 | 0.5564 |  | Hyper-methylation |
|  | cg23343291 | 14 | *INF2* | Body | 1.07 | 0.74-1.53 | 0.7311 |  | Hyper-methylation |
|  | cg23998240 | 14 | *INF2* | 5'UTR | 1.19 | 0.85-1.66 | 0.3126 |  | Hyper-methylation |
|  | cg27286011 | 14 | *INF2* | 5'UTR | 1.31 | 0.93-1.83 | 0.1215 |  | Hyper-methylation |
|  | cg05878630 | 15 | *HDDC3* | Body | 0.89 | 0.67-1.19 | 0.443 |  | Hyper-methylation |
|  | cg11145826 | 15 | *HDDC3* | TSS1500 | 1.06 | 0.69-1.64 | 0.7901 |  | Hyper-methylation |
|  | cg27366007 | 15 | *UNC45A* | Body | 0.93 | 0.62-1.39 | 0.7202 |  | Hyper-methylation |
|  | cg03896542 | 16 | *GNAO1* | Body | 1.23 | 0.8-1.89 | 0.3472 |  | Hyper-methylation |
|  | cg09243909 | 16 | *FTO* | Body | 0.90 | 0.67-1.2 | 0.4809 |  | Hyper-methylation |
|  | cg09577575 | 16 |  |  | 1.13 | 0.85-1.5 | 0.4099 |  | Hyper-methylation |
|  | cg00891649 | 17 | *MAPT* | 5'UTR | 1.29 | 0.93-1.79 | 0.131 |  | Hyper-methylation |
|  | cg04491389 | 17 | *KIAA1267* | Body | 1.02 | 0.73-1.42 | 0.9113 |  | Hyper-methylation |
|  | cg04726374 | 17 | *PTRF* | 3'UTR | 1.06 | 0.76-1.5 | 0.7195 |  | Hyper-methylation |
|  | cg04927033 | 17 | *LOC644172* | Body | 1.25 | 0.91-1.71 | 0.1726 |  | Hyper-methylation |
|  | cg05159804 | 17 |  |  | 1.06 | 0.79-1.4 | 0.7107 |  | Hyper-methylation |
|  | cg05301556 | 17 | *MAPT* | TSS1500 | 1.15 | 0.87-1.51 | 0.3286 |  | Hyper-methylation |
|  | cg06291494 | 17 |  |  | 1.28 | 0.92-1.77 | 0.1408 |  | Hyper-methylation |
|  | cg06462185 | 17 |  |  | 1.20 | 0.86-1.65 | 0.2803 |  | Hyper-methylation |
|  | cg07067577 | 17 | *SH3D20* | 3'UTR | 0.83 | 0.58-1.19 | 0.3108 |  | Hyper-methylation |
|  | cg07368061 | 17 | *MAPT* | Body | 1.18 | 0.83-1.68 | 0.3437 |  | Hyper-methylation |
|  | cg07778819 | 17 | *CRHR1* | Body | 1.22 | 0.87-1.7 | 0.2553 |  | Hyper-methylation |
|  | cg09764761 | 17 | *MAPT* | 3'UTR | 1.19 | 0.86-1.64 | 0.2923 |  | Hyper-methylation |
|  | cg13732302 | 17 | *KIAA1267* | Body | 0.82 | 0.57-1.19 | 0.2923 |  | Hyper-methylation |
|  | cg15413793 | 17 | *MGC57346* | Body | 1.07 | 0.8-1.43 | 0.6583 |  | Hyper-methylation |
|  | cg16281322 | 17 | *SH3D20* | TSS200 | 1.76 | 1-3.1 | 0.05 |  | Hyper-methylation |
|  | cg17280106 | 17 |  |  | 0.94 | 0.69-1.29 | 0.7191 |  | Hyper-methylation |
|  | cg23590916 | 17 | *MGC57346* | TSS1500 | 1.07 | 0.81-1.4 | 0.6502 |  | Hyper-methylation |
|  | cg24063856 | 17 | *CRHR1* | Body | 1.08 | 0.81-1.45 | 0.6018 |  | Hyper-methylation |
|  | cg27503360 | 17 | *CRHR1* | Body | 0.96 | 0.74-1.24 | 0.7474 |  | Hyper-methylation |
|  | cg27551605 | 17 | *CRHR1* | Body | 1.30 | 0.89-1.89 | 0.1796 |  | Hyper-methylation |
|  | cg00751072 | 19 | *SSBP4* | TSS1500 | 1.17 | 0.75-1.83 | 0.4763 |  | Hyper-methylation |
|  | cg01065977 | 19 | *ISYNA1* | TSS1500 | 0.88 | 0.69-1.12 | 0.2927 |  | Hyper-methylation |
|  | cg02546618 | 19 | *KIAA0892* | TSS1500 | 0.93 | 0.66-1.31 | 0.6743 |  | Hyper-methylation |
|  | cg03719032 | 19 | *HAPLN4* | TSS1500 | 0.99 | 0.72-1.35 | 0.9322 |  | Hyper-methylation |

Supplementary Table 1. Continued

| CpG sets | Probe | CHR | Gene | Region | OR^a^ | 95%CI | *P*-value | *Q*-value^b^ | Previously reported direction |
| --- | --- | --- | --- | --- | --- | --- | --- | --- | --- |
|  | cg06953865 | 19 | *ISYNA1* | TSS1500 | 0.91 | 0.69-1.2 | 0.5056 |  | Hyper-methylation |
|  | cg07636338 | 19 | *FBXO46* | 3'UTR | 1.24 | 0.86-1.8 | 0.2509 |  | Hyper-methylation |
|  | cg08633290 | 19 |  |  | 1.06 | 0.82-1.37 | 0.6454 |  | Hyper-methylation |
|  | cg09516349 | 19 | *SSBP4* | TSS1500 | 1.02 | 0.69-1.51 | 0.9233 |  | Hyper-methylation |
|  | cg11102782 | 19 | *ISYNA1* | TSS200 | 0.99 | 0.69-1.43 | 0.9594 |  | Hyper-methylation |
|  | cg12014333 | 19 | *TRMT1* | TSS1500 | 0.93 | 0.7-1.22 | 0.5875 |  | Hyper-methylation |
|  | cg12527440 | 19 | *ZSWIM4* | Body | 1.51 | 0.99-2.31 | 0.0554 |  | Hyper-methylation |
|  | cg15073853 | 19 | *ISYNA1* | TSS200 | 0.92 | 0.66-1.28 | 0.616 |  | Hyper-methylation |
|  | cg16629695 | 19 |  |  | 0.75 | 0.5-1.13 | 0.1684 |  | Hyper-methylation |
|  | cg17327990 | 19 | *ISYNA1* | TSS200 | 0.92 | 0.64-1.32 | 0.6553 |  | Hyper-methylation |
|  | cg19640821 | 19 | *LRRC25* | Body | 0.97 | 0.55-1.72 | 0.9234 |  | Hyper-methylation |
|  | **cg21073520** | 19 | *CILP2* | TSS200 | 0.72 | 0.54-0.96 | 0.025 | 0.6677 | Hyper-methylation |
|  | cg21131022 | 19 | *HAPLN4* | Body | 0.84 | 0.63-1.12 | 0.23 |  | Hyper-methylation |
|  | **cg21956434** | 19 | *C19orf62* | TSS1500 | 1.37 | 1.06-1.78 | 0.0175 | 0.6677 | Hyper-methylation |
|  | cg21962901 | 19 | *ISYNA1* | TSS200 | 0.81 | 0.59-1.11 | 0.187 |  | Hyper-methylation |
|  | cg22161383 | 19 | *ISYNA1* | 3'UTR | 0.89 | 0.63-1.27 | 0.5352 |  | Hyper-methylation |
|  | cg23259813 | 19 | *ELL* | Body | 0.99 | 0.66-1.49 | 0.9714 |  | Hyper-methylation |
|  | cg23479742 | 19 | *CILP2* | TSS200 | 0.85 | 0.65-1.13 | 0.2634 |  | Hyper-methylation |
|  | cg25828334 | 19 | *ISYNA1* | 3'UTR | 1.16 | 0.83-1.61 | 0.3791 |  | Hyper-methylation |
|  | cg27346356 | 19 | *CILP2* | TSS200 | 0.88 | 0.68-1.14 | 0.3361 |  | Hyper-methylation |
|  | cg09146183 | 22 | *MAFF* | Body | 0.96 | 0.71-1.3 | 0.7991 |  | Hyper-methylation |
|  | cg15548613 | 22 | *MAFF* | Body | 0.90 | 0.7-1.15 | 0.382 |  | Hyper-methylation |
|  | cg16866941 | 22 | *MAFF* | 5'UTR | 0.97 | 0.69-1.36 | 0.8684 |  | Hyper-methylation |
|  | cg18884555 | 22 | *MAFF* | Body | 1.09 | 0.78-1.51 | 0.6284 |  | Hyper-methylation |
|  | cg21687563 | 22 | *TTC28* | Body | 0.76 | 0.48-1.21 | 0.2474 |  | Hyper-methylation |
|  | cg00394712 | 1 | *NUDT17* | TSS200 | 0.90 | 0.66-1.22 | 0.4952 |  | Hypo-methylation |
|  | cg01019262 | 1 |  |  | 1.32 | 0.94-1.87 | 0.1129 |  | Hypo-methylation |
|  | cg03733578 | 1 | *KLHDC7A* | 1stExon | 0.97 | 0.72-1.3 | 0.8163 |  | Hypo-methylation |
|  | cg06662140 | 1 | *KLHDC7A* | 1stExon | 0.92 | 0.72-1.16 | 0.4673 |  | Hypo-methylation |
|  | cg08949192 | 1 | *RUSC1* | TSS1500 | 0.91 | 0.66-1.25 | 0.5633 |  | Hypo-methylation |
|  | cg09141557 | 1 |  |  | 1.04 | 0.68-1.58 | 0.863 |  | Hypo-methylation |
|  | cg09579413 | 1 | *RUSC1* | TSS1500 | 1.34 | 0.9-1.99 | 0.1518 |  | Hypo-methylation |
|  | cg11175836 | 1 | *KLHDC7A* | TSS1500 | 0.89 | 0.68-1.18 | 0.4226 |  | Hypo-methylation |
|  | cg15131316 | 1 | *KLHDC7A* | 1stExon | 1.06 | 0.81-1.38 | 0.6889 |  | Hypo-methylation |
|  | cg15676040 | 1 | *RUSC1* | TSS200 | 0.99 | 0.74-1.33 | 0.94 |  | Hypo-methylation |
|  | cg18147280 | 1 | *PEX14* | Body | 0.87 | 0.64-1.18 | 0.3651 |  | Hypo-methylation |
|  | cg21693033 | 1 | *KLHDC7A* | 1stExon | 0.87 | 0.64-1.18 | 0.3662 |  | Hypo-methylation |
|  | cg22424284 | 1 | *FAM46C* | Body | 0.84 | 0.65-1.09 | 0.1902 |  | Hypo-methylation |
|  | cg23281527 | 1 | *KLHDC7A* | 1stExon | 0.95 | 0.75-1.2 | 0.655 |  | Hypo-methylation |
|  | cg23589035 | 1 | *ASH1L* | Body | 0.99 | 0.63-1.55 | 0.9622 |  | Hypo-methylation |
|  | cg24006126 | 1 | *RUSC1* | 5'UTR | 0.90 | 0.63-1.28 | 0.5612 |  | Hypo-methylation |
|  | cg24544490 | 1 | *RUSC1* | 5'UTR | 0.95 | 0.67-1.33 | 0.7485 |  | Hypo-methylation |
|  | cg24550026 | 1 | *BCL2L15* | 1stExon | 0.72 | 0.45-1.15 | 0.1713 |  | Hypo-methylation |
|  | **cg25095934** | 1 |  |  | 1.32 | 1.01-1.74 | 0.0414 | 0.7013 | Hypo-methylation |
|  | cg25197698 | 1 |  |  | 1.02 | 0.8-1.31 | 0.8772 |  | Hypo-methylation |
|  | cg25310676 | 1 | *PEX14* | Body | 1.16 | 0.74-1.8 | 0.5228 |  | Hypo-methylation |
|  | cg25767870 | 1 |  |  | 1.02 | 0.6-1.72 | 0.9464 |  | Hypo-methylation |

Supplementary Table 1. Continued

| CpG sets | Probe | CHR | Gene | Region | OR^a^ | 95%CI | *P*-value | *Q*-value^b^ | Previously reported direction |
| --- | --- | --- | --- | --- | --- | --- | --- | --- | --- |
|  | cg01396774 | 2 | *DIRC3* | Body | 1.03 | 0.75-1.43 | 0.839 |  | Hypo-methylation |
|  | cg01884057 | 2 |  |  | 0.77 | 0.59-1.01 | 0.0552 |  | Hypo-methylation |
|  | cg02168584 | 2 |  |  | 0.80 | 0.61-1.04 | 0.0965 |  | Hypo-methylation |
|  | cg12021814 | 2 |  |  | 0.97 | 0.66-1.42 | 0.8662 |  | Hypo-methylation |
|  | cg15335768 | 2 | *DIRC3* | Body | 1.09 | 0.78-1.52 | 0.6055 |  | Hypo-methylation |
|  | **cg15423357** | 2 |  |  | 0.73 | 0.56-0.96 | 0.0255 | 0.6677 | Hypo-methylation |
|  | cg16190888 | 2 | *ANTXR1* | Body | 0.98 | 0.74-1.29 | 0.8607 |  | Hypo-methylation |
|  | cg19758881 | 2 |  |  | 1.15 | 0.82-1.62 | 0.4259 |  | Hypo-methylation |
|  | cg21808287 | 2 | *TNP1* | TSS200 | 1.13 | 0.77-1.65 | 0.5387 |  | Hypo-methylation |
|  | cg22480783 | 2 |  |  | 0.92 | 0.71-1.19 | 0.5276 |  | Hypo-methylation |
|  | cg00613786 | 3 |  |  | 1.31 | 0.88-1.94 | 0.1835 |  | Hypo-methylation |
|  | cg01879723 | 3 | *ZBTB38* | 5'UTR | 0.82 | 0.57-1.18 | 0.2857 |  | Hypo-methylation |
|  | cg03060555 | 3 | *ZBTB38* | 5'UTR | 0.80 | 0.59-1.1 | 0.1659 |  | Hypo-methylation |
|  | cg03277049 | 3 |  |  | 0.88 | 0.67-1.15 | 0.3409 |  | Hypo-methylation |
|  | cg07805332 | 3 | *MIR548G* | Body | 1.01 | 0.76-1.36 | 0.9209 |  | Hypo-methylation |
|  | cg08360599 | 3 | *ZBTB38* | 5'UTR | 0.77 | 0.56-1.05 | 0.1001 |  | Hypo-methylation |
|  | cg09025210 | 3 | *ZBTB38* | 5'UTR | 0.73 | 0.43-1.23 | 0.2301 |  | Hypo-methylation |
|  | cg13411656 | 3 | *ZBTB38* | 5'UTR | 0.94 | 0.68-1.3 | 0.7096 |  | Hypo-methylation |
|  | **cg14500070** | 3 | *ZBTB38* | 5'UTR | 0.60 | 0.38-0.95 | 0.029 | 0.6677 | Hypo-methylation |
|  | cg17388683 | 3 | *MIR548G* | Body | 0.86 | 0.63-1.16 | 0.3265 |  | Hypo-methylation |
|  | cg17581925 | 3 |  |  | 1.20 | 0.8-1.78 | 0.3772 |  | Hypo-methylation |
|  | cg20395773 | 3 | *ZBTB38* | 5'UTR | 1.07 | 0.8-1.45 | 0.6386 |  | Hypo-methylation |
|  | cg20959189 | 3 | *ZBTB38* | 5'UTR | 1.04 | 0.62-1.74 | 0.8882 |  | Hypo-methylation |
|  | cg21593952 | 3 |  |  | 0.82 | 0.57-1.16 | 0.2544 |  | Hypo-methylation |
|  | cg26133301 | 3 |  |  | 0.96 | 0.71-1.29 | 0.7775 |  | Hypo-methylation |
|  | cg26764555 | 3 | *RASA2* | TSS1500 | 1.08 | 0.81-1.44 | 0.6201 |  | Hypo-methylation |
|  | cg27288595 | 3 | *ZBTB38* | 5'UTR | 1.31 | 0.91-1.87 | 0.1484 |  | Hypo-methylation |
|  | cg22839308 | 4 | *TLR1* | TSS1500 | 0.88 | 0.65-1.19 | 0.3978 |  | Hypo-methylation |
|  | cg00308841 | 5 |  |  | 0.83 | 0.61-1.12 | 0.2193 |  | Hypo-methylation |
|  | cg02648101 | 5 | *ATP6AP1L* | TSS1500 | 0.99 | 0.73-1.35 | 0.9723 |  | Hypo-methylation |
|  | cg07807695 | 5 | *ATP6AP1L* | Body | 1.07 | 0.81-1.42 | 0.6227 |  | Hypo-methylation |
|  | cg12308054 | 5 | *HSPA4* | TSS1500 | 1.02 | 0.77-1.35 | 0.8784 |  | Hypo-methylation |
|  | cg13663945 | 5 | *ATP6AP1L* | TSS1500 | 1.09 | 0.82-1.45 | 0.5478 |  | Hypo-methylation |
|  | cg16971831 | 5 | *MAP3K1* | 1stExon | 1.04 | 0.77-1.4 | 0.8193 |  | Hypo-methylation |
|  | cg17277001 | 5 | *SLC6A18* | Body | 1.23 | 0.94-1.6 | 0.1287 |  | Hypo-methylation |
|  | cg17942617 | 5 | *ATG10* | Body | 1.10 | 0.81-1.49 | 0.5299 |  | Hypo-methylation |
|  | cg18294332 | 5 | *MAP3K1* | Body | 1.14 | 0.83-1.55 | 0.4212 |  | Hypo-methylation |
|  | cg19040266 | 5 | *SLC22A5* | Body | 1.07 | 0.76-1.51 | 0.6851 |  | Hypo-methylation |
|  | cg21155100 | 5 | *MAP3K1* | Body | 1.24 | 0.87-1.76 | 0.2342 |  | Hypo-methylation |
|  | cg22692507 | 5 | *ATG10* | Body | 1.11 | 0.78-1.56 | 0.5624 |  | Hypo-methylation |
|  | cg25363080 | 5 | *ATG10* | Body | 1.30 | 0.65-2.61 | 0.4525 |  | Hypo-methylation |
|  | cg27303933 | 5 |  |  | 0.95 | 0.62-1.45 | 0.811 |  | Hypo-methylation |
|  | cg00609961 | 6 |  |  | 1.18 | 0.85-1.63 | 0.3223 |  | Hypo-methylation |
|  | cg02004594 | 6 | *ZNF311* | 5'UTR | 0.94 | 0.69-1.27 | 0.678 |  | Hypo-methylation |
|  | cg03714916 | 6 | *CDKN1A* | TSS1500 | 1.12 | 0.72-1.74 | 0.6213 |  | Hypo-methylation |
|  | cg04012857 | 6 | *TRIM31* | Body | 1.03 | 0.78-1.35 | 0.8386 |  | Hypo-methylation |
|  | cg05216056 | 6 | *TRIM27* | Body | 0.88 | 0.58-1.34 | 0.5524 |  | Hypo-methylation |

Supplementary Table 1. Continued

| CpG sets | Probe | CHR | Gene | Region | OR^a^ | 95%CI | *P*-value | *Q*-value^b^ | Previously reported direction |
| --- | --- | --- | --- | --- | --- | --- | --- | --- | --- |
|  | cg08125020 | 6 | *TRIM27* | Body | 1.10 | 0.8-1.52 | 0.5564 |  | Hypo-methylation |
|  | cg10046620 | 6 | *HIST1H2AI* | TSS1500 | 0.92 | 0.66-1.26 | 0.5906 |  | Hypo-methylation |
|  | cg10433043 | 6 |  |  | 1.04 | 0.79-1.36 | 0.7922 |  | Hypo-methylation |
|  | cg11920449 | 6 | *CDKN1A* | TSS1500 | 1.00 | 0.76-1.32 | 0.975 |  | Hypo-methylation |
|  | cg12078157 | 6 | *SIRT5* | 3'UTR | 0.97 | 0.75-1.26 | 0.8179 |  | Hypo-methylation |
|  | cg14781281 | 6 | *HLA-J* | Body | 0.83 | 0.61-1.14 | 0.2493 |  | Hypo-methylation |
|  | cg15626350 | 6 | *ESR1* | Body | 0.90 | 0.69-1.16 | 0.4202 |  | Hypo-methylation |
|  | cg18784558 | 6 |  |  | 0.84 | 0.61-1.17 | 0.3034 |  | Hypo-methylation |
|  | cg19727175 | 6 |  |  | 0.85 | 0.63-1.15 | 0.2827 |  | Hypo-methylation |
|  | cg24425727 | 6 | *CDKN1A* | TSS1500 | 1.00 | 0.76-1.31 | 0.9825 |  | Hypo-methylation |
|  | cg25701364 | 6 | *TRIM27* | 3'UTR | 1.28 | 0.87-1.88 | 0.211 |  | Hypo-methylation |
|  | cg02729383 | 7 |  |  | 1.00 | 0.77-1.31 | 0.9825 |  | Hypo-methylation |
|  | cg14761417 | 7 | *FLJ43663* | Body | 1.33 | 0.88-2.03 | 0.1793 |  | Hypo-methylation |
|  | cg23235069 | 7 |  |  | 1.14 | 0.88-1.48 | 0.3323 |  | Hypo-methylation |
|  | cg18896184 | 8 |  |  | 1.02 | 0.77-1.34 | 0.8955 |  | Hypo-methylation |
|  | cg01628053 | 10 |  |  | 1.14 | 0.86-1.5 | 0.3568 |  | Hypo-methylation |
|  | cg04231319 | 10 | *MLLT10* | Body | 1.48 | 0.91-2.39 | 0.1131 |  | Hypo-methylation |
|  | cg05399434 | 10 |  |  | 0.93 | 0.69-1.24 | 0.6133 |  | Hypo-methylation |
|  | cg10812439 | 10 | *ZMIZ1* | 5'UTR | 1.28 | 0.92-1.77 | 0.1383 |  | Hypo-methylation |
|  | cg14701867 | 10 | *ZNF365* | Body | 0.96 | 0.71-1.31 | 0.8147 |  | Hypo-methylation |
|  | cg17065712 | 10 | *ZMIZ1* | 5'UTR | 0.80 | 0.56-1.13 | 0.2065 |  | Hypo-methylation |
|  | cg23502253 | 10 | *LOC283050* | Body | 1.31 | 0.91-1.87 | 0.1429 |  | Hypo-methylation |
|  | cg24982682 | 10 | *ZMIZ1* | 5'UTR | 1.40 | 0.76-2.58 | 0.2843 |  | Hypo-methylation |
|  | cg00211115 | 11 |  |  | 0.90 | 0.63-1.29 | 0.5544 |  | Hypo-methylation |
|  | cg01969701 | 11 |  |  | 0.86 | 0.67-1.12 | 0.2661 |  | Hypo-methylation |
|  | cg02554274 | 11 | *CEND1* | Body | 0.88 | 0.67-1.16 | 0.3791 |  | Hypo-methylation |
|  | cg03483046 | 11 | *LSP1* | 5'UTR | 0.96 | 0.7-1.32 | 0.815 |  | Hypo-methylation |
|  | cg03982897 | 11 |  |  | 1.04 | 0.69-1.57 | 0.8579 |  | Hypo-methylation |
|  | **cg04024572** | 11 | *CCDC85B* | TSS200 | 0.73 | 0.55-0.97 | 0.0313 | 0.6677 | Hypo-methylation |
|  | cg04054921 | 11 |  |  | 0.96 | 0.73-1.28 | 0.8014 |  | Hypo-methylation |
|  | cg04647234 | 11 | *H19* | Body | 1.29 | 0.93-1.78 | 0.1225 |  | Hypo-methylation |
|  | cg06065225 | 11 | *EFEMP2* | 5'UTR | 0.82 | 0.6-1.11 | 0.1989 |  | Hypo-methylation |
|  | cg06757133 | 11 |  |  | 1.22 | 0.79-1.89 | 0.3755 |  | Hypo-methylation |
|  | cg07241660 | 11 |  |  | 0.82 | 0.62-1.09 | 0.1708 |  | Hypo-methylation |
|  | cg07977153 | 11 | *MRPL23* | TSS1500 | 0.92 | 0.61-1.38 | 0.685 |  | Hypo-methylation |
|  | cg08046411 | 11 |  |  | 1.19 | 0.85-1.65 | 0.3127 |  | Hypo-methylation |
|  | cg09055519 | 11 |  |  | 1.03 | 0.77-1.38 | 0.833 |  | Hypo-methylation |
|  | cg09177106 | 11 |  |  | 0.89 | 0.65-1.2 | 0.4356 |  | Hypo-methylation |
|  | cg10113191 | 11 | *MRPL23* | Body | 1.19 | 0.79-1.8 | 0.4122 |  | Hypo-methylation |
|  | cg11637721 | 11 | *KAT5* | 3'UTR | 0.90 | 0.67-1.21 | 0.4694 |  | Hypo-methylation |
|  | cg11955198 | 11 |  |  | 0.98 | 0.73-1.32 | 0.8956 |  | Hypo-methylation |
|  | cg13988440 | 11 |  |  | 0.85 | 0.49-1.47 | 0.5671 |  | Hypo-methylation |
|  | cg15114474 | 11 |  |  | 0.82 | 0.61-1.11 | 0.1968 |  | Hypo-methylation |
|  | cg15269875 | 11 | *H19* | Body | 1.33 | 0.96-1.83 | 0.0839 |  | Hypo-methylation |
|  | cg15317267 | 11 | *H19* | Body | 1.20 | 0.77-1.89 | 0.4188 |  | Hypo-methylation |
|  | **cg15442037** | 11 |  |  | 0.72 | 0.52-0.99 | 0.0454 | 0.7324 | Hypo-methylation |
|  | cg16073427 | 11 | *CCDC85B* | TSS200 | 1.26 | 0.91-1.76 | 0.1669 |  | Hypo-methylation |

Supplementary Table 1. Continued

| CpG sets | Probe | CHR | Gene | Region | OR^a^ | 95%CI | *P*-value | *Q*-value^b^ | Previously reported direction |
| --- | --- | --- | --- | --- | --- | --- | --- | --- | --- |
|  | cg16133872 | 11 |  |  | 0.96 | 0.71-1.29 | 0.7769 |  | Hypo-methylation |
|  | cg16151795 | 11 |  |  | 0.94 | 0.7-1.28 | 0.715 |  | Hypo-methylation |
|  | cg17293600 | 11 | *SNX32* | Body | 1.12 | 0.81-1.55 | 0.4909 |  | Hypo-methylation |
|  | cg17628894 | 11 | *LRDD* | 5'UTR | 0.94 | 0.7-1.25 | 0.655 |  | Hypo-methylation |
|  | cg17976121 | 11 |  |  | 0.91 | 0.67-1.25 | 0.5752 |  | Hypo-methylation |
|  | cg18297705 | 11 |  |  | 1.08 | 0.73-1.59 | 0.7105 |  | Hypo-methylation |
|  | **cg18498241** | 11 |  |  | 0.73 | 0.56-0.95 | 0.0195 | 0.6677 | Hypo-methylation |
|  | cg19480699 | 11 | *LRDD* | TSS1500 | 1.14 | 0.82-1.56 | 0.4385 |  | Hypo-methylation |
|  | cg20225915 | 11 | *LRDD* | TSS1500 | 1.05 | 0.71-1.55 | 0.82 |  | Hypo-methylation |
|  | **cg20781238** | 11 | *CEND1* | 3'UTR | 1.39 | 1.06-1.83 | 0.0163 | 0.6677 | Hypo-methylation |
|  | cg22009923 | 11 | *CD151* | TSS1500 | 1.44 | 0.99-2.11 | 0.0592 |  | Hypo-methylation |
|  | cg22360649 | 11 | *CCDC85B* | TSS200 | 0.86 | 0.62-1.19 | 0.3617 |  | Hypo-methylation |
|  | cg22649187 | 11 |  |  | 1.05 | 0.79-1.39 | 0.7542 |  | Hypo-methylation |
|  | cg23977670 | 11 | *H19* | Body | 1.18 | 0.84-1.67 | 0.3359 |  | Hypo-methylation |
|  | cg24193659 | 11 | *CEND1* | Body | 0.86 | 0.67-1.1 | 0.2336 |  | Hypo-methylation |
|  | cg25169679 | 11 |  |  | 0.94 | 0.74-1.2 | 0.6321 |  | Hypo-methylation |
|  | cg25852472 | 11 | *H19* | Body | 1.25 | 0.88-1.77 | 0.208 |  | Hypo-methylation |
|  | cg26015384 | 11 | *EFCAB4A* | 5'UTR | 1.36 | 0.94-1.97 | 0.0995 |  | Hypo-methylation |
|  | cg26318265 | 11 |  |  | 0.95 | 0.66-1.36 | 0.7642 |  | Hypo-methylation |
|  | cg26326607 | 11 | *MRPL23* | Body | 1.27 | 0.94-1.71 | 0.1136 |  | Hypo-methylation |
|  | **cg26509915** | 11 |  |  | 1.49 | 1.03-2.15 | 0.0333 | 0.6677 | Hypo-methylation |
|  | cg26591679 | 11 |  |  | 0.98 | 0.68-1.42 | 0.9175 |  | Hypo-methylation |
|  | cg07711192 | 12 |  |  | 1.27 | 0.81-1.99 | 0.305 |  | Hypo-methylation |
|  | cg13239976 | 12 |  |  | 1.06 | 0.75-1.48 | 0.7522 |  | Hypo-methylation |
|  | cg24428144 | 12 |  |  | 1.11 | 0.85-1.44 | 0.442 |  | Hypo-methylation |
|  | cg00787180 | 14 | *CCDC88C* | Body | 0.88 | 0.64-1.21 | 0.4318 |  | Hypo-methylation |
|  | cg13803234 | 14 | *RAD51L1* | Body | 1.11 | 0.79-1.56 | 0.5464 |  | Hypo-methylation |
|  | cg20580673 | 14 |  |  | 1.04 | 0.69-1.57 | 0.8489 |  | Hypo-methylation |
|  | cg23526087 | 14 | *RAD51L1* | Body | 1.12 | 0.52-2.45 | 0.7675 |  | Hypo-methylation |
|  | cg22570213 | 15 | *RCCD1* | TSS1500 | 1.04 | 0.78-1.4 | 0.7714 |  | Hypo-methylation |
|  | cg23684204 | 15 | *RCCD1* | TSS200 | 1.11 | 0.81-1.51 | 0.5293 |  | Hypo-methylation |
|  | cg25839482 | 15 | *IMP3* | 3'UTR | 1.11 | 0.83-1.47 | 0.4755 |  | Hypo-methylation |
|  | cg02793451 | 16 | *TOX3* | TSS1500 | 1.28 | 0.98-1.68 | 0.0688 |  | Hypo-methylation |
|  | cg09032423 | 16 | *ADCY9* | 3'UTR | 1.37 | 0.84-2.23 | 0.2124 |  | Hypo-methylation |
|  | cg00480298 | 17 | *MAPT* | Body | 1.14 | 0.85-1.52 | 0.3925 |  | Hypo-methylation |
|  | cg00971050 | 17 | *PTRF* | Body | 0.73 | 0.53-1.01 | 0.0601 |  | Hypo-methylation |
|  | cg01882395 | 17 | *C17orf69* | Body | 0.90 | 0.65-1.24 | 0.5119 |  | Hypo-methylation |
|  | cg01934064 | 17 | *MAPT* | Body | 0.89 | 0.64-1.25 | 0.5125 |  | Hypo-methylation |
|  | cg03383056 | 17 |  |  | 1.36 | 0.93-2 | 0.1145 |  | Hypo-methylation |
|  | cg03836283 | 17 | *MAPT* | Body | 0.80 | 0.54-1.16 | 0.2354 |  | Hypo-methylation |
|  | cg03915738 | 17 |  |  | 0.89 | 0.63-1.26 | 0.515 |  | Hypo-methylation |
|  | cg03954353 | 17 | *C17orf69* | TSS1500 | 0.91 | 0.66-1.24 | 0.5408 |  | Hypo-methylation |
|  | cg04703951 | 17 |  |  | 0.94 | 0.74-1.2 | 0.6288 |  | Hypo-methylation |
|  | cg05772917 | 17 | *MAPT* | 5'UTR | 0.89 | 0.62-1.28 | 0.5198 |  | Hypo-methylation |
|  | cg08113562 | 17 | *SH3D20* | 5'UTR | 0.92 | 0.71-1.2 | 0.5278 |  | Hypo-methylation |
|  | cg10955972 | 17 | *MAPT* | 5'UTR | 0.87 | 0.63-1.2 | 0.3917 |  | Hypo-methylation |
|  | cg12609785 | 17 |  |  | 0.88 | 0.68-1.13 | 0.3084 |  | Hypo-methylation |

Supplementary Table 1. Continued

| CpG sets | Probe | CHR | Gene | Region | OR^a^ | 95%CI | *P*-value | *Q*-value^b^ | Previously reported direction |
| --- | --- | --- | --- | --- | --- | --- | --- | --- | --- |
|  | cg14517863 | 17 |  |  | 0.91 | 0.64-1.3 | 0.6095 |  | Hypo-methylation |
|  | cg16652462 | 17 | *LOC644172* | Body | 0.85 | 0.66-1.1 | 0.2201 |  | Hypo-methylation |
|  | cg17347326 | 17 |  |  | 0.94 | 0.66-1.34 | 0.7227 |  | Hypo-methylation |
|  | cg18027529 | 17 |  |  | 0.76 | 0.55-1.05 | 0.0938 |  | Hypo-methylation |
|  | cg18878992 | 17 | *MAPT* | 5'UTR | 1.08 | 0.85-1.36 | 0.5301 |  | Hypo-methylation |
|  | cg19832721 | 17 | *KIAA1267* | TSS1500 | 0.79 | 0.6-1.03 | 0.0786 |  | Hypo-methylation |
|  | cg23659289 | 17 | *ARHGAP27* | 3'UTR | 0.94 | 0.61-1.45 | 0.7793 |  | Hypo-methylation |
|  | cg23955979 | 17 |  |  | 0.97 | 0.67-1.4 | 0.8728 |  | Hypo-methylation |
|  | cg24677220 | 17 | *MAPT* | Body | 0.99 | 0.73-1.33 | 0.9433 |  | Hypo-methylation |
|  | cg24910739 | 17 | *ARL17A* | TSS1500 | 0.78 | 0.59-1.04 | 0.0941 |  | Hypo-methylation |
|  | cg26471390 | 17 | *SH3D20* | TSS1500 | 0.96 | 0.73-1.26 | 0.7595 |  | Hypo-methylation |
|  | cg05300248 | 18 | *CHST9* | Body | 0.93 | 0.68-1.27 | 0.6481 |  | Hypo-methylation |
|  | cg12776287 | 18 | *KCTD1* | 5'UTR | 1.25 | 0.91-1.72 | 0.1685 |  | Hypo-methylation |
|  | cg19738924 | 18 | *KCTD1* | 5'UTR | 1.19 | 0.86-1.64 | 0.2955 |  | Hypo-methylation |
|  | cg00515669 | 19 | *LYPD5* | TSS1500 | 1.34 | 0.95-1.88 | 0.0912 |  | Hypo-methylation |
|  | **cg01297721** | 19 | *HAPLN4* | 5'UTR | 0.74 | 0.56-0.98 | 0.0334 | 0.6677 | Hypo-methylation |
|  | cg02528768 | 19 | *ISYNA1* | Body | 0.96 | 0.64-1.45 | 0.8493 |  | Hypo-methylation |
|  | cg02587156 | 19 | *ZNF45* | Body | 1.04 | 0.74-1.47 | 0.8083 |  | Hypo-methylation |
|  | cg03540731 | 19 | *LRRC25* | 1stExon | 1.02 | 0.79-1.32 | 0.8598 |  | Hypo-methylation |
|  | cg04590610 | 19 | *LYPD5* | Body | 0.98 | 0.74-1.31 | 0.9122 |  | Hypo-methylation |
|  | cg04833845 | 19 | *KCNN4* | TSS1500 | 0.98 | 0.64-1.51 | 0.9237 |  | Hypo-methylation |
|  | cg06420512 | 19 |  |  | 1.15 | 0.82-1.6 | 0.4214 |  | Hypo-methylation |
|  | cg06899329 | 19 | *NDUFA13* | Body | 1.31 | 0.96-1.8 | 0.0907 |  | Hypo-methylation |
|  | cg08331981 | 19 | *NDUFA13* | TSS1500 | 1.03 | 0.78-1.36 | 0.8273 |  | Hypo-methylation |
|  | cg08505228 | 19 | *GATAD2A* | TSS1500 | 0.88 | 0.65-1.18 | 0.3942 |  | Hypo-methylation |
|  | cg08509172 | 19 | *TM6SF2* | Body | 0.81 | 0.58-1.15 | 0.2448 |  | Hypo-methylation |
|  | cg11778563 | 19 | *SSBP4* | Body | 1.31 | 0.98-1.76 | 0.0706 |  | Hypo-methylation |
|  | **cg12163845** | 19 | *HAPLN4* | TSS200 | 0.73 | 0.55-0.96 | 0.0257 | 0.6677 | Hypo-methylation |
|  | cg13364410 | 19 | *HAPLN4* | TSS200 | 0.88 | 0.63-1.23 | 0.4711 |  | Hypo-methylation |
|  | cg14066757 | 19 | *KCNN4* | TSS200 | 1.00 | 0.67-1.49 | 0.9935 |  | Hypo-methylation |
|  | cg14449575 | 19 | *HAPLN4* | TSS200 | 0.83 | 0.57-1.19 | 0.3023 |  | Hypo-methylation |
|  | cg14602471 | 19 |  |  | 0.99 | 0.7-1.42 | 0.972 |  | Hypo-methylation |
|  | **cg15071133** | 19 | *KIAA0892* | TSS200 | 1.48 | 1.08-2.03 | 0.0142 | 0.6677 | Hypo-methylation |
|  | **cg15977816** | 19 | *KCNN4* | 1stExon | 1.51 | 1.11-2.07 | 0.0087 | 0.6677 | Hypo-methylation |
|  | cg16082401 | 19 | *GIPR* | Body | 0.76 | 0.52-1.1 | 0.1407 |  | Hypo-methylation |
|  | cg18506018 | 19 | *KCNN4* | TSS1500 | 1.09 | 0.69-1.73 | 0.6992 |  | Hypo-methylation |
|  | **cg18735402** | 19 | *GIPR* | Body | 0.62 | 0.45-0.87 | 0.0051 | 0.6677 | Hypo-methylation |
|  | **cg19822309** | 19 | *GIPR* | Body | 0.72 | 0.52-0.98 | 0.0358 | 0.6677 | Hypo-methylation |
|  | **cg20434926** | 19 | *GIPR* | Body | 0.70 | 0.51-0.97 | 0.0302 | 0.6677 | Hypo-methylation |
|  | cg21496419 | 19 | *LYPD5* | 5'UTR | 0.97 | 0.74-1.28 | 0.851 |  | Hypo-methylation |
|  | cg21757127 | 19 |  |  | 0.96 | 0.66-1.39 | 0.8101 |  | Hypo-methylation |
|  | cg22005401 | 19 | *SSBP4* | Body | 0.84 | 0.57-1.25 | 0.4015 |  | Hypo-methylation |
|  | **cg22694191** | 19 | *HAPLN4* | 5'UTR | 0.71 | 0.53-0.95 | 0.0213 | 0.6677 | Hypo-methylation |
|  | cg22813366 | 19 | *ANO8* | Body | 0.76 | 0.56-1.03 | 0.0729 |  | Hypo-methylation |
|  | cg22904711 | 19 | *KCNN4* | Body | 0.87 | 0.66-1.14 | 0.3158 |  | Hypo-methylation |
|  | cg24170529 | 20 |  |  | 1.02 | 0.7-1.48 | 0.9148 |  | Hypo-methylation |
|  | cg01089751 | 22 | *APOBEC3B* | 1stExon | 1.12 | 0.78-1.6 | 0.532 |  | Hypo-methylation |

Supplementary Table 1. Continued

| CpG sets | Probe | CHR | Gene | Region | OR^a^ | 95%CI | *P*-value | *Q*-value^b^ | Previously reported direction |
| --- | --- | --- | --- | --- | --- | --- | --- | --- | --- |
|  | cg03162506 | 22 |  |  | 1.10 | 0.78-1.56 | 0.5812 |  | Hypo-methylation |
|  | cg09232727 | 22 | *HSCB* | Body | 0.91 | 0.57-1.46 | 0.7048 |  | Hypo-methylation |
|  | cg16093065 | 22 | *TNRC6B* | 3'UTR | 1.32 | 0.75-2.34 | 0.3402 |  | Hypo-methylation |
|  | cg18940763 | 22 | *XBP1* | TSS1500 | 0.88 | 0.58-1.32 | 0.5241 |  | Hypo-methylation |
| 248-CpGs  (Joo et al., 2018; van Veldhoven et al., 2015; Xu et al., 2013) |  |  |  |  |  |  |  |  |  |
|  | cg12082615 | 1 | *SETDB1* | TSS200 | 0.81 | 0.56-1.16 | 0.2458 |  | Hyper-methylation |
|  | cg15422784 | 1 | *LRRC8B* | 5'UTR | 1.30 | 0.94-1.8 | 0.1178 |  | Hyper-methylation |
|  | cg16148270 | 1 | *DIRAS3* | TSS200 | 1.07 | 0.74-1.55 | 0.7262 |  | Hyper-methylation |
|  | cg18758482 | 1 | *RBBP4* | TSS1500 | 1.20 | 0.69-2.07 | 0.5203 |  | Hyper-methylation |
|  | cg22857957 | 1 | *FMO5* | 5'UTR | 0.91 | 0.67-1.23 | 0.5369 |  | Hyper-methylation |
|  | cg22983885 | 1 | *C1orf128* | TSS1500 | 1.12 | 0.78-1.62 | 0.539 |  | Hyper-methylation |
|  | cg01741999 | 2 | *PNKD* | Body | 1.03 | 0.78-1.37 | 0.8292 |  | Hyper-methylation |
|  | cg02397720 | 2 |  |  | 1.12 | 0.82-1.53 | 0.4597 |  | Hyper-methylation |
|  | cg03539474 | 2 | *SLC25A12* | TSS1500 | 0.93 | 0.69-1.27 | 0.6596 |  | Hyper-methylation |
|  | cg14372394 | 2 | *CHST10* | 5'UTR | 0.98 | 0.72-1.35 | 0.9199 |  | Hyper-methylation |
|  | cg18584561 | 2 | *GREB1* | TSS1500 | 1.08 | 0.85-1.38 | 0.5424 |  | Hyper-methylation |
|  | cg21388029 | 2 | *CREG2* | Body | 1.22 | 0.87-1.72 | 0.2499 |  | Hyper-methylation |
|  | cg11237115 | 3 | *KLHL24* | 5'UTR | 1.05 | 0.76-1.45 | 0.7737 |  | Hyper-methylation |
|  | cg15814508 | 3 | *P2RY14* | 1stExon | 0.97 | 0.57-1.62 | 0.8936 |  | Hyper-methylation |
|  | cg22842233 | 3 | *UPK1B* | TSS1500 | 1.12 | 0.84-1.51 | 0.4425 |  | Hyper-methylation |
|  | cg08704509 | 5 | *GRAMD3* | Body | 0.88 | 0.64-1.2 | 0.4056 |  | Hyper-methylation |
|  | cg20311501 | 5 | *APC* | TSS200 | 1.07 | 0.78-1.47 | 0.6897 |  | Hyper-methylation |
|  | cg22230395 | 5 | *FAM114A2* | 5'UTR | 1.12 | 0.79-1.58 | 0.5253 |  | Hyper-methylation |
|  | cg17067528 | 6 | *IER3* | TSS200 | 0.99 | 0.72-1.36 | 0.95 |  | Hyper-methylation |
|  | cg23566335 | 8 | *ADAM18* | Body | 1.00 | 0.67-1.49 | 0.989 |  | Hyper-methylation |
|  | cg24115571 | 8 | *FZD6* | TSS1500 | 0.86 | 0.6-1.23 | 0.4053 |  | Hyper-methylation |
|  | cg00729541 | 9 | *FAM73B* | TSS1500 | 1.01 | 0.71-1.43 | 0.955 |  | Hyper-methylation |
|  | cg11639651 | 10 | *HKDC1* | TSS1500 | 1.18 | 0.86-1.63 | 0.3043 |  | Hyper-methylation |
|  | cg16779976 | 10 | *BLNK* | 1stExon | 0.87 | 0.64-1.18 | 0.3743 |  | Hyper-methylation |
|  | cg21750602 | 10 | *PPP2R2D* | 1stExon | 1.02 | 0.74-1.39 | 0.9255 |  | Hyper-methylation |
|  | cg27555776 | 10 | *OPTN* | TSS1500 | 0.97 | 0.68-1.38 | 0.8538 |  | Hyper-methylation |
|  | cg27648946 | 10 | *RHOBTB1* | Body | 1.02 | 0.72-1.46 | 0.8978 |  | Hyper-methylation |
|  | cg00066153 | 11 | *ACRV1* | 1stExon | 0.89 | 0.64-1.22 | 0.4568 |  | Hyper-methylation |
|  | cg06790862 | 11 | *MPPED2* | Body | 1.34 | 0.85-2.12 | 0.2057 |  | Hyper-methylation |
|  | cg13838528 | 11 | *RPL27A* | Body | 1.13 | 0.83-1.52 | 0.4434 |  | Hyper-methylation |
|  | cg17022914 | 11 | *BATF2* | TSS200 | 0.92 | 0.62-1.36 | 0.6831 |  | Hyper-methylation |
|  | cg01291404 | 12 | *COL2A1* | Body | 0.86 | 0.6-1.22 | 0.4 |  | Hyper-methylation |
|  | cg18344063 | 12 | *MGAT4C* | 1stExon | 0.99 | 0.78-1.26 | 0.967 |  | Hyper-methylation |
|  | **cg27533013** | 13 | *TRIM13* | 3'UTR | 1.39 | 1.03-1.88 | 0.0339 | 0.9848 | Hyper-methylation |
|  | cg18555117 | 14 | *DDHD1* | TSS1500 | 1.11 | 0.81-1.51 | 0.5179 |  | Hyper-methylation |
|  | cg25775449 | 14 | *LTB4R* | 5'UTR | 0.88 | 0.62-1.25 | 0.475 |  | Hyper-methylation |
|  | cg17037924 | 15 | *DUT* | TSS1500 | 0.89 | 0.66-1.2 | 0.4397 |  | Hyper-methylation |

Supplementary Table 1. Continued

| CpG sets | Probe | CHR | Gene | Region | OR^a^ | 95%CI | *P*-value | *Q*-value^b^ | Previously reported direction |
| --- | --- | --- | --- | --- | --- | --- | --- | --- | --- |
|  | cg19510698 | 15 | *ALDH1A3* | Body | 0.96 | 0.73-1.26 | 0.7837 |  | Hyper-methylation |
|  | cg27639199 | 15 | *TMC3* | TSS200 | 0.88 | 0.69-1.14 | 0.3368 |  | Hyper-methylation |
|  | cg03217964 | 16 | *ZFP1* | TSS1500 | 0.81 | 0.58-1.14 | 0.2231 |  | Hyper-methylation |
|  | cg15755548 | 16 | *ARHGDIG* | Body | 1.00 | 0.73-1.38 | 0.9824 |  | Hyper-methylation |
|  | cg18160978 | 16 | *ZNF646* | TSS1500 | 1.10 | 0.8-1.53 | 0.5473 |  | Hyper-methylation |
|  | cg20083730 | 16 | *MT1E* | 1stExon | 0.99 | 0.71-1.37 | 0.9496 |  | Hyper-methylation |
|  | cg06723357 | 17 | *CUEDC1* | TSS1500 | 1.23 | 0.85-1.78 | 0.2722 |  | Hyper-methylation |
|  | cg07554030 | 17 | *NAGLU* | TSS200 | 1.07 | 0.76-1.51 | 0.6961 |  | Hyper-methylation |
|  | cg14275779 | 17 | *PLEKHH3* | TSS1500 | 1.12 | 0.77-1.62 | 0.5495 |  | Hyper-methylation |
|  | cg15077637 | 17 | *EFTUD2* | TSS1500 | 0.75 | 0.53-1.06 | 0.1038 |  | Hyper-methylation |
|  | cg18190433 | 17 |  |  | 1.27 | 0.93-1.74 | 0.1366 |  | Hyper-methylation |
|  | cg12432709 | 18 | *CCDC68* | TSS1500 | 1.17 | 0.87-1.59 | 0.3014 |  | Hyper-methylation |
|  | cg25560840 | 18 | *KIAA0427* | 5'UTR | 1.06 | 0.76-1.48 | 0.7237 |  | Hyper-methylation |
|  | cg04558861 | 19 | *LIN37* | TSS200 | 1.03 | 0.72-1.46 | 0.8855 |  | Hyper-methylation |
|  | cg05951993 | 19 | *GYS1* | TSS1500 | 0.97 | 0.68-1.4 | 0.8873 |  | Hyper-methylation |
|  | cg07072643 | 19 | *EMR3* | 5'UTR | 0.76 | 0.49-1.16 | 0.2031 |  | Hyper-methylation |
|  | cg09712606 | 19 | *IRF3* | TSS200 | 0.88 | 0.64-1.22 | 0.4517 |  | Hyper-methylation |
|  | cg25234963 | 19 | *SFRS14* | 5'UTR | 1.28 | 0.88-1.87 | 0.1986 |  | Hyper-methylation |
|  | cg00980978 | 20 | *WFDC2* | Body | 1.03 | 0.75-1.41 | 0.8619 |  | Hyper-methylation |
|  | cg08623947 | 20 | *HCK* | 1stExon | 1.07 | 0.77-1.5 | 0.6779 |  | Hyper-methylation |
|  | cg23272214 | 21 | *PCBP3* | TSS200 | 0.86 | 0.61-1.22 | 0.4086 |  | Hyper-methylation |
|  | cg00124920 | 1 | *C1orf220* | Body | 1.04 | 0.76-1.41 | 0.8224 |  | Hypo-methylation |
|  | cg03825921 | 1 | *RAB4A* | Body | 0.94 | 0.69-1.26 | 0.6633 |  | Hypo-methylation |
|  | cg07437033 | 1 | *ZBTB17* | 5'UTR | 1.16 | 0.87-1.55 | 0.2996 |  | Hypo-methylation |
|  | cg08360728 | 1 | *GPATCH3* | TSS200 | 1.06 | 0.78-1.45 | 0.7114 |  | Hypo-methylation |
|  | cg08908355 | 1 | *HIST2H2BE* | 1stExon | 1.13 | 0.82-1.55 | 0.4458 |  | Hypo-methylation |
|  | cg09649610 | 1 | *GNG4* | TSS1500 | 0.93 | 0.65-1.34 | 0.6953 |  | Hypo-methylation |
|  | cg12995941 | 1 | *NTRK1* | Body | 0.99 | 0.71-1.36 | 0.9294 |  | Hypo-methylation |
|  | cg14017991 | 1 | *CCDC21* | 1stExon | 1.07 | 0.8-1.43 | 0.6454 |  | Hypo-methylation |
|  | cg15991288 | 1 | *WDR47* | 5'UTR | 1.04 | 0.77-1.4 | 0.8132 |  | Hypo-methylation |
|  | cg16116363 | 1 | *SH3GLB1* | 1stExon | 1.06 | 0.78-1.44 | 0.6884 |  | Hypo-methylation |
|  | cg16632891 | 1 | *SSBP3* | TSS1500 | 1.03 | 0.78-1.35 | 0.8558 |  | Hypo-methylation |
|  | cg17424007 | 1 | *MYOG* | TSS1500 | 1.16 | 0.82-1.65 | 0.4011 |  | Hypo-methylation |
|  | cg18908499 | 1 | *C1orf150* | TSS1500 | 0.84 | 0.62-1.16 | 0.2909 |  | Hypo-methylation |
|  | cg19350340 | 1 | *ASPM* | TSS200 | 1.02 | 0.75-1.39 | 0.9187 |  | Hypo-methylation |
|  | cg19413110 | 1 | *POLR3GL* | 1stExon | 0.90 | 0.64-1.28 | 0.5632 |  | Hypo-methylation |
|  | cg20112035 | 1 | *TATDN3* | TSS200 | 1.07 | 0.78-1.45 | 0.6791 |  | Hypo-methylation |
|  | cg21379816 | 1 | *SRM* | Body | 1.06 | 0.77-1.47 | 0.7164 |  | Hypo-methylation |
|  | cg21680178 | 1 | *GPATCH3* | 1stExon | 0.89 | 0.63-1.26 | 0.5233 |  | Hypo-methylation |
|  | cg22226839 | 1 | *ATP2B4* | 5'UTR | 0.86 | 0.62-1.2 | 0.3824 |  | Hypo-methylation |
|  | cg22957381 | 1 | *KCND3* | TSS1500 | 0.98 | 0.74-1.28 | 0.8583 |  | Hypo-methylation |
|  | cg24305835 | 1 | *SDHB* | 5'UTR | 1.12 | 0.87-1.45 | 0.3805 |  | Hypo-methylation |
|  | cg03490115 | 2 | *INO80B* | TSS1500 | 0.90 | 0.65-1.25 | 0.5477 |  | Hypo-methylation |
|  | cg05091653 | 2 | *SP100* | Body | 0.83 | 0.59-1.15 | 0.262 |  | Hypo-methylation |
|  | cg07314414 | 2 | *SAP130* | TSS200 | 0.95 | 0.71-1.27 | 0.709 |  | Hypo-methylation |
|  | cg11736869 | 2 | *PEX13* | Body | 0.93 | 0.66-1.32 | 0.6935 |  | Hypo-methylation |
|  | cg13293721 | 2 | *RPIA* | TSS1500 | 0.97 | 0.69-1.38 | 0.8824 |  | Hypo-methylation |

Supplementary Table 1. Continued

| CpG sets | Probe | CHR | Gene | Region | OR^a^ | 95%CI | *P*-value | *Q*-value^b^ | Previously reported direction |
| --- | --- | --- | --- | --- | --- | --- | --- | --- | --- |
|  | cg19147570 | 2 | *C2orf86* | 1stExon | 0.99 | 0.72-1.35 | 0.9284 |  | Hypo-methylation |
|  | cg19658284 | 2 | *RBM43* | TSS200 | 1.09 | 0.85-1.38 | 0.4999 |  | Hypo-methylation |
|  | cg20732367 | 2 | *LRRFIP1* | Body | 1.01 | 0.72-1.43 | 0.9431 |  | Hypo-methylation |
|  | cg20959523 | 2 | *FIGN* | 1stExon | 1.11 | 0.78-1.59 | 0.5522 |  | Hypo-methylation |
|  | cg27074297 | 2 | *SOS1* | TSS1500 | 0.96 | 0.7-1.32 | 0.8192 |  | Hypo-methylation |
|  | cg00571634 | 3 | *WDR5B* | 1stExon | 0.95 | 0.71-1.28 | 0.7412 |  | Hypo-methylation |
|  | cg00841581 | 3 | *STAG1* | 5'UTR | 0.97 | 0.7-1.34 | 0.8495 |  | Hypo-methylation |
|  | cg01745657 | 3 | *PLCXD2* | TSS1500 | 1.07 | 0.81-1.41 | 0.6276 |  | Hypo-methylation |
|  | cg04428853 | 3 | *TMEM110* | 1stExon | 0.94 | 0.73-1.2 | 0.6032 |  | Hypo-methylation |
|  | cg05911610 | 3 | *MRPL3* | TSS200 | 1.06 | 0.8-1.39 | 0.6961 |  | Hypo-methylation |
|  | cg09134747 | 3 | *ATP2C1* | TSS1500 | 0.89 | 0.63-1.25 | 0.5031 |  | Hypo-methylation |
|  | cg10546523 | 3 | *SEMA3B* | Body | 1.08 | 0.81-1.43 | 0.6102 |  | Hypo-methylation |
|  | cg19642148 | 3 | *DHX30* | 5'UTR | 1.17 | 0.87-1.57 | 0.3091 |  | Hypo-methylation |
|  | cg19709625 | 3 | *PCOLCE2* | TSS200 | 0.88 | 0.7-1.12 | 0.3049 |  | Hypo-methylation |
|  | cg21246431 | 3 | *GK5* | TSS200 | 1.08 | 0.78-1.51 | 0.6338 |  | Hypo-methylation |
|  | cg25276849 | 3 | *TFRC* | 1stExon | 1.10 | 0.83-1.46 | 0.5145 |  | Hypo-methylation |
|  | cg25420398 | 3 | *RYK* | Body | 0.73 | 0.53-1.01 | 0.061 |  | Hypo-methylation |
|  | cg26640549 | 3 | *RAD18* | TSS200 | 0.83 | 0.59-1.16 | 0.2693 |  | Hypo-methylation |
|  | cg00886554 | 4 | *NMU* | TSS200 | 1.07 | 0.79-1.45 | 0.6517 |  | Hypo-methylation |
|  | cg03616357 | 4 | *MAP9* | 5'UTR | 1.13 | 0.82-1.57 | 0.4432 |  | Hypo-methylation |
|  | cg05028306 | 4 | *LAP3* | TSS1500 | 1.21 | 0.88-1.67 | 0.2407 |  | Hypo-methylation |
|  | cg05311623 | 4 | *ANXA5* | 5'UTR | 0.91 | 0.67-1.24 | 0.5626 |  | Hypo-methylation |
|  | cg09459044 | 4 | *PPA2* | Body | 1.16 | 0.86-1.56 | 0.3394 |  | Hypo-methylation |
|  | cg10133171 | 4 |  |  | 0.95 | 0.68-1.34 | 0.7752 |  | Hypo-methylation |
|  | cg12486486 | 4 | *YTHDC1* | Body | 1.04 | 0.77-1.39 | 0.807 |  | Hypo-methylation |
|  | cg13168683 | 4 | *JAKMIP1* | TSS200 | 1.24 | 0.93-1.64 | 0.1425 |  | Hypo-methylation |
|  | cg16420530 | 4 | *ING2* | Body | 1.21 | 0.92-1.59 | 0.174 |  | Hypo-methylation |
|  | cg22385477 | 4 | *CCDC110* | Body | 1.13 | 0.87-1.47 | 0.3487 |  | Hypo-methylation |
|  | cg24504843 | 4 | *CEP135* | 3'UTR | 0.95 | 0.71-1.26 | 0.698 |  | Hypo-methylation |
|  | cg25490411 | 4 | *MANBA* | 1stExon | 1.15 | 0.84-1.56 | 0.3909 |  | Hypo-methylation |
|  | cg25860314 | 4 | *SEC24D* | 1stExon | 0.89 | 0.64-1.22 | 0.4586 |  | Hypo-methylation |
|  | cg27406727 | 4 | *UGDH* | TSS1500 | 0.99 | 0.74-1.31 | 0.9375 |  | Hypo-methylation |
|  | cg01222684 | 5 | *TTC1* | TSS1500 | 0.90 | 0.65-1.26 | 0.5464 |  | Hypo-methylation |
|  | cg10237911 | 5 | *UTP15* | 5'UTR | 1.05 | 0.74-1.51 | 0.7723 |  | Hypo-methylation |
|  | cg10627136 | 5 | *HINT1* | Body | 1.01 | 0.73-1.41 | 0.9335 |  | Hypo-methylation |
|  | cg10681725 | 5 | *RPS23* | TSS1500 | 0.89 | 0.62-1.28 | 0.5384 |  | Hypo-methylation |
|  | cg13859478 | 5 | *CANX* | 5'UTR | 1.07 | 0.79-1.46 | 0.6531 |  | Hypo-methylation |
|  | cg22938407 | 5 | *CEP72* | TSS200 | 1.24 | 0.92-1.69 | 0.159 |  | Hypo-methylation |
|  | cg23054676 | 5 | *MTRR* | 1stExon | 0.90 | 0.65-1.25 | 0.5406 |  | Hypo-methylation |
|  | cg25428293 | 5 | *HMGXB3* | 5'UTR | 1.22 | 0.91-1.63 | 0.1907 |  | Hypo-methylation |
|  | cg00214855 | 6 | *CCDC90A* | Body | 0.92 | 0.68-1.26 | 0.6122 |  | Hypo-methylation |
|  | cg03338924 | 6 | *PHACTR1* | Body | 0.84 | 0.64-1.09 | 0.1818 |  | Hypo-methylation |
|  | cg03969906 | 6 | *HIST1H4A* | TSS200 | 0.99 | 0.75-1.3 | 0.9264 |  | Hypo-methylation |
|  | cg16041611 | 6 | *SRF* | 1stExon | 1.04 | 0.8-1.34 | 0.775 |  | Hypo-methylation |
|  | cg16208491 | 6 | *PRPF4B* | Body | 1.26 | 0.89-1.79 | 0.1883 |  | Hypo-methylation |
|  | cg19190714 | 6 | *GMPR* | 1stExon | 1.11 | 0.83-1.48 | 0.4768 |  | Hypo-methylation |
|  | cg19217872 | 6 | *ZNF187* | 5'UTR | 0.80 | 0.57-1.13 | 0.2131 |  | Hypo-methylation |

Supplementary Table 1. Continued

| CpG sets | Probe | CHR | Gene | Region | OR^a^ | 95%CI | *P*-value | *Q*-value^b^ | Previously reported direction |
| --- | --- | --- | --- | --- | --- | --- | --- | --- | --- |
|  | cg20235510 | 6 | *ZNF311* | Body | 0.82 | 0.57-1.17 | 0.2778 |  | Hypo-methylation |
|  | cg20640749 | 6 | *GLP1R* | 3'UTR | 0.88 | 0.61-1.25 | 0.4599 |  | Hypo-methylation |
|  | cg25438963 | 6 | *HIST1H3C* | 1stExon | 0.78 | 0.5-1.21 | 0.2622 |  | Hypo-methylation |
|  | cg03916490 | 7 | *C7orf50* | Body | 0.83 | 0.67-1.02 | 0.0804 |  | Hypo-methylation |
|  | cg03998173 | 7 | *RHEB* | TSS1500 | 1.25 | 0.94-1.68 | 0.124 |  | Hypo-methylation |
|  | cg07681084 | 7 | *LMBR1* | 1stExon | 0.84 | 0.64-1.11 | 0.2192 |  | Hypo-methylation |
|  | cg11997899 | 7 | *DLX5* | TSS200 | 1.16 | 0.83-1.63 | 0.3801 |  | Hypo-methylation |
|  | cg13456653 | 7 | *DNAJB6* | 5'UTR | 1.10 | 0.79-1.53 | 0.56 |  | Hypo-methylation |
|  | cg17885062 | 7 | *NRCAM* | 5'UTR | 1.20 | 0.89-1.62 | 0.2266 |  | Hypo-methylation |
|  | cg19861697 | 7 | *DMTF1* | 5'UTR | 0.89 | 0.64-1.23 | 0.4714 |  | Hypo-methylation |
|  | cg23219720 | 7 | *ZNF804B* | TSS200 | 1.03 | 0.77-1.37 | 0.854 |  | Hypo-methylation |
|  | cg01836096 | 8 | *C8ORFK29* | TSS200 | 1.01 | 0.76-1.35 | 0.9204 |  | Hypo-methylation |
|  | cg06269753 | 8 | *MSC* | Body | 0.82 | 0.6-1.11 | 0.1921 |  | Hypo-methylation |
|  | cg10913563 | 8 | *C8orf55* | 1stExon | 0.82 | 0.58-1.18 | 0.2948 |  | Hypo-methylation |
|  | cg14830952 | 8 | *E2F5* | TSS200 | 1.02 | 0.77-1.36 | 0.8829 |  | Hypo-methylation |
|  | cg15264255 | 8 | *NIPAL2* | TSS200 | 0.97 | 0.71-1.34 | 0.856 |  | Hypo-methylation |
|  | cg16007680 | 8 | *GLI4* | 5'UTR | 1.07 | 0.76-1.49 | 0.7008 |  | Hypo-methylation |
|  | cg16659470 | 8 | *RBM12B* | 3'UTR | 1.09 | 0.76-1.57 | 0.63 |  | Hypo-methylation |
|  | cg22783363 | 8 | *TNFRSF10D* | TSS200 | 1.03 | 0.75-1.42 | 0.8651 |  | Hypo-methylation |
|  | **cg00594118** | 9 | *COBRA1* | 1stExon | 1.31 | 1.02-1.68 | 0.0334 | 0.9848 | Hypo-methylation |
|  | cg08287471 | 9 | *NEK6* | 5'UTR | 0.94 | 0.71-1.25 | 0.6785 |  | Hypo-methylation |
|  | cg15428653 | 9 | *ABCA2* | TSS1500 | 1.13 | 0.8-1.61 | 0.4817 |  | Hypo-methylation |
|  | cg03417317 | 10 | *TMEM180* | TSS200 | 1.09 | 0.8-1.5 | 0.5759 |  | Hypo-methylation |
|  | cg10949322 | 10 | *CUL2* | 1stExon | 1.01 | 0.77-1.33 | 0.9466 |  | Hypo-methylation |
|  | **cg15212440** | 10 | *SFXN2* | 5'UTR | 1.60 | 1.14-2.24 | 0.0071 | 0.5845 | Hypo-methylation |
|  | **cg17028039** | 10 | *FGFR2* | 5'UTR | 1.51 | 1.12-2.03 | 0.0062 | 0.5845 | Hypo-methylation |
|  | cg18472228 | 10 | *BAMBI* | 1stExon | 1.11 | 0.82-1.51 | 0.4817 |  | Hypo-methylation |
|  | cg23087130 | 10 | *ABI1* | Body | 0.83 | 0.61-1.14 | 0.2504 |  | Hypo-methylation |
|  | cg23494338 | 10 |  |  | 1.07 | 0.82-1.41 | 0.6084 |  | Hypo-methylation |
|  | **cg23702568** | 10 | *WAPAL* | 5'UTR | 1.32 | 1.02-1.72 | 0.0368 | 0.9848 | Hypo-methylation |
|  | cg23724447 | 10 | *BUB3* | 5'UTR | 1.19 | 0.88-1.62 | 0.2592 |  | Hypo-methylation |
|  | cg00007981 | 11 | *PANX1* | 1stExon | 1.08 | 0.79-1.46 | 0.6306 |  | Hypo-methylation |
|  | cg02692952 | 11 | *TRAF6* | 1stExon | 1.05 | 0.78-1.41 | 0.7482 |  | Hypo-methylation |
|  | cg06044117 | 11 | *FAM76B* | TSS1500 | 0.96 | 0.73-1.27 | 0.7737 |  | Hypo-methylation |
|  | cg10970251 | 11 | *SLC25A22* | TSS1500 | 0.97 | 0.7-1.35 | 0.8573 |  | Hypo-methylation |
|  | cg18392783 | 11 | *PRKCDBP* | 1stExon | 1.13 | 0.81-1.57 | 0.4712 |  | Hypo-methylation |
|  | cg24247865 | 11 | *CHORDC1* | 5'UTR | 0.79 | 0.57-1.1 | 0.1683 |  | Hypo-methylation |
|  | cg24407308 | 11 | *DGKZ* | 1stExon | 1.02 | 0.74-1.43 | 0.8856 |  | Hypo-methylation |
|  | cg26606064 | 11 | *EI24* | TSS1500 | 1.12 | 0.82-1.53 | 0.4827 |  | Hypo-methylation |
|  | cg00136105 | 12 | *GNPTAB* | TSS1500 | 0.90 | 0.66-1.24 | 0.5235 |  | Hypo-methylation |
|  | cg01341572 | 12 | *HNF1A* | TSS200 | 0.81 | 0.62-1.07 | 0.1405 |  | Hypo-methylation |
|  | cg04798824 | 12 | *ANKS1B* | Body | 0.85 | 0.61-1.17 | 0.3119 |  | Hypo-methylation |
|  | cg05450995 | 12 | *BCL7A* | Body | 1.23 | 0.93-1.62 | 0.1397 |  | Hypo-methylation |
|  | cg05567435 | 12 | *METTL7B* | TSS1500 | 1.00 | 0.69-1.44 | 0.9937 |  | Hypo-methylation |
|  | cg08578305 | 12 | *KRAS* | TSS200 | 0.91 | 0.65-1.29 | 0.606 |  | Hypo-methylation |
|  | cg09259332 | 12 | *EEA1* | Body | 1.12 | 0.83-1.52 | 0.4484 |  | Hypo-methylation |
|  | cg17303299 | 12 | *ENO2* | TSS200 | 0.85 | 0.63-1.15 | 0.3019 |  | Hypo-methylation |

Supplementary Table 1. Continued

| CpG sets | Probe | CHR | Gene | Region | OR^a^ | 95%CI | *P*-value | *Q*-value^b^ | Previously reported direction |
| --- | --- | --- | --- | --- | --- | --- | --- | --- | --- |
|  | cg17824939 | 12 | *C12orf50* | 5'UTR | 0.90 | 0.67-1.22 | 0.4992 |  | Hypo-methylation |
|  | cg21226225 | 12 | *TXNRD1* | 1stExon | 1.04 | 0.77-1.41 | 0.7761 |  | Hypo-methylation |
|  | **cg21570220** | 12 | *SLC11A2* | 5'UTR | 1.45 | 1.09-1.93 | 0.0109 | 0.6741 | Hypo-methylation |
|  | cg00274399 | 13 | *FGF9* | 5'UTR | 0.75 | 0.55-1.03 | 0.0727 |  | Hypo-methylation |
|  | cg00756058 | 13 | *DZIP1* | 5'UTR | 1.01 | 0.77-1.33 | 0.9202 |  | Hypo-methylation |
|  | cg06531158 | 13 | *MIR548F5* | Body | 1.10 | 0.76-1.6 | 0.6067 |  | Hypo-methylation |
|  | cg00025138 | 14 | *MAP3K9* | TSS200 | 1.12 | 0.85-1.48 | 0.4315 |  | Hypo-methylation |
|  | cg01078871 | 14 | *FBXO33* | 1stExon | 1.01 | 0.76-1.33 | 0.9702 |  | Hypo-methylation |
|  | cg06588247 | 14 | *TRAF3* | 5'UTR | 1.15 | 0.96-1.36 | 0.126 |  | Hypo-methylation |
|  | cg13905388 | 14 | *CDCA4* | 5'UTR | 0.95 | 0.74-1.21 | 0.6631 |  | Hypo-methylation |
|  | cg15078479 | 14 | *PARP2* | 1stExon | 0.93 | 0.7-1.24 | 0.6385 |  | Hypo-methylation |
|  | cg16920242 | 14 | *TEP1* | TSS200 | 1.34 | 0.97-1.85 | 0.079 |  | Hypo-methylation |
|  | cg21171130 | 14 | *FAM158A* | 5'UTR | 1.12 | 0.87-1.45 | 0.3699 |  | Hypo-methylation |
|  | cg03643709 | 15 | *VPS18* | 1stExon | 0.79 | 0.56-1.12 | 0.1888 |  | Hypo-methylation |
|  | cg04187814 | 15 | *KIAA1199* | 5'UTR | 1.04 | 0.8-1.35 | 0.7692 |  | Hypo-methylation |
|  | cg07594478 | 15 | *ARIH1* | TSS200 | 1.13 | 0.86-1.5 | 0.3806 |  | Hypo-methylation |
|  | cg12237946 | 15 | *PGBD4* | 1stExon | 0.97 | 0.74-1.29 | 0.8553 |  | Hypo-methylation |
|  | cg12426141 | 15 | *TCF12* | 5'UTR | 1.18 | 0.91-1.52 | 0.2206 |  | Hypo-methylation |
|  | cg12460541 | 15 | *ITPKA* | TSS1500 | 1.31 | 0.95-1.82 | 0.1043 |  | Hypo-methylation |
|  | cg13140465 | 15 | *NIPA1* | Body | 0.93 | 0.64-1.35 | 0.6869 |  | Hypo-methylation |
|  | cg15025769 | 15 | *MYO9A* | 5'UTR | 0.92 | 0.68-1.23 | 0.5577 |  | Hypo-methylation |
|  | cg15523238 | 15 | *ATP10A* | TSS1500 | 0.90 | 0.68-1.19 | 0.4634 |  | Hypo-methylation |
|  | cg16940801 | 15 | *PKM2* | 1stExon | 1.32 | 0.99-1.76 | 0.0626 |  | Hypo-methylation |
|  | cg18766912 | 15 | *UBE3A* | 1stExon | 1.18 | 0.93-1.49 | 0.1787 |  | Hypo-methylation |
|  | cg00962707 | 16 |  |  | 0.90 | 0.67-1.2 | 0.4641 |  | Hypo-methylation |
|  | cg04979725 | 16 | *UNKL* | Body | 0.89 | 0.65-1.22 | 0.4698 |  | Hypo-methylation |
|  | cg05361373 | 16 | *PYCARD* | 1stExon | 1.06 | 0.77-1.45 | 0.7371 |  | Hypo-methylation |
|  | cg15645784 | 16 | *ROGDI* | TSS1500 | 1.05 | 0.76-1.45 | 0.7486 |  | Hypo-methylation |
|  | cg19216839 | 16 | *FANCA* | Body | 1.14 | 0.86-1.51 | 0.3608 |  | Hypo-methylation |
|  | cg23217005 | 16 | *MT1F* | TSS200 | 1.35 | 0.98-1.86 | 0.0688 |  | Hypo-methylation |
|  | cg24930915 | 16 | *RAB26* | Body | 1.26 | 0.93-1.7 | 0.1349 |  | Hypo-methylation |
|  | cg00126034 | 17 | *WNT3* | TSS200 | 0.99 | 0.73-1.33 | 0.9246 |  | Hypo-methylation |
|  | cg06307169 | 17 | *CASC3* | 5'UTR | 0.95 | 0.73-1.23 | 0.6887 |  | Hypo-methylation |
|  | cg07112154 | 17 | *MPP2* | 5'UTR | 0.91 | 0.63-1.31 | 0.6072 |  | Hypo-methylation |
|  | cg09907395 | 17 | *RNF213* | 5'UTR | 1.22 | 0.89-1.68 | 0.2121 |  | Hypo-methylation |
|  | cg18085206 | 17 | *CRK* | 1stExon | 0.80 | 0.59-1.09 | 0.1591 |  | Hypo-methylation |
|  | cg21000021 | 17 | *MYBBP1A* | TSS1500 | 0.97 | 0.7-1.36 | 0.8715 |  | Hypo-methylation |
|  | cg26813908 | 17 | *CCDC55* | TSS1500 | 0.89 | 0.64-1.24 | 0.4941 |  | Hypo-methylation |
|  | cg02366059 | 18 | *C18orf45* | Body | 0.94 | 0.68-1.31 | 0.7312 |  | Hypo-methylation |
|  | cg04510262 | 18 | *PTPN2* | 1stExon | 1.12 | 0.86-1.47 | 0.4085 |  | Hypo-methylation |
|  | cg18038361 | 18 | *TTR* | TSS1500 | 1.10 | 0.78-1.56 | 0.5938 |  | Hypo-methylation |
|  | cg18419020 | 18 | *C18orf56* | 3'UTR | 1.29 | 0.96-1.72 | 0.0909 |  | Hypo-methylation |
|  | cg26772788 | 18 |  |  | 1.03 | 0.74-1.44 | 0.8423 |  | Hypo-methylation |
|  | cg03430067 | 19 | *C19orf57* | TSS200 | 1.00 | 0.75-1.33 | 0.9925 |  | Hypo-methylation |
|  | cg07847428 | 19 | *02-Mar* | TSS200 | 0.86 | 0.64-1.15 | 0.3154 |  | Hypo-methylation |
|  | cg09231514 | 19 | *HSD11B1L* | 5'UTR | 0.84 | 0.61-1.17 | 0.3006 |  | Hypo-methylation |
|  | cg10262425 | 19 | *PGLS* | TSS200 | 1.18 | 0.87-1.62 | 0.2915 |  | Hypo-methylation |

Supplementary Table 1. Continued

| CpG sets | Probe | CHR | Gene | Region | OR^a^ | 95%CI | *P*-value | *Q*-value^b^ | Previously reported direction |
| --- | --- | --- | --- | --- | --- | --- | --- | --- | --- |
|  | cg13332130 | 19 | *RAB4B* | 5'UTR | 1.17 | 0.88-1.57 | 0.2862 |  | Hypo-methylation |
|  | cg17283268 | 19 | *POLRMT* | 1stExon | 1.03 | 0.78-1.37 | 0.8126 |  | Hypo-methylation |
|  | cg17378989 | 19 | *ERCC1* | 5'UTR | 0.93 | 0.67-1.28 | 0.6537 |  | Hypo-methylation |
|  | cg20643362 | 19 | *C19orf12* | 1stExon | 1.21 | 0.92-1.59 | 0.174 |  | Hypo-methylation |
|  | cg22517351 | 19 | *GLT25D1* | Body | 1.15 | 0.89-1.5 | 0.2928 |  | Hypo-methylation |
|  | cg23963136 | 19 | *CD320* | Body | 0.99 | 0.73-1.34 | 0.957 |  | Hypo-methylation |
|  | cg00340102 | 20 | *ERGIC3* | TSS1500 | 1.00 | 0.72-1.38 | 0.9796 |  | Hypo-methylation |
|  | cg01951308 | 20 | *KIF3B* | 1stExon | 0.97 | 0.7-1.34 | 0.8589 |  | Hypo-methylation |
|  | cg05343548 | 20 |  |  | 1.06 | 0.76-1.48 | 0.7468 |  | Hypo-methylation |
|  | cg07115304 | 20 | *TRIB3* | 5'UTR | 0.91 | 0.68-1.22 | 0.5333 |  | Hypo-methylation |
|  | cg09598225 | 20 | *GNAS* | 3'UTR | 1.24 | 0.88-1.74 | 0.2203 |  | Hypo-methylation |
|  | cg22084611 | 20 | *SULF2* | 1stExon | 1.13 | 0.87-1.45 | 0.361 |  | Hypo-methylation |
|  | **cg04454259** | 21 | *CBR3* | 1stExon | 1.43 | 1.1-1.85 | 0.0069 | 0.5845 | Hypo-methylation |
|  | cg13552650 | 21 | *MORC3* | TSS1500 | 0.98 | 0.69-1.39 | 0.9186 |  | Hypo-methylation |
|  | cg03475420 | 22 | *RTN4R* | 5'UTR | 1.18 | 0.89-1.55 | 0.2503 |  | Hypo-methylation |
|  | cg06405090 | 22 | *TTLL12* | Body | 0.87 | 0.63-1.2 | 0.3956 |  | Hypo-methylation |
|  | cg06534422 | 22 | *MIF* | 1stExon | 1.16 | 0.87-1.54 | 0.3158 |  | Hypo-methylation |
|  | cg11672225 | 22 | *RNF185* | 1stExon | 0.96 | 0.74-1.24 | 0.7306 |  | Hypo-methylation |
|  | cg13184872 | 22 | *PNPLA3* | 1stExon | 0.97 | 0.81-1.17 | 0.7865 |  | Hypo-methylation |
|  | cg15103426 | 22 | *CCDC117* | 1stExon | 0.92 | 0.65-1.3 | 0.6352 |  | Hypo-methylation |
|  | cg15879316 | 22 | *CELSR1* | TSS1500 | 0.79 | 0.57-1.11 | 0.1756 |  | Hypo-methylation |
|  | cg25094927 | 22 | *RANGAP1* | 1stExon | 1.17 | 0.88-1.56 | 0.2873 |  | Hypo-methylation |
|  | cg05455393 | X | *FHL1* | TSS1500 | 0.91 | 0.66-1.25 | 0.5566 |  | Hypo-methylation |
| 131-CpGs  (Xu et al., 2019) |  |  |  |  |  |  |  |  |  |
|  | cg03754165 | 2 | BCL11A | 1stExon | 1.22 | 0.94-1.58 | 0.1335 |  | Hyper-methylation |
|  | cg08172999 | 2 | C2orf69 | TSS1500 | 0.91 | 0.69-1.2 | 0.4907 |  | Hyper-methylation |
|  | cg12968518 | 2 | ACVR2A | TSS200 | 1.17 | 0.91-1.5 | 0.2345 |  | Hyper-methylation |
|  | cg22902939 | 2 | KCTD18 | Body | 1.18 | 0.85-1.63 | 0.3123 |  | Hyper-methylation |
|  | cg04104820 | 3 | WDR82 | 3'UTR | 0.86 | 0.6-1.24 | 0.4296 |  | Hyper-methylation |
|  | cg11630696 | 3 | ROBO1 | TSS1500 | 1.17 | 0.9-1.53 | 0.2371 |  | Hyper-methylation |
|  | cg25710815 | 4 | HERC6 | 1stExon | 1.03 | 0.79-1.33 | 0.8409 |  | Hyper-methylation |
|  | cg19977964 | 5 | C5orf35 | Body | 1.22 | 0.94-1.59 | 0.1335 |  | Hyper-methylation |
|  | cg05099288 | 6 | CD164 | Body | 0.85 | 0.62-1.16 | 0.2954 |  | Hyper-methylation |
|  | cg07439409 | 6 | MIR548H3 | Body | 1.03 | 0.67-1.58 | 0.8919 |  | Hyper-methylation |
|  | cg23819836 | 6 | DTNBP1 | 1stExon | 1.10 | 0.84-1.44 | 0.4908 |  | Hyper-methylation |
|  | cg06193393 | 7 | GATS | Body | 1.21 | 0.89-1.63 | 0.2221 |  | Hyper-methylation |
|  | cg01296653 | 9 | CDC37L1 | TSS1500 | 1.11 | 0.83-1.49 | 0.4791 |  | Hyper-methylation |
|  | cg26771998 | 10 | BMS1P4 | TSS200 | 1.25 | 0.87-1.78 | 0.2257 |  | Hyper-methylation |
|  | cg13544125 | 11 | PSMA1 | 5'UTR | 1.05 | 0.74-1.49 | 0.7852 |  | Hyper-methylation |
|  | cg14908986 | 11 | PPFIA1 | Body | 1.08 | 0.78-1.5 | 0.6319 |  | Hyper-methylation |
|  | cg21074594 | 11 |  |  | 0.98 | 0.72-1.33 | 0.889 |  | Hyper-methylation |
|  | **cg05099186** | 13 | LHFP | Body | 1.51 | 1.05-2.17 | 0.027 | 0.8844 | Hyper-methylation |
|  | cg08866608 | 13 |  |  | 0.94 | 0.72-1.23 | 0.6399 |  | Hyper-methylation |
|  | cg23173466 | 14 | MAX | Body | 0.86 | 0.64-1.17 | 0.3407 |  | Hyper-methylation |
|  | cg03117577 | 17 | CYTSB | Body | 1.28 | 0.93-1.75 | 0.1296 |  | Hyper-methylation |

Supplementary Table 1. Continued

| CpG sets | Probe | CHR | Gene | Region | OR^a^ | 95%CI | *P*-value | *Q*-value^b^ | Previously reported direction |
| --- | --- | --- | --- | --- | --- | --- | --- | --- | --- |
|  | cg23419170 | 18 |  |  | 1.33 | 0.96-1.82 | 0.0834 |  | Hyper-methylation |
|  | cg02679291 | 19 | GLTSCR2 | 1stExon | 1.12 | 0.85-1.48 | 0.4166 |  | Hyper-methylation |
|  | cg11433319 | 21 | CHODL | Body | 0.93 | 0.73-1.17 | 0.5197 |  | Hyper-methylation |
|  | cg00162673 | 1 | RNF11 | TSS1500 | 0.95 | 0.72-1.25 | 0.7189 |  | Hypo-methylation |
|  | cg02083528 | 1 | FAM73A | Body | 0.89 | 0.67-1.18 | 0.4093 |  | Hypo-methylation |
|  | **cg06698332** | 1 | S100A10 | TSS1500 | 0.70 | 0.52-0.93 | 0.016 | 0.8844 | Hypo-methylation |
|  | cg07031542 | 1 | PTPN14 | Body | 1.03 | 0.78-1.36 | 0.8405 |  | Hypo-methylation |
|  | cg09136695 | 1 | CEPT1 | Body | 1.05 | 0.77-1.43 | 0.7504 |  | Hypo-methylation |
|  | cg09664975 | 1 | PRKACB | TSS200 | 0.89 | 0.64-1.23 | 0.4664 |  | Hypo-methylation |
|  | cg11750112 | 1 | CACHD1 | Body | 0.85 | 0.65-1.12 | 0.2504 |  | Hypo-methylation |
|  | cg13865248 | 1 |  |  | 0.87 | 0.66-1.15 | 0.319 |  | Hypo-methylation |
|  | cg16008440 | 1 | OLFML2B | Body | 0.82 | 0.61-1.11 | 0.1955 |  | Hypo-methylation |
|  | cg21584883 | 1 | TMED5 | 1stExon | 0.90 | 0.67-1.22 | 0.5045 |  | Hypo-methylation |
|  | cg25768273 | 1 |  |  | 1.18 | 0.89-1.57 | 0.2531 |  | Hypo-methylation |
|  | cg26883190 | 1 |  |  | 0.94 | 0.74-1.2 | 0.6219 |  | Hypo-methylation |
|  | cg00008800 | 2 |  |  | 0.98 | 0.75-1.26 | 0.8491 |  | Hypo-methylation |
|  | cg01580574 | 2 |  |  | 0.88 | 0.66-1.17 | 0.3871 |  | Hypo-methylation |
|  | cg02540338 | 2 |  |  | 1.15 | 0.87-1.52 | 0.3381 |  | Hypo-methylation |
|  | cg03555299 | 2 |  |  | 0.96 | 0.71-1.3 | 0.7931 |  | Hypo-methylation |
|  | cg04819081 | 2 | ATAD2B | Body | 0.89 | 0.66-1.19 | 0.4198 |  | Hypo-methylation |
|  | cg05059169 | 2 | COQ10B | 3'UTR | 0.83 | 0.62-1.11 | 0.2101 |  | Hypo-methylation |
|  | **cg05999729** | 2 |  |  | 1.49 | 1.09-2.04 | 0.0113 | 0.8844 | Hypo-methylation |
|  | cg22727572 | 2 | LOC100132215 | Body | 1.13 | 0.87-1.48 | 0.3576 |  | Hypo-methylation |
|  | cg23562675 | 2 | C2orf76 | 5'UTR | 0.94 | 0.7-1.26 | 0.6617 |  | Hypo-methylation |
|  | cg24654185 | 2 | MRPL30 | 3'UTR | 0.99 | 0.75-1.33 | 0.9682 |  | Hypo-methylation |
|  | cg24690060 | 2 | LRRTM4 | Body | 1.17 | 0.85-1.62 | 0.3418 |  | Hypo-methylation |
|  | cg25783997 | 2 | ZNF804A | TSS200 | 0.88 | 0.63-1.23 | 0.4525 |  | Hypo-methylation |
|  | cg25995995 | 2 | NDUFA10 | Body | 0.92 | 0.69-1.23 | 0.5881 |  | Hypo-methylation |
|  | cg26569634 | 2 | NOL10 | Body | 0.90 | 0.68-1.18 | 0.4417 |  | Hypo-methylation |
|  | cg01551787 | 3 | GXYLT2 | Body | 0.79 | 0.59-1.06 | 0.1159 |  | Hypo-methylation |
|  | cg01954930 | 3 | LOC255025 | TSS200 | 0.85 | 0.64-1.12 | 0.2509 |  | Hypo-methylation |
|  | cg04872851 | 3 |  |  | 1.16 | 0.86-1.55 | 0.3349 |  | Hypo-methylation |
|  | cg10723575 | 3 | WWTR1 | TSS1500 | 0.99 | 0.73-1.34 | 0.9409 |  | Hypo-methylation |
|  | cg19385799 | 3 |  |  | 0.91 | 0.68-1.22 | 0.5242 |  | Hypo-methylation |
|  | cg21331107 | 3 | CMTM6 | Body | 1.00 | 0.74-1.35 | 0.9947 |  | Hypo-methylation |
|  | cg23535170 | 3 | USP13 | Body | 1.10 | 0.81-1.48 | 0.5441 |  | Hypo-methylation |
|  | cg06501333 | 4 |  |  | 1.03 | 0.77-1.38 | 0.8175 |  | Hypo-methylation |
|  | cg01021224 | 5 | AGXT2 | TSS1500 | 0.83 | 0.61-1.11 | 0.2099 |  | Hypo-methylation |
|  | cg01885839 | 5 |  |  | 1.10 | 0.83-1.45 | 0.5101 |  | Hypo-methylation |
|  | cg02456218 | 5 | BTNL9 | Body | 1.07 | 0.82-1.4 | 0.5988 |  | Hypo-methylation |
|  | cg10825350 | 5 | FSTL4 | Body | 1.02 | 0.77-1.35 | 0.8772 |  | Hypo-methylation |
|  | **cg19780570** | 5 |  |  | 0.74 | 0.56-0.96 | 0.0252 | 0.8844 | Hypo-methylation |
|  | cg06079966 | 6 |  |  | 1.09 | 0.78-1.51 | 0.6157 |  | Hypo-methylation |
|  | cg14840502 | 6 |  |  | 0.95 | 0.68-1.33 | 0.7861 |  | Hypo-methylation |
|  | cg19082069 | 6 | HIST1H1A | TSS1500 | 0.91 | 0.65-1.27 | 0.5913 |  | Hypo-methylation |
|  | cg19494811 | 6 | NUDT3 | Body | 1.02 | 0.75-1.39 | 0.9091 |  | Hypo-methylation |
|  | cg24152718 | 6 |  |  | 0.79 | 0.56-1.11 | 0.1756 |  | Hypo-methylation |

Supplementary Table 1. Continued

| CpG sets | Probe | CHR | Gene | Region | OR^a^ | 95%CI | *P*-value | *Q*-value^b^ | Previously reported direction |
| --- | --- | --- | --- | --- | --- | --- | --- | --- | --- |
|  | cg24310785 | 6 | QKI | Body | 0.82 | 0.59-1.14 | 0.2327 |  | Hypo-methylation |
|  | cg26526443 | 6 |  |  | 1.07 | 0.8-1.44 | 0.6408 |  | Hypo-methylation |
|  | cg06479434 | 7 | CALU | Body | 1.00 | 0.75-1.35 | 0.9757 |  | Hypo-methylation |
|  | cg10412943 | 7 | LMTK2 | Body | 0.96 | 0.69-1.34 | 0.7987 |  | Hypo-methylation |
|  | cg17345859 | 7 | ZNF12 | 3'UTR | 0.83 | 0.62-1.11 | 0.2061 |  | Hypo-methylation |
|  | cg20864326 | 7 | XRCC2 | Body | 1.13 | 0.84-1.51 | 0.4212 |  | Hypo-methylation |
|  | cg21921829 | 7 | ST7 | Body | 1.18 | 0.87-1.6 | 0.2932 |  | Hypo-methylation |
|  | cg22277672 | 7 | NCAPG2 | Body | 0.81 | 0.63-1.05 | 0.1133 |  | Hypo-methylation |
|  | cg24495177 | 7 | ZNF273 | TSS1500 | 0.97 | 0.74-1.27 | 0.8308 |  | Hypo-methylation |
|  | cg26203572 | 7 |  |  | 1.03 | 0.77-1.38 | 0.8394 |  | Hypo-methylation |
|  | cg12861602 | 8 | MATN2 | Body | 1.05 | 0.78-1.42 | 0.7506 |  | Hypo-methylation |
|  | cg12927915 | 8 | ARMC1 | 5'UTR | 0.99 | 0.73-1.34 | 0.9381 |  | Hypo-methylation |
|  | cg13155823 | 8 | TPD52 | Body | 0.99 | 0.73-1.34 | 0.9238 |  | Hypo-methylation |
|  | cg15328990 | 8 |  |  | 1.19 | 0.9-1.57 | 0.2264 |  | Hypo-methylation |
|  | cg17477493 | 8 |  |  | 1.10 | 0.81-1.5 | 0.5383 |  | Hypo-methylation |
|  | cg14569423 | 9 | DENND1A | Body | 1.03 | 0.77-1.38 | 0.853 |  | Hypo-methylation |
|  | cg21246531 | 9 | RECK | Body | 0.86 | 0.65-1.14 | 0.2902 |  | Hypo-methylation |
|  | cg01433914 | 10 | ADD3 | 5'UTR | 1.01 | 0.74-1.38 | 0.9398 |  | Hypo-methylation |
|  | cg03539267 | 10 |  |  | 1.15 | 0.85-1.56 | 0.3676 |  | Hypo-methylation |
|  | cg08320413 | 10 | MMP21 | 3'UTR | 1.14 | 0.86-1.52 | 0.3538 |  | Hypo-methylation |
|  | cg16719077 | 10 | ATP5C1 | 3'UTR | 0.94 | 0.68-1.31 | 0.7326 |  | Hypo-methylation |
|  | cg23260111 | 10 | ANUBL1 | Body | 0.91 | 0.67-1.22 | 0.5164 |  | Hypo-methylation |
|  | cg25248628 | 10 |  |  | 1.26 | 0.93-1.71 | 0.1407 |  | Hypo-methylation |
|  | cg26848011 | 10 | CHUK | TSS1500 | 0.85 | 0.65-1.1 | 0.2173 |  | Hypo-methylation |
|  | cg20006187 | 11 |  |  | 1.20 | 0.89-1.63 | 0.2254 |  | Hypo-methylation |
|  | cg20667822 | 11 | PTPRJ | Body | 0.99 | 0.76-1.3 | 0.9624 |  | Hypo-methylation |
|  | cg21107103 | 11 | SPTY2D1 | 3'UTR | 0.97 | 0.73-1.3 | 0.8427 |  | Hypo-methylation |
|  | cg22152887 | 11 | FCHSD2 | Body | 0.89 | 0.67-1.18 | 0.4019 |  | Hypo-methylation |
|  | cg11496226 | 12 | TMEM132D | Body | 0.82 | 0.62-1.1 | 0.188 |  | Hypo-methylation |
|  | cg13425650 | 12 | CRADD | Body | 1.11 | 0.8-1.53 | 0.5266 |  | Hypo-methylation |
|  | cg20251156 | 12 | OR6C65 | TSS1500 | 1.13 | 0.81-1.56 | 0.4725 |  | Hypo-methylation |
|  | cg22473961 | 12 | CD63 | 3'UTR | 1.14 | 0.85-1.54 | 0.3785 |  | Hypo-methylation |
|  | cg23828765 | 12 | NAV3 | Body | 1.17 | 0.87-1.59 | 0.3038 |  | Hypo-methylation |
|  | cg25282652 | 12 |  |  | 0.83 | 0.62-1.1 | 0.1955 |  | Hypo-methylation |
|  | **cg01075555** | 13 |  |  | 1.39 | 1.02-1.9 | 0.04 | 0.8844 | Hypo-methylation |
|  | cg04139515 | 13 | SLAIN1 | 1stExon | 0.97 | 0.71-1.32 | 0.831 |  | Hypo-methylation |
|  | cg16574347 | 13 | SLAIN1 | Body | 1.01 | 0.75-1.37 | 0.944 |  | Hypo-methylation |
|  | cg18462916 | 13 | CUL4A | 3'UTR | 0.82 | 0.61-1.1 | 0.1892 |  | Hypo-methylation |
|  | cg25399333 | 13 |  |  | 1.17 | 0.86-1.6 | 0.3237 |  | Hypo-methylation |
|  | cg05180258 | 14 | NUMB | 5'UTR | 1.11 | 0.82-1.49 | 0.5101 |  | Hypo-methylation |
|  | cg11720863 | 14 | PSME1 | TSS1500 | 1.21 | 0.92-1.6 | 0.1752 |  | Hypo-methylation |
|  | cg13979573 | 14 |  |  | 0.97 | 0.7-1.34 | 0.8502 |  | Hypo-methylation |
|  | cg02539984 | 15 | ADAM10 | Body | 1.32 | 0.96-1.8 | 0.0826 |  | Hypo-methylation |
|  | cg04258046 | 15 | RPS27L | 3'UTR | 0.88 | 0.65-1.19 | 0.3959 |  | Hypo-methylation |
|  | cg04474209 | 15 | MYO5C | 3'UTR | 1.00 | 0.73-1.38 | 0.9794 |  | Hypo-methylation |
|  | cg10542300 | 15 | MYEF2 | Body | 1.07 | 0.78-1.47 | 0.6871 |  | Hypo-methylation |
|  | cg13402936 | 15 |  |  | 1.02 | 0.77-1.35 | 0.8774 |  | Hypo-methylation |

Supplementary Table 1. Continued

| CpG sets | Probe | CHR | Gene | Region | OR^a^ | 95%CI | *P*-value | *Q*-value^b^ | Previously reported direction |
| --- | --- | --- | --- | --- | --- | --- | --- | --- | --- |
|  | cg22731164 | 15 | GPR176 | Body | 1.12 | 0.8-1.56 | 0.5229 |  | Hypo-methylation |
|  | cg25217313 | 15 | NUSAP1 | TSS200 | 1.08 | 0.81-1.44 | 0.5881 |  | Hypo-methylation |
|  | cg16743903 | 16 | SPG7 | Body | 1.13 | 0.88-1.46 | 0.3367 |  | Hypo-methylation |
|  | cg01691987 | 17 | CHD3 | Body | 0.91 | 0.67-1.25 | 0.5789 |  | Hypo-methylation |
|  | cg02704946 | 17 | KIAA0753 | Body | 1.05 | 0.78-1.41 | 0.7488 |  | Hypo-methylation |
|  | cg04256065 | 17 | TNRC6C | 3'UTR | 0.86 | 0.66-1.12 | 0.2737 |  | Hypo-methylation |
|  | cg09628499 | 17 |  |  | 0.98 | 0.69-1.39 | 0.9098 |  | Hypo-methylation |
|  | cg27657459 | 17 |  |  | 1.17 | 0.85-1.62 | 0.3398 |  | Hypo-methylation |
|  | cg05692899 | 18 |  |  | 1.52 | 0.95-2.41 | 0.0788 |  | Hypo-methylation |
|  | cg12361352 | 18 | ASXL3 | 5'UTR | 0.85 | 0.63-1.15 | 0.2934 |  | Hypo-methylation |
|  | cg16954236 | 18 | RAB12 | 3'UTR | 1.01 | 0.73-1.39 | 0.9454 |  | Hypo-methylation |
|  | cg06632759 | 19 | ZNF585B | TSS1500 | 0.92 | 0.69-1.23 | 0.5949 |  | Hypo-methylation |
|  | cg10812466 | 19 |  |  | 0.94 | 0.69-1.29 | 0.6936 |  | Hypo-methylation |
|  | cg18569070 | 19 | CPAMD8 | Body | 0.90 | 0.69-1.17 | 0.4284 |  | Hypo-methylation |
|  | cg25325053 | 20 | CDH26 | TSS1500 | 1.11 | 0.82-1.51 | 0.4872 |  | Hypo-methylation |
|  | cg01162841 | X | SH3KBP1 | TSS1500 | 1.01 | 0.77-1.32 | 0.9654 |  | Hypo-methylation |

^a^Odds ratio from unconditional logistic regression of the risk of breast cancer on β-value (per 1 s.d.) and model adjusted for age, batch effect, and leukocyte composition.

^b^Q-value are p-values adjusted for multiple testing according to the Benjamini-Hochberg method

Bold printed CpGs are sites showing a significant association with BC risk (P-value < 0.05) when multiple testing is not adjusted for

Abbreviation: CHR: chromosome; CI: confidence Interval; MRS: methylation risk score; OR: odds ratio; TSS: transcriptional start site; 3’UTR: the three prime untranslated region; 5’UTR: the 5’ untranslated region.

Supplement Table 2. SNPs used for genetic risk score construction

| 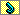SNP | Risk Allele | Chromosome | Position | Effect* |
| --- | --- | --- | --- | --- |
| rs11117758 | G | 1 | 217220574 | 0.0440 |
| rs11118563 | T | 1 | 220671050 | 0.0418 |
| rs11268668 | T | 1 | 204502514 | 0.0321 |
| rs114282204 | C | 1 | 41389220 | 0.1550 |
| rs12091730 | A | 1 | 155556971 | 0.0499 |
| rs12406858 | C | 1 | 118141492 | 0.0452 |
| rs139315904 | CA | 1 | 168171052 | 0.0680 |
| rs143384623 | C | 1 | 145604302 | 0.0399 |
| rs144105764 | T | 1 | 46670206 | 0.0447 |
| rs17426269 | A | 1 | 88156923 | 0.0494 |
| rs2785646 | G | 1 | 208076291 | 0.0366 |
| rs2992756 | T | 1 | 18807339 | 0.0564 |
| rs35383942 | T | 1 | 201437832 | 0.0917 |
| rs4233486 | T | 1 | 41380440 | 0.0426 |
| rs612683 | T | 1 | 100880328 | 0.0373 |
| rs616488 | A | 1 | 10566215 | 0.0586 |
| rs637868 | C | 1 | 120257110 | 0.0385 |
| **rs6656241** | T | 1 | 51464645 | 0.0374 |
| rs6686987 | C | 1 | 202184600 | 0.0065 |
| rs707475 | G | 1 | 7917076 | 0.0409 |
| rs72755295 | G | 1 | 242034263 | 0.1428 |
| rs7513707 | A | 1 | 114445880 | 0.0621 |
| rs7514172 | A | 1 | 203770448 | 0.0498 |
| rs10164550 | G | 2 | 121159205 | 0.0440 |
| rs10179592 | C | 2 | 121246568 | 0.0992 |
| rs10197246 | T | 2 | 202204741 | 0.0492 |
| rs1036759 | C | 2 | 88358825 | 0.0473 |
| rs12472404 | G | 2 | 29179452 | 0.0066 |
| rs1550622 | G | 2 | 174212910 | 0.0593 |
| rs17726078 | C | 2 | 172974566 | 0.0473 |
| rs2356656 | T | 2 | 192381934 | 0.0316 |
| **rs3791976** | A | 2 | 218714851 | 0.0431 |
| rs4322799 | T | 2 | 29615233 | 0.0427 |
| rs4676356 | C | 2 | 241388857 | 0.1232 |
| rs6725517 | A | 2 | 25129473 | 0.0427 |
| rs6743383 | T | 2 | 19315675 | 0.0331 |
| rs6746250 | A | 2 | 121058254 | 0.0334 |
| rs6756513 | G | 2 | 70172587 | 0.0412 |
| rs78425380 | C | 2 | 10138983 | 0.0603 |
| rs112476261 | C | 3 | 29294845 | 0.1281 |
| rs138866686 | A | 3 | 55970777 | 0.1195 |
| rs17838698 | T | 3 | 30684907 | 0.0592 |
| rs2886671 | C | 3 | 59373745 | 0.0394 |
| rs376397524 | C | 3 | 141112859 | 0.0551 |
| rs552647 | A | 3 | 27353716 | 0.0748 |
| rs56387622 | T | 3 | 46888198 | 0.0806 |
| rs58058861 | A | 3 | 172285237 | 0.0422 |
| rs62255657 | G | 3 | 27388664 | 0.0502 |
| rs639355 | G | 3 | 99403877 | 0.0376 |
| rs6762558 | G | 3 | 4742251 | 0.0616 |

Supplement Table 2. Continued

| 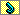SNP | Risk Allele | Chromosome | Position | Effect* |
| --- | --- | --- | --- | --- |
| rs9825432 | T | 3 | 71620370 | 0.0374 |
| rs9882792 | C | 3 | 189774456 | 0.0478 |
| rs10012017 | T | 4 | 38784633 | 0.0489 |
| rs138786872 | C | 4 | 151218296 | 0.0388 |
| rs17014016 | A | 4 | 89240476 | 0.0352 |
| rs28436676 | G | 4 | 175842495 | 0.0898 |
| rs532161833 | TAA | 4 | 84370124 | 0.0464 |
| rs56039025 | C | 4 | 143467195 | 0.0569 |
| rs62331150 | T | 4 | 106069013 | 0.0471 |
| rs62334414 | A | 4 | 175847436 | 0.0348 |
| rs10069690 | T | 5 | 1279790 | 0.0617 |
| rs10074269 | C | 5 | 169591460 | 0.0412 |
| rs10941679 | G | 5 | 44706498 | 0.0497 |
| rs116095464 | C | 5 | 345109 | 0.0840 |
| rs11949391 | T | 5 | 56045081 | 0.0564 |
| rs138335056 | G | 5 | 44508264 | 0.1177 |
| rs1428387 | T | 5 | 122705244 | 0.0944 |
| rs1432679 | C | 5 | 158244083 | 0.0677 |
| rs144028731 | GT | 5 | 77155397 | 0.0408 |
| rs146817970 | TA | 5 | 81512947 | 0.0598 |
| rs157557 | T | 5 | 73234583 | 0.0363 |
| rs16886165 | G | 5 | 56023083 | 0.1366 |
| rs17157372 | G | 5 | 104300273 | 0.0487 |
| rs17343002 | G | 5 | 44853593 | 0.0336 |
| rs17611291 | G | 5 | 16231194 | 0.0426 |
| rs187108781 | A | 5 | 44619502 | 0.1101 |
| rs3010266 | G | 5 | 71965007 | 0.0410 |
| rs3215401 | A | 5 | 1296255 | 0.0549 |
| rs332529 | G | 5 | 90789470 | 0.0564 |
| rs335160 | C | 5 | 122478676 | 0.0386 |
| rs34525310 | GA | 5 | 79180995 | 0.0328 |
| rs35130031 | T | 5 | 32579616 | 0.0363 |
| rs4613718 | T | 5 | 44649944 | 0.0492 |
| rs4866496 | A | 5 | 2777029 | 0.0391 |
| rs553874618 | C | 5 | 55662540 | 0.0458 |
| rs62329727 | C | 5 | 1353077 | 0.1552 |
| rs6596100 | C | 5 | 132407058 | 0.0388 |
| rs6860806 | G | 5 | 131640536 | 0.0392 |
| rs6864691 | A | 5 | 173358154 | 0.0365 |
| rs76250845 | T | 5 | 56042972 | 0.0865 |
| rs889310 | T | 5 | 55965167 | 0.0394 |
| rs111342015 | G | 6 | 43227141 | 0.0640 |
| rs12211970 | A | 6 | 18783140 | 0.0326 |
| rs146519950 | C | 6 | 85912194 | 0.0762 |
| rs2121348 | T | 6 | 149595505 | 0.0476 |
| rs34196306 | G | 6 | 27425644 | 0.0737 |
| rs418053 | G | 6 | 13713366 | 0.0553 |
| rs55941023 | CT | 6 | 130341728 | 0.0472 |
| rs60954078 | G | 6 | 151955914 | 0.1449 |
| rs6904031 | T | 6 | 152055978 | 0.0740 |

Supplement Table 2. Continued

| 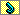SNP | Risk Allele | Chromosome | Position | Effect* |
| --- | --- | --- | --- | --- |
| rs6913578 | C | 6 | 151949806 | 0.0703 |
| rs6940159 | C | 6 | 170332621 | 0.0373 |
| rs73754909 | C | 6 | 87803819 | 0.0383 |
| rs851984 | A | 6 | 152023191 | 0.0626 |
| rs910416 | T | 6 | 152432902 | 0.0649 |
| rs9358466 | T | 6 | 21923810 | 0.0321 |
| rs111963714 | G | 7 | 99948655 | 0.0420 |
| rs13244925 | A | 7 | 55192256 | 0.0349 |
| rs17268829 | C | 7 | 94113799 | 0.0449 |
| rs289997 | C | 7 | 25569548 | 0.0486 |
| rs4439053 | G | 7 | 98005235 | 0.0467 |
| rs71559437 | G | 7 | 101552440 | 0.0568 |
| rs74765302 | G | 7 | 28869017 | 0.0572 |
| rs7800548 | C | 7 | 102481842 | 0.0418 |
| rs7971 | A | 7 | 21940960 | 0.0467 |
| rs10096351 | G | 8 | 128372172 | 0.0597 |
| rs1016578 | A | 8 | 129199566 | 0.0615 |
| rs1028016 | A | 8 | 23447496 | 0.0389 |
| rs12541094 | A | 8 | 124571581 | 0.0340 |
| rs12546444 | A | 8 | 106358620 | 0.0745 |
| rs12550713 | G | 8 | 128370949 | 0.0642 |
| rs13267382 | A | 8 | 117209548 | 0.0417 |
| rs13365225 | A | 8 | 36858483 | 0.0760 |
| rs1511243 | G | 8 | 76230943 | 0.0755 |
| rs1533366 | G | 8 | 76378165 | 0.0391 |
| **rs3008281** | C | 8 | 172477 | 0.0477 |
| rs310295 | A | 8 | 23663653 | 0.0335 |
| rs35542655 | C | 8 | 124563705 | 0.0477 |
| rs62517052 | C | 8 | 102483100 | 0.0593 |
| rs62526620 | G | 8 | 120862186 | 0.0527 |
| rs72658084 | T | 8 | 76333056 | 0.1129 |
| rs7830152 | A | 8 | 143669254 | 0.0346 |
| rs7842619 | G | 8 | 124739913 | 0.0466 |
| rs9693444 | A | 8 | 29509616 | 0.0601 |
| rs10120432 | C | 9 | 98362587 | 0.0576 |
| rs10816625 | G | 9 | 110837073 | 0.1158 |
| rs10975870 | G | 9 | 6880263 | 0.0348 |
| rs13294895 | T | 9 | 110837176 | 0.0653 |
| rs17694493 | G | 9 | 22041998 | 0.0289 |
| rs1895062 | A | 9 | 119313486 | 0.0462 |
| rs3861871 | A | 9 | 129424719 | 0.0382 |
| rs4880038 | C | 9 | 36928288 | 0.0249 |
| rs539723051 | C | 9 | 21964882 | 0.0550 |
| rs550057 | T | 9 | 136146597 | 0.0400 |
| rs60037937 | T | 9 | 110303808 | 0.0797 |
| rs630965 | T | 9 | 110885479 | 0.0877 |
| rs665889 | C | 9 | 87782211 | 0.0361 |
| rs7848334 | T | 9 | 110849525 | 0.0153 |
| rs10796139 | A | 10 | 13892298 | 0.0371 |
| rs10885405 | T | 10 | 114777670 | 0.0472 |

Supplement Table 2. Continued

| 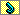SNP | Risk Allele | Chromosome | Position | Effect* |
| --- | --- | --- | --- | --- |
| rs10995201 | A | 10 | 64299890 | 0.1345 |
| rs12250948 | T | 10 | 115128491 | 0.0592 |
| rs140936696 | CAA | 10 | 95292187 | 0.0512 |
| rs35054928 | GC | 10 | 123340431 | 0.2408 |
| rs45631563 | A | 10 | 123349324 | 0.2609 |
| rs45631580 | G | 10 | 123340107 | 0.1508 |
| rs4980029 | G | 10 | 80886726 | 0.0762 |
| rs542275778 | A | 10 | 22477776 | 0.1687 |
| rs55910451 | G | 10 | 5794652 | 0.0470 |
| rs6479868 | T | 10 | 64819996 | 0.0472 |
| rs7072776 | A | 10 | 22032942 | 0.0580 |
| rs719338 | G | 10 | 80851257 | 0.0805 |
| rs9421410 | G | 10 | 123095209 | 0.0538 |
| rs10832963 | G | 11 | 18664241 | 0.0461 |
| rs10838267 | A | 11 | 44368892 | 0.0374 |
| rs10896047 | G | 11 | 65572431 | 0.0347 |
| rs11822830 | G | 11 | 129461016 | 0.0453 |
| rs12287832 | A | 11 | 65553492 | 0.0425 |
| rs199504893 | C | 11 | 108267402 | 0.0022 |
| rs35039974 | A | 11 | 69328130 | 0.0423 |
| rs4472923 | C | 11 | 42844441 | 0.0336 |
| rs4980386 | C | 11 | 1895708 | 0.0762 |
| rs610437 | T | 11 | 111696440 | 0.0396 |
| rs625145 | A | 11 | 116727936 | 0.0423 |
| rs6597981 | G | 11 | 803017 | 0.0457 |
| rs661204 | A | 11 | 69330983 | 0.1022 |
| rs7121616 | A | 11 | 122966626 | 0.0383 |
| rs7125780 | G | 11 | 103614438 | 0.0147 |
| rs7394715 | T | 11 | 433617 | 0.0437 |
| rs77047825 | C | 11 | 46318032 | 0.0748 |
| rs78540526 | T | 11 | 69331418 | 0.1782 |
| rs7939702 | T | 11 | 129243417 | 0.0543 |
| rs1027113 | A | 12 | 29140260 | 0.0647 |
| rs1061657 | C | 12 | 115108136 | 0.0465 |
| rs10862899 | T | 12 | 85004551 | 0.0348 |
| rs11049431 | C | 12 | 28347382 | 0.0521 |
| rs11065822 | G | 12 | 111600134 | 0.0442 |
| rs11067551 | A | 12 | 115796577 | 0.0428 |
| rs111622698 | GA | 12 | 83064195 | 0.0671 |
| rs12422552 | C | 12 | 14413931 | 0.0484 |
| rs17356907 | A | 12 | 96027759 | 0.0867 |
| rs206966 | T | 12 | 120832146 | 0.0516 |
| rs2454399 | T | 12 | 115835836 | 0.0813 |
| rs2870876 | T | 12 | 70798355 | 0.0469 |
| rs7132703 | T | 12 | 103097887 | 0.0546 |
| rs7297051 | C | 12 | 28174817 | 0.0856 |
| rs788458 | C | 12 | 28149568 | 0.0620 |
| rs797736 | G | 12 | 293626 | 0.0401 |
| rs11571833 | T | 13 | 32972626 | 0.2687 |
| rs12870942 | C | 13 | 73806982 | 0.0345 |

Supplement Table 2. Continued

| 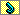SNP | Risk Allele | Chromosome | Position | Effect* |
| --- | --- | --- | --- | --- |
| rs2181965 | G | 13 | 73960952 | 0.0399 |
| rs56404467 | A | 13 | 32839990 | 0.0424 |
| rs9315973 | G | 13 | 43501356 | 0.0517 |
| rs11341843 | T | 14 | 91751788 | 0.0380 |
| rs11624333 | T | 14 | 68979835 | 0.0911 |
| rs2253012 | T | 14 | 37228504 | 0.0390 |
| rs2588809 | T | 14 | 68660428 | 0.0474 |
| rs34914085 | C | 14 | 37128564 | 0.0733 |
| rs4983544 | G | 14 | 105213978 | 0.0399 |
| rs78440108 | C | 14 | 93070286 | 0.0577 |
| rs941764 | G | 14 | 91841069 | 0.0513 |
| rs144767203 | A | 15 | 100905819 | 0.0608 |
| rs187010898 | C | 15 | 46680811 | 0.1973 |
| rs2290202 | G | 15 | 91512267 | 0.0589 |
| **rs28489579** | G | 15 | 50686778 | 0.0417 |
| rs35874463 | G | 15 | 67457698 | 0.0782 |
| rs8035987 | T | 15 | 75750383 | 0.0413 |
| rs8042593 | G | 15 | 66630569 | 0.0369 |
| rs11076805 | C | 16 | 4106788 | 0.0300 |
| rs12449271 | T | 16 | 87086492 | 0.0469 |
| rs12709163 | G | 16 | 6963972 | 0.0354 |
| rs28539243 | A | 16 | 54682064 | 0.0477 |
| rs34872983 | G | 16 | 10706580 | 0.0740 |
| rs35668161 | A | 16 | 52538825 | 0.1147 |
| rs4784227 | T | 16 | 52599188 | 0.1070 |
| rs55872725 | C | 16 | 53809123 | 0.0704 |
| rs6499648 | C | 16 | 53861139 | 0.0338 |
| rs7184573 | G | 16 | 53861592 | 0.0337 |
| rs7500067 | G | 16 | 80648296 | 0.0839 |
| rs75753503 | T | 16 | 23007047 | 0.1218 |
| rs9931038 | T | 16 | 85145977 | 0.0211 |
| rs11296 | C | 17 | 40127060 | 0.0174 |
| rs149370081 | A | 17 | 40744470 | 0.2017 |
| rs150537328 | C | 17 | 39251123 | 0.0799 |
| rs17881320 | G | 17 | 40485239 | 0.0571 |
| rs2787486 | A | 17 | 53209774 | 0.0793 |
| rs545502941 | CT | 17 | 43212339 | 0.0438 |
| rs745570 | A | 17 | 77781725 | 0.0401 |
| rs79461387 | G | 17 | 29168077 | 0.0568 |
| rs1111207 | C | 18 | 24125857 | 0.0346 |
| rs11665269 | C | 18 | 20634253 | 0.0415 |
| rs16976596 | C | 18 | 11696613 | 0.0381 |
| rs35369219 | AT | 18 | 24518050 | 0.0599 |
| **rs521667** | C | 18 | 24338479 | 0.0455 |
| rs8092192 | G | 18 | 25407513 | 0.0399 |
| rs9952980 | T | 18 | 42888797 | 0.0542 |
| rs9954058 | G | 18 | 42411803 | 0.0877 |
| rs10164323 | C | 19 | 18569492 | 0.0719 |
| rs1172821 | C | 19 | 55816678 | 0.0359 |
| rs117922601 | T | 19 | 13249921 | 0.0956 |

Supplement Table 2. Continued

| 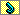SNP | Risk Allele | Chromosome | Position | Effect* |
| --- | --- | --- | --- | --- |
| rs140702307 | CGGGCG | 19 | 19517054 | 0.0437 |
| rs4399645 | T | 19 | 46166073 | 0.0360 |
| rs56069439 | A | 19 | 17393925 | 0.0378 |
| rs56681946 | C | 19 | 44283031 | 0.0619 |
| rs1154723 | C | 20 | 11379842 | 0.0844 |
| rs13039563 | A | 20 | 52296849 | 0.0440 |
| rs16991615 | A | 20 | 5948227 | 0.0760 |
| **rs965352** | C | 20 | 41611322 | 0.0315 |
| rs2403907 | C | 21 | 16574455 | 0.0707 |
| rs2822999 | G | 21 | 16364756 | 0.0646 |
| rs2823130 | G | 21 | 16566350 | 0.0595 |
| rs112855987 | G | 22 | 45319953 | 0.0134 |
| rs132289 | A | 22 | 29551872 | 0.1716 |
| rs17879961 | G | 22 | 29121087 | 0.1839 |
| rs34134147 | T | 22 | 29203724 | 0.1405 |
| rs5997390 | A | 22 | 29135543 | 0.0654 |
| rs66987842 | C | 22 | 40904707 | 0.1148 |
| rs9611990 | C | 22 | 43433100 | 0.0600 |
| rs9798754 | C | 22 | 19766137 | 0.0367 |

* effect refers to risk allele

Bold printed SNPs are surrogate SNPs.

Supplementary Table 3. Environmental risk scores from previous studies and availability/modification of related variables in ESTHER

| Study  (Country) | Variables, score points and categories | Information on variable availability/modification in ESTHER |
| --- | --- | --- |
| Gail, 1989  (USA) (Gail et al., 1989) | Score= -0.74948 + 0.09401*(AGEMEN) + 0.52926*(NBIOPS) + 0.21683* (AGEFLB) + 0.95830* (NUMREL_BR) + 0.01081*(AGE: code 1 if age ≥50, code 0 if age <50) – 0.28804 *(NBIOPS*(AGE: code 1 if age ≥50, code 0 if age <50)) - 0.19081*(AGEFLB*NUMREL_BR) | “Number of previous breast biopsies” was not available, hence excluded. |
| Novotny, 2006  (Czech) (Novotny et al., 2006) | MODEL I: Score=-0.0798-0.0969*(AGEMEN) + 0.2449*(NBIOPS) – 0.1129*(AGEFLB) + 0.4344*(NUMREL_BR) + 0.1110*(AGE: code 1 if age ≥50, code 0 if age<50) – 0.0057*(NBIOPS* (AGE: code 1 if age ≥50, code 0 if age <50)) – 0.1293*(AGEFLB*NUMREL_BR)  MODEL II: Score= -0.7145 - 0.1141*(AGEMEN) + 0.3197*(NBIOPS) - 0.1563*(AGEFLB) + 0.2867*(NUMREL_BR) – 0.0124*( AGE: code 1 if age ≥50, code 0 if age <50) – 0.0590*(NBIOPS* (AGE: code 1 if age ≥50, code 0 if age <50)) – 0.0879*(AGEFLB* NUMREL_BR)+0.1678*(NUMREL_Ca) + 0.5177*( BR_INFLAM: code 1 if yes, code 0 if no) + 0.0549*(BMI) – 0.2573*(Parity) | “Number of previous breast biopsies” and “Breast inflammation” were not available, hence excluded. |
| Park, 2013  (South Korea) (Park et al., 2013) | Score= = -0.6729 + 0.6960*( Fami_BC: code 1 if yes, code 0 if no) + 0.8755*( AGEMEN: code 1 if <13 years, code 0 if others) + 0.4244*( AGEMEN: code 1 if 13-16 years, code 0 if others) + 0.9154*(age at menopause: code 1 if premenopausal, code 0 if others) + 0.2954*(age at menopause: code 1 if 45-49 years, code 0 if others) + 0.3048*(age at menopause: code 1 if 50-54 years, code 0 if others) + 0.4794*(age at menopause: code 1 if ≥ 55 years old, code 0 if others) + 0.6287*(Parity: code 1 if nullipara, code 0 if para) + 0.1474*(BMI: code 1 if 25.0–29.9, code 0 if others) + 0.8239*(BMI: code 1 if ≥30, code 0 if <30) + 0.4175*(OC: code 1 if once a week or more, code 0 if less than once a week) + 0.6115*(PA: code 1 if <once/week, code 0 if ≥once/week) | All variables available and included. |

Supplementary Table 3. (Continued)

| Study  (Country) | Variables, score points and categories | Information on variable availability/modification in ESTHER |
| --- | --- | --- |
| Wang, 2016  (China) (Wang et al., 2016) | Premenopausal women  Score= 0.367*(Age) + 0.137*(Parity) + 0.855*(NUMREL_BR) + 0.631*(Alcohol Consumption) + 0.264*(Light at night: code 1 if dark, code 2 if few light, code 3 if little bright) – 0.256*(Sleep quality: code 1 if good, code 2 if common, code 3 if poor, code 4 if poor with sleep pill)  Postmenopausal women  Score= 0.346*(Age) + 0.935*(BMI) – 0.075*(AGEMEN) + 0.141*( AGEFLB) – 0.184*(Parity) – 0.110*(breast feeding: code 1 if yes, code 0 if no) – 0.090 (Oral Contraceptive: code 0 if yes, code 1 if no) – 0.710*( Menopausal hormone therapy: code 1 if no, code 0 if yes) + 0.844*( NUMREL_BR) + 0.296*( NBIOPS) + 0.238* (Light at night: code 1 if dark, code 2 if few light, code 3 if little bright) – 0.122*( Sleep quality: code 1 if good, code 2 if common, code 3 if poor, code 4 if poor with sleep pill) | “Light at night” and “Number of previous breast biopsies” were not available, hence excluded. |
| Maas, 2016  (Europe, Australia and the USA) (Maas et al., 2016) | AGEMEN [0: ≤11, -0.0305: 12, -0.0834: 13, -0.1054: 14, -0.0834: 15, -0.1393: ≥16]  Age at menopause [0: ≤40, -0.0202: 41-45, -0.0101: 46-47, 0.0583: 48, 0.1398: 49-50, 0.1398: 51, 0.2151: 52, 0.2546: 53, 0.2469: 54-55, 0.1222: ≥56]  AGEFLB [0: ≤19, 0.0583: 20-22, 0.0296: 23, 0.0100: 24-25, 0.1906: 26-27, 0.3365: 28-30, 0.3148: 31-34, 0.3716: 35-38, 0.2927: ≥39]  Parity [0: nulliparous, -0.1625: 1, -0.2614: 2, -0.2877: 3, -0.3425: ≥4]  Alcohol consumption (drinks/week) [0: 0, -0.0762: ≤0.4, 0.0100: 0.4-0.8, 0.0488: 0.8-1.5, 0: 1.5-3.2, 0.0583: 3.2-5.7, 0.1484: 5.7-9.8, 0.2151: ≥9.8]  Height [0: ≤1.55, 0.1133: 1.55-1.57, 0.1310: 1.57-1.60, 0.1570: 1.60-1.61, 0.0953: 1.61-1.63, 0.2070: 1.63-1.65, 0.1823: 1.65-1.66, 0.2700: 1.66-1.68, 0.1989: 1.68-1.71, 0.2776: ≥1.71]  BMI [0: ≤21.5, 0.0677: 21.5-23.0, 0.0862: 23.0-24.2, 0.1655: 24.2-25.3, 0.1906: 25.3-26.5, 0.1222: 26.5-27.8, 0.2776: 27.8-29.3, 0.3436: 29.3-31.4, 0.3920: 31.4-34.6, 0.4187: ≥34.6]  MHT [0: no, 0.5365: premenopausal, 0.3221: current] | “Post-menopausal BMI” was modified to “BMI”, including both pre- and post- menopausal women |

Supplementary Table 3. (Continued)

| Study  (Country) | Variables, score points and categories | Information on variable availability/modification in ESTHER |
| --- | --- | --- |
| Dierssen-Sotos, 2018  (Spain) (Dierssen-Sotos et al., 2018) | Height [0: ≤1.55, -0.0305: 1.55-1.57, -0.1165: 1.57-1.60, 0.0488: 1.60-1.61, -0.1393: 1.61-1.63, -0.1508: 1.63-1.65, -0.0202: 1.65-1.66, 0: 1.66-1.68, -0.2614: 1.68-1.71, 0.1222: ≥1.71]  BMI [0: ≤21.5, -0.0513: 21.5-23.0, 0.1484: 23.0-24.2, 0.2151: 24.2-25.3, 0.3988: 25.3-26.5, 0.4383: 26.5-27.8, 0.5710: 27.8-29.3, 0.4700: 29.3-31.4, 0.3075: 31.4-34.6, 0.3365: ≥34.6]  Alcohol consumption [0: 0, -0.0619: ≤0.4, 0.0296: 0.4-0.8, -0.2107: 0.8-1.5, -0.1165: 1.5-3.2, -0.1863: 3.2-5.7, -0.1985: 5.7-9.8, 0.0100: ≥9.8]  AGEMEN [0: ≤11, 0.0488: 12, 0.1906: 13, 0.1570: 14, 0.1740: 15, 0.4121: ≥16]  Age at menopause [0: ≤40, -0.1863: 41-45, 0.4574: 46-47, -0.1863: 48, 0.0953: 49-50, 0.6523: 51, 0.2776: 52, 0.5306: 53, 0.3001: 54-55, 0.4886: ≥56]  AGEFLB [0: ≤19, 0.0488: 20-22, -0.1054: 23, -0.1625: 24-25, -0.1508: 26-27, -0.1625: 28-30, -0.1165: 31-34, -0.0202: 35-38, 0.1398: ≥39]  Parity [0: nulliparous, 0.1740: 1, -0.0305: 2, -0.0101: 3, -0.2357: ≥4]  Fami_BC [0: no, 0.7839: yes]  Menopausal status [0: post-menopausal, 0.0100: pre-menopausal]  MHT [0: no, 0.01: yes] | All variables available and included. |
| Rudolph, 2018  (Europe, Australia, Canada and the USA) (Rudolph et al., 2018) | AGEMEN [0: ≤10, 0.0583: 11, 0.0296: 12, 0.0677: 13, 0: 14, 0.0100: 15, -0.1625: ≥16]  Parity [0: nulliparous, -0.2390: parous]  AGEFLB [0: ≤20, -0.0408: 20-25, 0.0677: 25-30, 0.2070: >30]  Alcohol consumption (g/day) [0: 0, 0.0100: <2.5, 0.0677: 2.5-7.5, 0.2231: 7.5-12.5, 0.0488: 12.5-17.5, 0.0198: 17.5-22.5, 0.3001: ≥22.5]  Height [0: <1.58, 0.0677: 1.58-1.62, 0.1823: 1.65-1.68, 0.2469: ≥1.68]  BMI [0: <18.5, -0.1054: 18.5-25, 0: 25-30, 0.2311: ≥30]  MHT: [0: no, 0.3920: yes] | All variables available and included. |

Abbreviation: AGEMEN: Age at menarche; NBIOPS: Number of previous breast biopsies; AGEFLB: Age at first live birth; BMI: Body mass index; NUMREL_BR: Number of first-degree relatives with breast cancer; NUMREL_Ca: Number of first-degree relatives with any cancer; BR_INFLAM: Breast inflammation; BMI: Body mass index; MHT: Menopausal hormone therapy; Fami_BC: Family history of breast cancer; PA: Physical activity.

Supplementary Table 4. Performance of risk scores with respect to time to diagnosis in cases

| Median time to diagnosis | AUC (95% CI) | | | |
| --- | --- | --- | --- | --- |
|  | MRS^a^ | GRS | ERS^b^ | Combined risk score^c^ |
|  |  |  |  |  |
| ≤ 7 years  (n= 52 cases) | 0.553  (0.495-0.598) | 0.603  (0.567-0.649) | 0.602  (0.557-0.648) | 0.630  (0.576-0.663) |
| > 7 years  (n= 49 cases) | 0.556  (0.509-0.599) | 0.628  (0.588-0.667) | 0.586  (0.535-0.634) | 0.625  (0.578-0.672) |

^a^ 423-CpGs score

^b^ Dierssen-Sotos Score

^c^MRS + GRS + ERS

Abbreviation: MRS, Methylation Risk Score; GRS, Genetic Risk Score; ERS, Environmental Risk Score.

Supplementary Figure Legends

Supplementary Figure 1. Correlation among risk scores

(A) Correlation between MRS (423-CpGs) and GRS

(B) Correlation between MRS (423-CpGs) and ERS

(C) Correlation between GRS and ERS

Supplementary Figure 1.


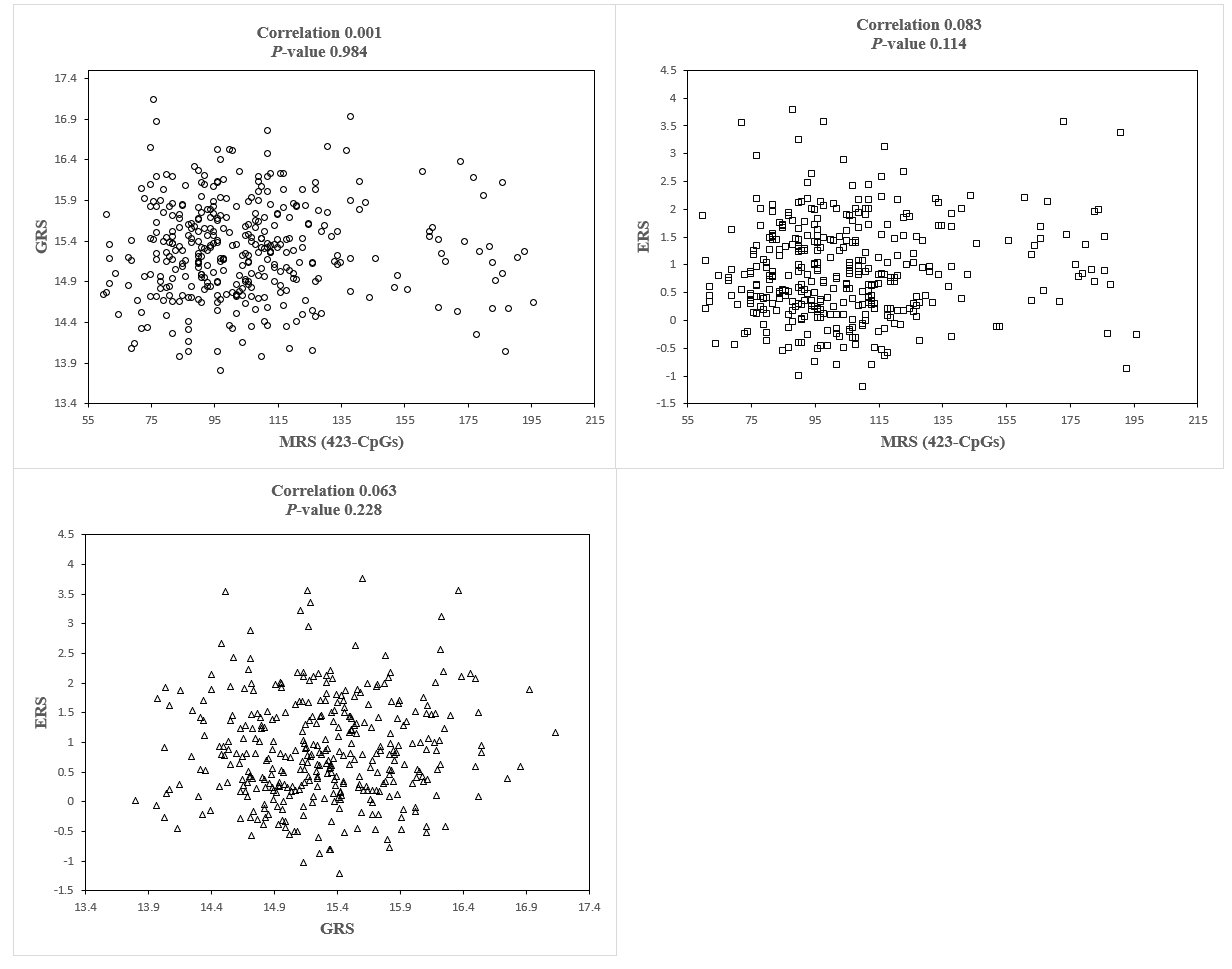

Supplement: Supplementary file 1 — Table S1 . Individual association of the previously identified CpGs with BC risk in the ESTHER study and reported methylation direction. Table S2 . SNPs used for genetic risk score construction. Table S3 . Environmental risk scores from previous studies and availability/modification of related variables in ESTHER. Table S4 . Performance of risk scores with respect to time to diagnosis in cases. Fig. S1. Correlation among risk scores. [file MOL2-14-42-s001.docx]
